# Relationship Between GLIM‐Defined Malnutrition and Postoperative Outcomes After Curative Resection in Patients With Gastroenterological Cancer: Update Systematic Review and Meta‐Analysis

**DOI:** 10.1002/ags3.70173

**Published:** 2026-01-27

**Authors:** Ryota Matsui, Jun Watanabe, Kazuma Rifu, Souya Nunobe, Noriyuki Inaki

**Affiliations:** ^1^ Department of Gastroenterological Surgery Cancer Institute Ariake Hospital Tokyo Japan; ^2^ Department of Gastrointestinal Surgery/Breast Surgery, Graduate School of Medical Science Kanazawa University Kanazawa City Ishikawa Japan; ^3^ Department of Surgery, Division of Gastroenterological, General and Transplant Surgery Jichi Medical University Shimotsuke City Tochigi Japan; ^4^ Center for Community Medicine Jichi Medical University Shimotsuke City Tochigi Japan

**Keywords:** gastroenterological cancer, malnutrition, overall survival, postoperative complication, prognosis

## Abstract

**Background:**

In cancer patients, malnutrition worsens postoperative outcomes, with increased complications and poor prognosis. We aimed to update the impact of malnutrition, as defined by the Global Leadership Initiative on Malnutrition (GLIM) criteria, on postoperative outcomes in patients with gastroenterological cancer after curative resection.

**Methods:**

We identified observational studies published from inception to April 2, 2025. A systematic review and random‐effects meta‐analysis were performed on studies including adult patients (age > 18 years) with gastroenterological cancer who received surgical treatment and had nutritional status assessments based on GLIM criteria. The primary outcomes were overall survival (OS) and overall postoperative complications, which were defined as events with a Clavien–Dindo (CD) grade ≥ II that occurred within 30 days after surgery. Hazard ratios and relative risk ratios for OS and postoperative complications, respectively, with 95% confidence intervals were pooled. The protocol has been published in PROSPERO (CRD42023434267).

**Results:**

Twenty‐five studies (28 reports comprising 10 942 patients) were included in the qualitative and quantitative syntheses. Compared with the absence of malnutrition, GLIM‐defined malnutrition probably worsens OS (hazard ratio: 1.88, 95% concordance interval: 1.62–2.18, certainty of the evidence: low) and increases postoperative complications (relative risk ratio: 1.57, 95% concordance interval: 1.34–1.84, certainty of the evidence: low). The risk of bias in each study was moderate or high.

**Conclusions:**

GLIM‐defined malnutrition probably worsens OS and increases the risk of postoperative complications in patients with gastroenterological cancer after surgery.

## Graphical abstract

GLIM‐defined malnutrition probably worsens overall survival in patients with gastroenterological cancer after surgery.

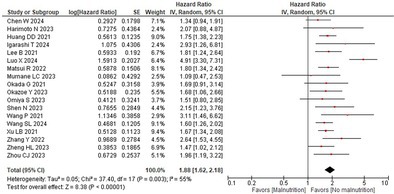

## 1 Introduction

In patients with cancer, malnutrition worsens postoperative outcomes, including increased postoperative complications and poor postoperative prognosis [1, 2]. Nutritional conditioning and nutritional therapy for 7–10 and 10–14 days are recommended in patients with mild and severe malnutrition, respectively, to reduce postoperative complications [1, 2]. However, there is no consensus on standard criteria that indicate the need for nutritional interventions, and opinions remain controversial as the targets of nutritional interventions have not been standardized in clinical trials. Therefore, universal standards for nutritional intervention are required in nutritional intervention trials.

The Global Leadership Initiative on Malnutrition (GLIM) criteria was published in 2019 as a global consensus on malnutrition [3] that comprises body mass index (BMI), body weight loss (BWL) rate, and reduced muscle mass, which were previously used as criteria for nutritional interventions based on severity ratings that aided the determination of the severity of malnutrition. As the procedures for diagnosing malnutrition and identifying patients for preoperative nutritional intervention vary between countries and institutions, it is important to standardize the general diagnostic criteria of malnutrition. Therefore, the establishment of diagnostic criteria by a global consensus can be advantageous for providing a universal assessment criterion.

Due to the small number of studies, the relationship between GLIM criteria‐defined malnutrition and postoperative outcomes in patients with cancer have not been fully investigated. Previous systematic review found few prospective studies on malnutrition defined by GLIM criteria [4]. While there were studies on upper gastrointestinal cancers such as gastric and esophageal cancer, there were few studies on colorectal cancer or hepatobiliary‐pancreatic cancer [4]. Therefore, updated systematic review using the GLIM criteria is necessary to ensure applicability across multiple cancer types. According to the GLIM criteria, BMI, BWL rate, and skeletal muscle mass were used to determine the severity of malnutrition. A BWL of > 10% or reduced skeletal muscle mass has been reported to increase postoperative complications [5, 6, 7, 8]. If this assumption is correct, the postoperative outcomes are likely to be worse in patients with severe malnutrition than in those with moderate malnutrition. However, the relationship between the severity of GLIM‐defined malnutrition and outcomes remains unclear.

In this latest meta‐analysis, we aimed to update the impact of GLIM criteria on postoperative outcomes in patients with gastroenterological cancer after curative resection, especially in a wide range of cancer types. We hypothesized that more severe malnutrition would be associated with worse short‐ and long‐term outcomes and more postoperative complications.

## 2 Methods

The Preferred Reporting Items for Systematic Review and Meta‐Analysis 2020 (PRISMA‐2020; Appendix S1) [9] were followed in the conduct and reporting of this study. PROSPERO has released the protocol (CRD42023434267).

In addition to searching the Cochrane Central Register of Controlled Trials (Cochrane Library), MEDLINE (PubMed), and EMBASE (Dialog) (Appendix S2), we additionally searched through the World Health Organization International Clinical Trials Platform Search Portal (ICTRP) and Clinical Trials.gov databases for ongoing or unpublished trials (Appendix S3). International guidelines [10, 11, 12, 13, 14, 15, 16, 17, 18, 19, 20, 21, 22, 23, 24, 25, 26, 27], reference lists, and publications mentioning relevant studies were also examined, as were study reference lists.

### 2.1 Including or Excluding Criteria

We included observational studies of patients, with gastroenterological cancer, and aged > 18 years who underwent surgical treatment and with GLIM criteria‐based nutritional status assessment. Studies with benign disease, mixed populations with cancer or benign disease, nonsurgical therapy, and nutritional evaluations that did not meet the GLIM criteria were not included. We found studies that were published between the beginning and April 2, 2025. The exposure variable was malnutrition as described by GLIM. Patients with cancer who did not have malnutrition as characterized by GLIM made up the control group. Patients who received surgical treatment and whose nutritional status was evaluated using the GLIM criteria were included in qualitative investigations, but only patients with outcome data were included in quantitative syntheses (meta‐analyses).

### 2.2 Definition of Outcomes

Overall survival (OS) and postoperative complications—defined as occurrences with a Clavien‐Dindo (CD) grade ≥ II that occurred within 30 days following surgery—were the primary outcomes. Relapse‐free survival (RFS) and length of hospital stay following surgery were secondary outcomes. We determined the percentage of anastomotic leakage, postoperative pneumonia, infectious complications, severe complications with CD grade ≥ III, and postoperative mortality in the sub‐analysis of the complications.

### 2.3 Extracting Data and Evaluating Its Quality

After screening the abstracts and titles, two independent reviewers (RM and JW) determined each reviewer's eligibility by reading the entire manuscript. Following data extraction, the same reviewers (RM and JW) independently assessed the certainty of evidence (COE) and the risk of bias using Quality In Prognosis Studies (QUIPS) in accordance with the Grading of Recommendations, Assessment, Development, and Evaluation (GRADE) approach [28]. A third reviewer (KR) served as the mediator after the two reviewers' disagreements could not be settled through discussion. When pertinent information was lacking, the original writers were contacted.

### 2.4 Analysis of Statistics

We combined the relative risk ratios (RR) and 95% CI for postoperative complications and postoperative mortality, the mean differences (MD) and 95% CI for postoperative hospital stay, and the hazard ratios (HR) and 95% CI for OS and RFS.

As much as practicable, an intention‐to‐treat analysis was conducted for dichotomous data. The *I* ^2^ statistic and visual examination of forest plots were used to assess statistical heterogeneity [29]. When significant heterogeneity was observed (*I* ^2^ > 50%), the cause was evaluated. We looked for reporting bias by searching the clinical trial registry systems (ClinicalTrials.gov and ICTRP). Upon inclusion of ≥ 10 eligible studies in the meta‐analysis, potential publication bias was evaluated by visual inspection of the funnel plot in accordance with the Cochrane handbook criteria [29].

We conducted meta‐analyses using a random‐effects model using Review Manager 5.4.2 and STATA SE16 (version 16.1, Stata Corporation, College Station, TX, USA). According to the Cochrane handbook, we hypothesized that the true effect would be minimal due to individual differences between trials and several unmeasured or unknown elements in the human body [29].

Subgroup analyses by location (e.g., head and neck, lung, gastroenterology, urology, or gynecology), location (gastrointestinal (GI) or hepatopancreatobiliary (HPB)), cancer type (esophageal or gastric or colorectal or hepatobiliary), severity of malnutrition (moderate or severe), surgical approach (minimally invasive surgery [MIS] or open surgery), and method of assessed skeletal muscle mass (computed tomography [CT], magnetic resonance imaging (MRI), bioelectrical impedance analysis (BIA), dual energy X‐ray absorptiometry (DXA), or calf circumference) were carried out when enough information was available to clarify the impact of effect modifiers on the results. The surgical approach was allocated to the group with the highest implementation rate. The studies that did not assess muscle mass were excluded using sensitivity analysis.

### 2.5 Certainty of the Evidence (COE)

We compiled the results for OS, RFS, and rates of overall postoperative complications, severe complications, infectious complications, anastomotic leakage, postoperative pneumonia, postoperative mortality, and postoperative hospital stay based on the Cochrane Handbook [29]. The GRADE method includes the rating of COE as a prognostic factor in a summary of the results for all outcomes [29]. For observational study evidence, we began with “high” COE [29]. We reduced the grade from “high” COE in any domain where there were significant issues.

## 3 Results

### 3.1 Study Selection

**FIGURE 1:**
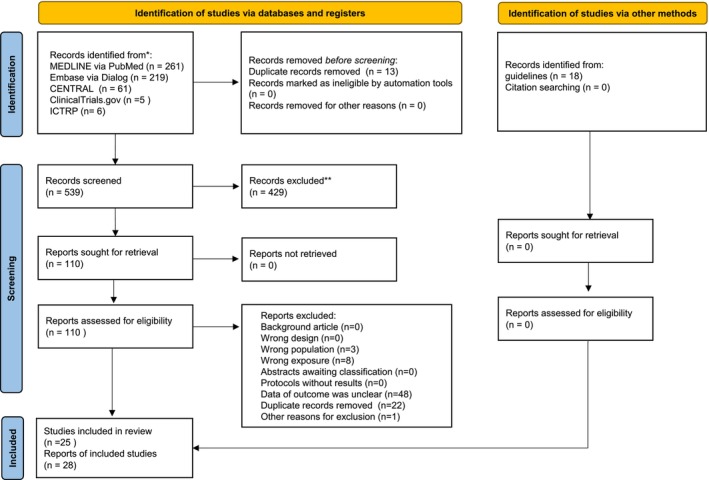
PRISMA 2020 flow diagram of this study.

The PRISMA flowchart is presented in Figure 1. A search of 539 records was conducted between the period of their publication and April 2, 2025.25 studies (28 reports, 10 942 patients) were included in the qualitative [30, 31, 32, 33, 34, 35, 36, 37, 38, 39, 40, 41, 42, 43, 44, 45, 46, 47, 48, 49, 50, 51, 52, 53, 54, 55, 56, 57] and quantitative syntheses following screening. Our search found up no unpublished data or current research, despite our following the Cochrane Handbook's instructions to include them in our systematic review. Qualitative and quantitative syntheses did not include the studies that are listed in Appendix S4. Insufficient outcome data (*n* = 48), inaccurate exposure (*n* = 8), inappropriate population (*n* = 3), and other reasons (*n* = 1) were the reasons for exclusion.

### 3.2 Study and Participant Characteristics

Table 1 summarizes the characteristics of the 25 studies included in the quantitative synthesis. Five studies were prospective cohort studies and 20 were retrospective cohort studies. Eighteen studies focused on GI cancer, five on HPB cancer, and two on mixed GI and HPB cancers. Seventeen studies assessed skeletal muscle mass using CT, three used anthropometry, two used BIA, and three did not. The pooled proportion of malnutrition was 40.0% (95% CI: 33.0%–47.0%; Figure S1).

**TABLE 1:** Summary of reports on GLIM criteria‐defined malnutrition and outcomes.

Author (Year)	Country	Design	Cancer type	Sample size	Age (mean)	BMI (mean)	Male (%)	Prevalence (%) (malnutrition)	Muscle mass assessment	Cut off
Huang DD (2021) [30] Huang DD (2022) [31]	China	Retrospective	Gastric	1359	66.0	22.5	73.6	28.2	CT	Men: 40.8 cm^2^/m^2^ Women: 34.9 cm^2^/m^2^
Kakavas S (2020) [32]	Greece	Prospective	GI cancer	218	70.1	NA	41.3	33.0	CC	31 cm
Lee B (2021) [33]	Korea	Retrospective	Pancreas	228	NA	NA	NA	32.9	NA	
Okada G (2021) [34]	Japan	Retrospective	Esophageal	117	63.8	21.0	75.2	43.6	BIA	Men: 6.1 kg/m^2^ Women: 5.0 kg/m^2^
Wang P (2021) [35]	China	Retrospective	Esophageal	189	65.1	22.9	68.8	75.7	BIA	Men: 9.87 kg/m^2^ Women: 7.15 kg/m^2^
Xu LB (2021) [36]	China	Prospective	Gastric	895	65.6	22.3	74.0	38.3	CT	Men: 40.8 cm^2^/m^2^ Women: 34.9 cm^2^/m^2^
Yin L (2021) [37]	China	Retrospective	Esophageal	360	64.1	22.4	80.8	33.3	CC	Men: 30 cm Women: 29 cm
Liu Y (2023) [38]	China	Prospective	Esophageal	182	NA	NA	79.7	48.4	CT	Men: 52.4 cm^2^/m^2^ Women: 38.5 cm^2^/m^2^
Matsui R (2022) [39] Matsui R (2022) [40] Matsui R (2024) [41]	Japan	Retrospective	Gastric	512	67.9	22.8	65.6	33.6	CT	Men: 41.9 cm^2^/m^2^ Women: 34.0 cm^2^/m^2^
Murnane LC (2023) [42]	Australia	Retrospective	Gastric Esophageal	108	66.4	25.9	75.0	72.2	CT	Men: 52.4 cm^2^/m^2^ Women: 38.5 cm^2^/m^2^
Okazoe Y (2023) [43]	Japan	Retrospective	Bile duct	166	71.0	21.6	65.1	78.9	CT	Mean −1SD/−2SD
Tan S (2022) [44]	China	Retrospective	GI/HPB	1115	62.6	23.4	66.8	35.9	CT	Men: 43.1 cm^2^/m^2^ Women: 37.8 cm^2^/m^2^
Wobith M (2022) [45]	Germany	Retrospective	GI/HPB	260	70.2	NA	56.5	25.4	CT	Men: 52.4 cm^2^/m^2^ Women: 38.5 cm^2^/m^2^
Zhang Y (2022) [46]	China	Retrospective	Gastric	182	62.0	23.3	74.2	36.3	CT	Men: 52.4 cm^2^/m^2^ Women: 38.5 cm^2^/m^2^
Zheng HL (2022) [47]	China	Retrospective	Gastric	1121	61.0	21.9	76.2	69.2	NA	
Omiya S (2023) [48]	Japan	Retrospective	HCC	293	70.0	23.1	83.6	55.6	CT	Men: 45.0 cm^2^/m^2^ Women: 34.0 cm^2^/m^2^
Harimoto N (2023) [49]	Japan	Retrospective	HCC	176	70.4	23.6	82.4	26.7	CT	Men: 42.0 cm^2^/m^2^ Women: 38.0 cm^2^/m^2^
Shen N (2023) [50]	China	Retrospective	Colorectal	385	73.0	22.6	60.0	30.7	NA	
Zhou CJ (2023) [51]	China	Retrospective	Rectal	624	65.0	NA	60.7	25.0	CT	Men: 40.8 cm^2^/m^2^ Women: 34.9 cm^2^/m^2^
Sun S (2023) [52]	China	Retrospective	Gastric	220	61.8	22.2	75.5	30.0	CT	Men: 40.8 cm^2^/m^2^ Women: 34.9 cm^2^/m^2^
Chen W (2024) [53]	China	Retrospective	Colorectal	850	64.0	25.1	65.7	12.4	CT	Men: 40.8 cm^2^/m^2^ Women: 34.9 cm^2^/m^2^
Wang SL (2024) [54]	China	Prospective	Gastric	1420	65.5	22.5	73.6	28.8	CT	Men: 40.8 cm^2^/m^2^ Women: 34.9 cm^2^/m^2^
Igarashi T (2024) [55]	Japan	Retrospective	Biliary tract	114	72.6	23.4	62.3	41.2	CT	Men: 42.0 cm^2^/m^2^ Women: 38.0 cm^2^/m^2^
Yıldız Kopuz TN (2024) [56]	Turkey	Prospective	Colorectal	121	62.3	27.1	62.8	45.5	CT	Men: 53.0 (BMI ≥ 25 kg/m^2^) 43.0 (BMI < 25 kg/m^2^) Women: 41.0 cm^2^/m^2^
Luo X (2024) [57]	China	Retrospective	Gastric	301	64.8	21.8	70.1	45.5	CC	Men: 33 cm Women: 32 cm

Abbreviations: BIA, bioelectrical impedance analysis; CC, calf circumference; CT, computed tomography; GI, gastrointestinal; GLIM, global leadership initiative on malnutrition; HPB, hepatobiliary pancreas; NA, not applicable.

### 3.3 Risk of Bias

The selected studies' risk of bias is compiled in Appendix S5. In terms of OS, the risk of bias was low or moderate for statistical analyses, prognostic factor measurement, and study participation, while it was low or high for outcome measurements. Confounding variables and research attrition had moderate to high bias risks.

Prognostic factor measurement had a low or moderate risk of bias, study confounding and statistical analysis had a moderate or high risk of bias, and study participation, study attrition, and outcome measurements had a low risk of bias with regard to postoperative complications.

### 3.4 Results of Meta‐Analysis

The results of the GRADE approach are compiled in Table 2. However, neither cancer‐specific nor other‐cause survival outcomes have been recorded. Since none of the studies employed imputed statistics and all used data on outcomes computed using the original GLIM‐defined malnutrition, we were unable to do sensitivity analysis.

**TABLE 2:** Summary of findings.

Relationship between GLIM‐defined malnutrition and postoperative outcomes after curative resection in patients with cancer				
Study population: adults, exposure: with malnutrition defined by the GLIM criteria, comparison: without malnutrition				
Outcomes	Relative effect (95% CI)	Patients number (Studies)	Certainty of the evidence (GRADE)	Comments
Overall survival	HR 1.88 (1.62 to 2.18)	8466 (18 non‐RCT)	⊕⊕⊝⊝ Low^a^, ^b^	GLIM‐defined malnutrition probably worsens overall survival
Relapse‐free survival	HR 1.63 (1.38 to 1.93)	2893 (8 non‐RCT)	⊕⊕⊕⊝ Moderate^a^	GLIM‐defined malnutrition probably worsens relapse‐free survival
Total postoperative complications	RR 1.57 (1.34 to 1.84)	8973 (18 non‐RCT)	⊕⊕⊝⊝ Low^a^, ^b^	GLIM‐defined malnutrition may increase total postoperative complications
Severe complications	RR 1.72 (1.28 to 2.32)	8295 (17 non‐RCT)	⊕⊕⊕⊝ Moderate^a^	GLIM‐defined malnutrition probably increases severe complications
Infectious complications	RR 1.47 (1.21 to 1.80)	7287 (12 non‐RCT)	⊕⊕⊕⊝ Moderate^a^	GLIM‐defined malnutrition probably increases infectious complications
Anastomotic leakage	RR 1.37 (1.04 to 1.79)	6377 (11 non‐RCT)	⊕⊕⊕⊝ Moderate^a^	GLIM‐defined malnutrition probably increases anastomotic leakage
Postoperative pneumonia	RR 1.92 (1.37 to 2.70)	6377 (11 non‐RCT)	⊕⊕⊝⊝ Low^a^, ^b^	GLIM‐defined malnutrition may increase postoperative pneumonia
Mortality	RR 1.34 (0.78 to 2.33)	4501 (9 non‐RCT)	⊕⊕⊝⊝ Low^a^, ^c^	GLIM‐defined malnutrition may not increase mortality
Postoperative hospital stays	MD 1.52 (0.81 to 2.24)	6943 (16 non‐RCT)	⊕⊕⊕⊝ Moderate^a^	GLIM‐defined malnutrition probably increases postoperative hospital stay

Abbreviations: CI, confidence interval; GLIM, global leadership initiative on malnutrition; HR, hazard ratio; RCT, randomized control trials; RR, risk ratio.

^a^ Downgraded by one point because of moderate or high risk of bias associated with study attrition, prognostic factor measurement, study confounding, and statistical analysis.

^b^ Downgraded one point because of inconsistency due to substantial heterogeneity.

^c^ Downgraded one point because of inconsistency of forest plot.

### 3.5 Overall Survival

**FIGURE 2:**
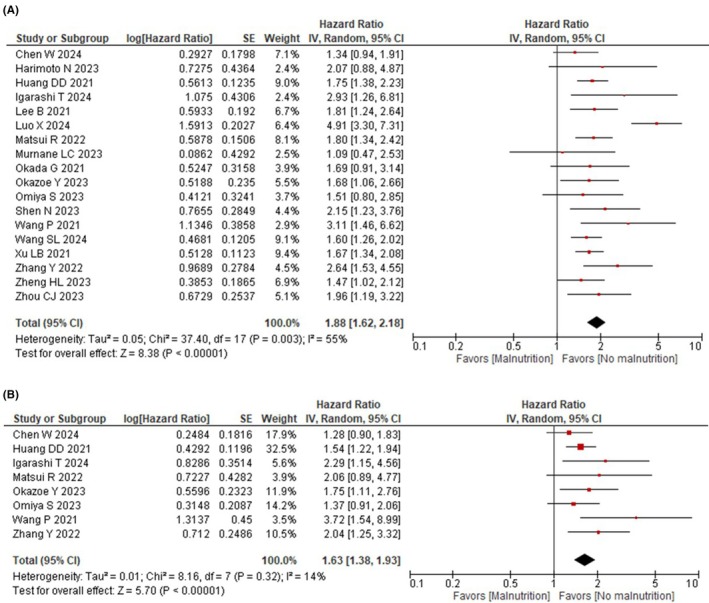
Forest plot of long‐term outcomes according to GLIM‐defined malnutrition. (a) For overall survival. (b) For relapse‐free survival.

Eighteen studies reported OS. According to the meta‐analysis, GLIM‐defined malnutrition probably worsens OS (HR: 1.88, 95% CI: 1.62–2.18, *I* ^2^ = 55%, *n* = 18, COE: low, Figure 2a).

Subgroup analysis showed no significant intergroup differences according to malnutrition severity (Figure S2a), cancer type (Figure S2b,c), presence of assessing skeletal muscle mass (Figure S2d), method of assessing skeletal muscle mass (Figure S2e), or surgical approach (Figure S2f). Due to the limited amount of data, no additional subgroup analyses were carried out.

In the sensitivity analysis excluding studies without muscle mass assessment, the effect size estimates were largely consistent with the primary analysis (HR: 1.92, 95% CI: 1.61–2.29, Figure S2g).

### 3.6 Relapse‐Free Survival

Eight studies reported RFS. According to the meta‐analysis, GLIM‐defined malnutrition probably worsens RFS (HR: 1.63, 95% CI: 1.38–1.93, *I* ^2^ = 14%, *n* = 8; COE: moderate, Figure 2b).

The subgroup analysis showed no significant intergroup difference according to malnutrition severity (Figure S3a). Due to the limited amount of data, no additional subgroup analyses were carried out.

In the sensitivity analysis excluding studies without muscle mass assessment, the effect size estimates were largely consistent with the primary analysis (HR: 1.63, 95% CI: 1.38–1.93, Figure S3b).

### 3.7 Total Postoperative Complications

**FIGURE 3:**
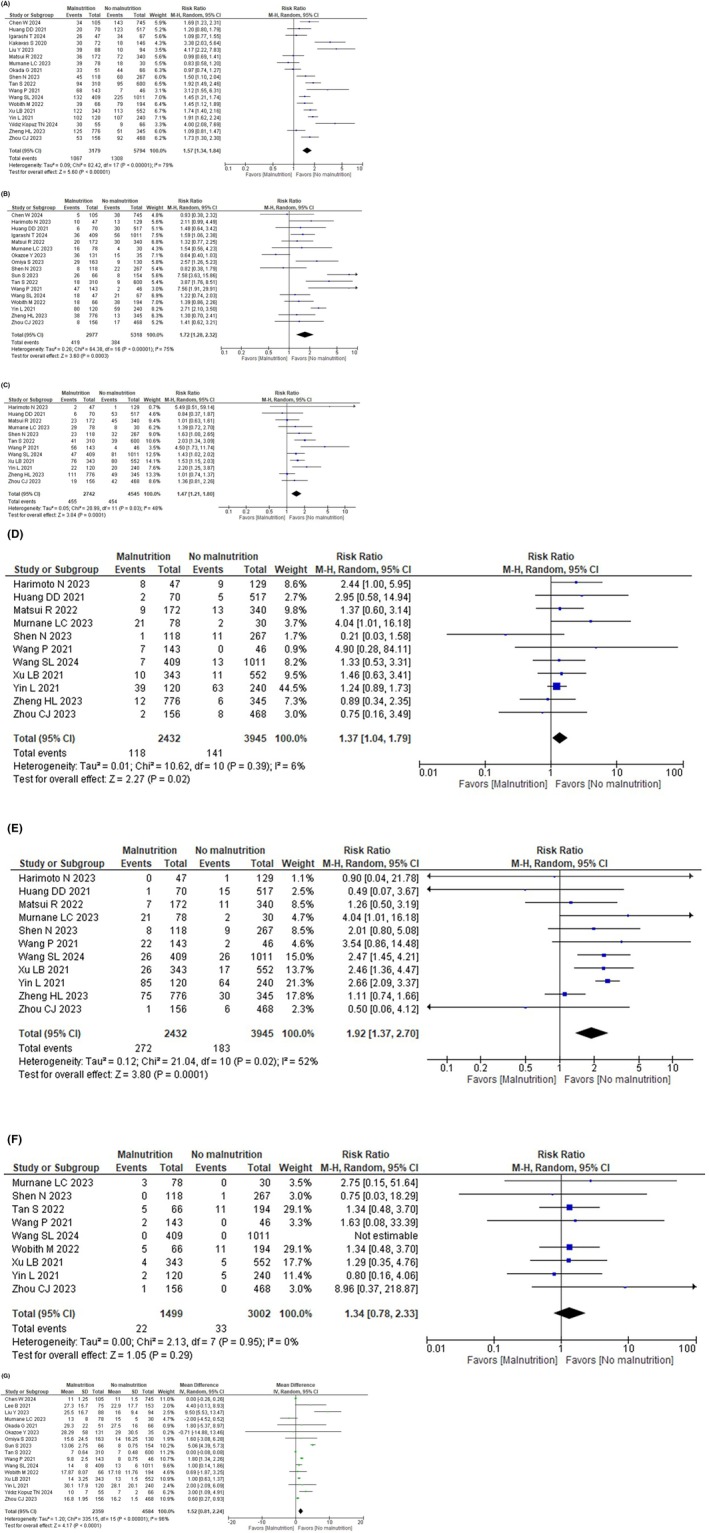
Forest plot of short‐term outcomes according to GLIM‐defined malnutrition. (a) For total number of postoperative complications. (b) For severe complications. (c) For infectious complications. (d) For anastomotic leakage. (e) For postoperative pneumonia. (f) For mortality. (g) For postoperative hospital stay.

Eighteen studies reported postoperative complications. According to the meta‐analysis, GLIM‐defined malnutrition probably increases the total postoperative complications (RR: 1.57, 95% CI: 1.34–1.84, *I* ^2^ = 79%, *n* = 18, COE: low, Figure 3a).

Subgroup analysis showed no significant intergroup differences according to the severity of malnutrition (Figure S4a), cancer type (Figure S4b), presence of assessing skeletal muscle mass (Figure S4c), method of assessing skeletal muscle mass (Figure S4d), or surgical approach (Figure S4e). Due to the limited amount of data, no additional subgroup analyses were carried out.

In the sensitivity analysis excluding studies without muscle mass assessment, the effect size estimates were largely consistent with the primary analysis (RR: 1.62, 95% CI: 1.36–1.93, Figure S4f).

### 3.8 Severe Complications

Seventeen studies reported severe complications. According to the meta‐analysis, GLIM‐defined malnutrition probably increases the risk of severe complications (RR: 1.72, 95% CI: 1.28–2.32, *I* ^2^ = 75%, *n* = 17, COE: moderate, Figure 3b).

Subgroup analysis of surgical approach showed increase severe complications in the MIS group compared with the open surgery group (*p* = 0.04; Figure S5a). Subgroup analysis of cancer type showed significant difference (*p* = 0.04; Figure S5b). Subgroup analysis showed no significant intergroup differences according to the severity of malnutrition (Figure S5c), cancer type (GI or HPB; Figure S5d), presence of assessing skeletal muscle mass (Figure S5e), method of assessing skeletal muscle mass (Figure S5f). Due to the limited amount of data, no additional subgroup analyses were carried out.

In the sensitivity analysis excluding studies without muscle mass assessment, patients with GLIM‐defined malnutrition who underwent muscle mass measurement had a higher risk of severe complications (RR: 1.84, 95% CI: 1.34–2.54, Figure S5g).

### 3.9 Infectious Complications

Twelve studies reported infectious complications. According to the meta‐analysis, GLIM‐defined malnutrition probably increases infectious complications (RR: 1.47, 95% CI: 1.21–1.80, *I* ^2^ = 48%, *n* = 12, COE: moderate, Figure 3c).

Subgroup analysis showed no significant intergroup difference according to the severity of malnutrition (Figure S6a), cancer types (Figure S6b), surgical approach (Figure S6c), or presence of assessing skeletal muscle mass (Figure S6d). Due to the limited amount of data, no additional subgroup analyses were carried out.

In the sensitivity analysis excluding studies without muscle mass assessment, the effect size estimates were largely consistent with the primary analysis (RR: 1.55, 95% CI: 1.25–1.93, Figure S6e).

### 3.10 Anastomotic Leakage

Eleven studies reported anastomotic leakages. According to the meta‐analysis, GLIM‐defined malnutrition probably increases the risk of anastomotic leakages (RR: 1.37, 95% CI: 1.04–1.79, *I* ^2^ = 6%, *n* = 11, COE: moderate, Figure 3d).

The subgroup analysis showed no significant intergroup differences according to the severity of malnutrition (Figure S7a), cancer type (Figure S7b), surgical approach (Figure 7c), or presence of assessing skeletal muscle mass (Figure S7d). Due to the limited amount of data, no additional subgroup analyses were carried out.

In the sensitivity analysis excluding studies without muscle mass assessment, the effect size estimates were largely consistent with the primary analysis (RR: 1.43, 95% CI: 1.11–1.83, Figure S7e).

### 3.11 Postoperative Pneumonia

Eleven studies reported postoperative pneumonia. According to the meta‐analysis, GLIM‐defined malnutrition may increase postoperative pneumonia (RR: 1.92, 95% CI: 1.37–2.70, *I* ^2^ = 52%, *n* = 11, COE: low, Figure 3e).

The subgroup analysis showed no significant intergroup differences according to the severity of malnutrition (Figure S8a), cancer type (Figure S8b), surgical approach (Figure 8c), or presence of assessing skeletal muscle mass (Figure S8d). Due to the limited amount of data, no additional subgroup analyses were carried out.

In the sensitivity analysis excluding studies without muscle mass assessment, patients with GLIM‐defined malnutrition who underwent muscle mass measurement had a higher risk of postoperative pneumonia (RR: 2.44, 95% CI: 1.96–3.03, Figure S8e).

### 3.12 Mortality

Nine studies reported postoperative pneumonia. According to the meta‐analysis, GLIM‐defined malnutrition may not increase the mortality risk (RR: 1.34, 95% CI: 0.78–2.33, *I* ^2^ = 0%, *n* = 9, COE: low, Figure 3f).

Subgroup analysis showed no significant intergroup differences according to cancer type (Figure S9a), surgical approach (Figure S9b), or the method of assessing skeletal muscle mass (Figure S9c). Due to the limited amount of data, no additional subgroup analyses were carried out.

In the sensitivity analysis excluding studies without muscle mass assessment, the effect size estimates were largely consistent with the primary analysis (RR: 1.37, 95% CI: 0.78–2.39, Figure S9d).

### 3.13 Postoperative Hospital Stay

Sixteen studies reported postoperative hospital stay. According to the meta‐analysis, GLIM‐defined malnutrition probably increases the length of postoperative hospital stay (MD: 1.52, 95% CI: 0.81–2.24, *I* ^2^ = 96%, *n* = 16, COE: moderate, Figure 3g).

The subgroup analysis showed no significant intergroup differences according to the severity of malnutrition (Figure S10a), cancer type (Figure S10b,c), surgical approach (Figure S10d), or method of assessing skeletal muscle mass (Figure S10e). Due to the limited amount of data, no additional subgroup analyses were carried out.

In the sensitivity analysis excluding studies without muscle mass assessment, the effect size estimates were largely consistent with the primary analysis (MD: 1.46, 95% CI: 0.74–2.19, Figure S10f).

### 3.14 Publication Bias

Funnel plots were symmetrically visualized, indicating that no publication bias existed in the reporting of OS (Figure S11a), total postoperative complications (Figure S11b), severe complications (Figure S11c), infectious complications (Figure S11d), anastomotic leakage (Figure S11e), postoperative pneumonia (Figure S11f), or postoperative hospital stay (Figure S11g). Funnel plots were not prepared for RFS and mortality because the number of references was less than 10, according to the Cochrane Handbook [29].

## 4 Discussion

The results of this systematic review and meta‐analysis of 25 studies and 10 942 patients revealed that GLIM‐defined malnutrition may worsen OS and RFS in surgically treated patients with gastroenterological cancer and cause a high rate of postoperative complications, including total, severe, infectious complications, anastomotic leakage, and postoperative pneumonia.

In this study, malnutrition as defined by the GLIM was significantly associated with poor OS in patients with gastroenterological cancer undergoing surgery, and the more severe the malnutrition, the poorer the prognosis. A previous meta‐analysis has reported that malnutrition, using the Mini Nutritional Assessment, was associated with a 3‐ to 8‐fold higher mortality rate in patients with cancer [58]. A recent systematic review also demonstrated that malnutrition, as assessed by various validated nutritional tools, is associated with poorer long‐term prognosis (HR 1.87; 95% CI, 1.62–2.17) [59]. However, it must be noted that GLIM is a diagnostic framework for malnutrition rather than a comprehensive prognostic model. The use of global‐consensus‐based diagnostic criteria for malnutrition is important for the development of future clinical trials.

In this study, malnutrition, as defined by the GLIM, worsened RFS in patients with cancer undergoing surgical treatment. This can be attributed to two factors. First, GLIM‐defined malnutrition increased the incidence of postoperative complications, which resulted in poor RFS [60]. The second reason was the impact of chemotherapy. The diagnosis of GLIM‐defined malnutrition included low muscle mass. Low muscle mass intensifies the side effects of postoperative adjuvant and neoadjuvant chemotherapy, making it difficult to continue cancer treatment [61, 62, 63]. Therefore, malnutrition, as defined by the GLIM, may be a useful risk factor for postoperative cancer recurrence. Only one study demonstrated that GLIM‐defined malnutrition is associated with poor compliance with adjuvant chemotherapy [40]. Further research is needed to examine the relationship between the impact of chemotherapy and GLIM‐defined malnutrition.

This systematic review revealed that GLIM‐defined malnutrition is a risk factor for various complications, including total postoperative complications, severe complications, infectious complications, anastomotic leakage, and postoperative pneumonia. Malnutrition, as defined by the GLIM, includes low BMI, high preoperative weight loss, and low muscle mass. Among these, BWL > 10% and low muscle mass have been reported to increase postoperative complications [4, 5, 6, 7, 8]. Systematic reviews have reported increased postoperative pneumonia in patients with low muscle mass and BMI < 18.5 kg/m^2^ but no increase in anastomotic leakage [7, 64]. As GLIM‐defined malnutrition includes poor dietary intake and the presence of inflammation as diagnostic criteria, this condition may facilitate the identification of a wider range of postoperative complications than sarcopenia, which was previously thought to require intervention.

Subgroup analysis by severity of malnutrition showed no significant difference, but the effect size was greater in the severe group. One reason why effect sizes are clearly larger in the severe group but not statistically significant is the limited number of studies comparing outcomes by severity grade. Less than half of studies reported outcomes by severity grade for each specific outcome. Therefore, this meta‐analysis may not be able to clarify the true effect of malnutrition severity on outcomes. To prove this fact, further research summarizing outcomes by severity level is necessary.

The results of this study suggest that a significant number of patients with a preoperative diagnosis of malnutrition, as defined by the GLIM, are eligible for preoperative nutritional intervention. The prevalence of malnutrition varied from 12.4% to 78.9%, which in most studies ranged from 30% to 40% [30, 31, 32, 33, 34, 35, 36, 37, 38, 39, 40, 41, 42, 43, 44, 45, 46, 47, 48, 49, 50, 51, 52, 53, 54, 55, 56, 57]. A systematic review showed that the GLIM criteria had high diagnostic accuracy for malnutrition, with a sensitivity of 0.72 (95% CI, 0.64–0.78) and specificity of 0.82 (95% CI, 0.72–0.88) [65]. However, muscle mass should be measured using an objective index because heterogeneity in diagnostic accuracy can occur if the measurement is not used correctly [65, 66]. Muscle mass measurements should be included in routine clinical practice, while recognizing that there are more undernourished patients who need intervention than surgeons believe.

Sensitivity analysis excluding studies without muscle mass assessment revealed that muscle mass measurement better reflects the risk of severe complications and postoperative pneumonia. Previous meta‐analyses have shown that low‐skeletal muscle mass increases the risk of severe complications or postoperative pneumonia [6, 7, 8]. Meanwhile, there was no difference in risk for long‐term survival or other postoperative complications even without muscle mass measurement. This may be the difference between outcomes that can be predicted based only on BMI and BWL rate, and outcomes that can be predicted more accurately by including muscle mass in addition to BMI and BWL rate.

The present study had several limitations. First, the overall strength of the conclusion was limited because the studies had a moderate or high risk of bias. It is possible that the effect size of the outcome may be overestimated or underestimated due to bias caused by inadequate adjustment for confounding factors in some studies. Therefore, further high‐quality prospective studies are warranted. Second, due to the small number of studies, subgroup analyses could not be adequately performed. In particular, the analysis by severity of malnutrition may have been insufficiently powered due to the limited number of studies. Further studies reporting outcomes by severity of malnutrition are needed to enable adequate consideration of effect size. Despite these limitations, this systematic review and meta‐analysis investigated the effect of GLIM‐defined malnutrition on short‐ and long‐term outcomes in patients with gastroenterological cancer who underwent surgery. Prospective or observational studies with statistical analyses are needed to eliminate confounding factors related to prognosis.

## 5 Conclusion

GLIM‐defined malnutrition may worsen the OS and increase the risk of postoperative complications in patients with gastroenterological cancer undergoing surgical treatment. Further studies are needed to confirm these findings and mitigate the risk of bias.

## Author Contributions

**Ryota Matsui:** conceptualization, investigation, writing – original draft, methodology, visualization, writing – review and editing, data curation, formal analysis, software. **Jun Watanabe:** conceptualization, investigation, writing – review and editing, validation, methodology, formal analysis, data curation. **Kazuma Rifu:** conceptualization, investigation, writing – review and editing, validation, methodology, data curation. **Souya Nunobe:** conceptualization, investigation, writing – review and editing, writing – original draft, methodology, validation, supervision. **Noriyuki Inaki:** conceptualization, investigation, writing – review and editing, validation, methodology, supervision.

## Funding

The authors have nothing to report.

## Ethics Statement

The authors have nothing to report.

## Consent

The authors have nothing to report.

## Conflicts of Interest

The authors declare no conflicts of interest.

## Supporting information


**Figure S1:** Prevalence of GLIM‐defined malnutrition.
**Figure S2:** Subgroup analysis or sensitivity analysis of overall survival.
**Figure S3:** Subgroup analysis or sensitivity analysis of relapse‐free survival.
**Figure S4:** Subgroup analysis or sensitivity analysis of total postoperative complications.
**Figure S5:** Subgroup analysis or sensitivity analysis of severe complications.
**Figure S6:** Subgroup analysis or sensitivity analysis of infectious complications.
**Figure S7:** Subgroup analysis or sensitivity analysis of anastomotic leakage.
**Figure S8:** Subgroup analysis or sensitivity analysis of postoperative pneumonia.
**Figure S9:** Subgroup analysis or sensitivity analysis of mortality.
**Figure S10:** Subgroup analysis or sensitivity analysis of postoperative hospital stay.
**Figure S11:** Funnel plots.

**Appendix S1:** PRISMA 2020 checklist.

**Appendix S2:** The electronic database search strategy.
**Appendix S3:** The trial registry search strategy.
**Appendix S4:** Characteristics of studies excluded from the qualitative and quantitative synthesis.
**Appendix S5:** Risk of bias for eligible studies using the Quality In Prognosis Studies tool.

## Data Availability

The datasets generated and/or analyzed in the current study are available from the corresponding author upon reasonable request.
