# Supplementary material for: Relationship Between GLIM‐Defined Malnutrition and Postoperative Outcomes After Curative Resection in Patients With Gastroenterological Cancer: Update Systematic Review and Meta‐Analysis
Source: Ann Gastroenterol Surg. 2026 Jan 27;10(2):348–62. doi: 10.1002/ags3.70173 (PMC12962040; doi:10.1002/ags3.70173)
Supplement: Supplementary file 1 — Figure S1: Prevalence of GLIM‐defined malnutrition. Figure S2: Subgroup analysis or sensitivity analysis of overall survival. Figure S3: Subgroup analysis or sensitivity analysis of relapse‐free survival. Figure S4: Subgroup analysis or sensitivity analysis of total postoperative complications. Figure S5: Subgroup analysis or sensitivity analysis of severe complications. Figure S6: Subgroup analysis or sensitivity analysis of infectious complications. Figure S7: Subgroup analysis or sensitivity analysis of anastomotic leakage. Figure S8: Subgroup analysis or sensitivity analysis of postoperative pneumonia. Figure S9: Subgroup analysis or sensitivity analysis of mortality. Figure S10: Subgroup analysis or sensitivity analysis of postoperative hospital stay. Figure S11: Funnel plots. [file AGS3-10-348-s001.pptx]

## Slide 1
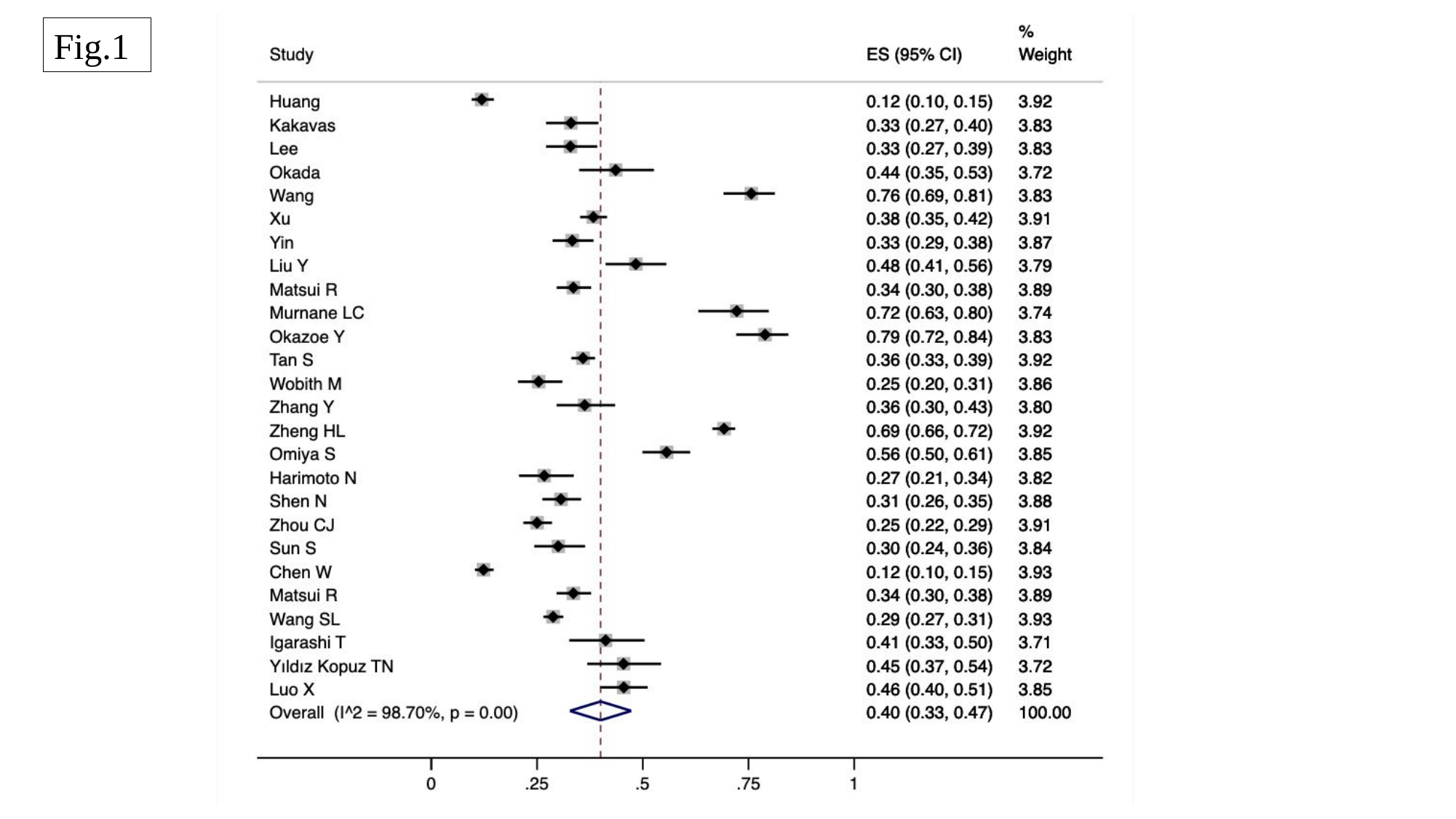

Fig.1

## Slide 2
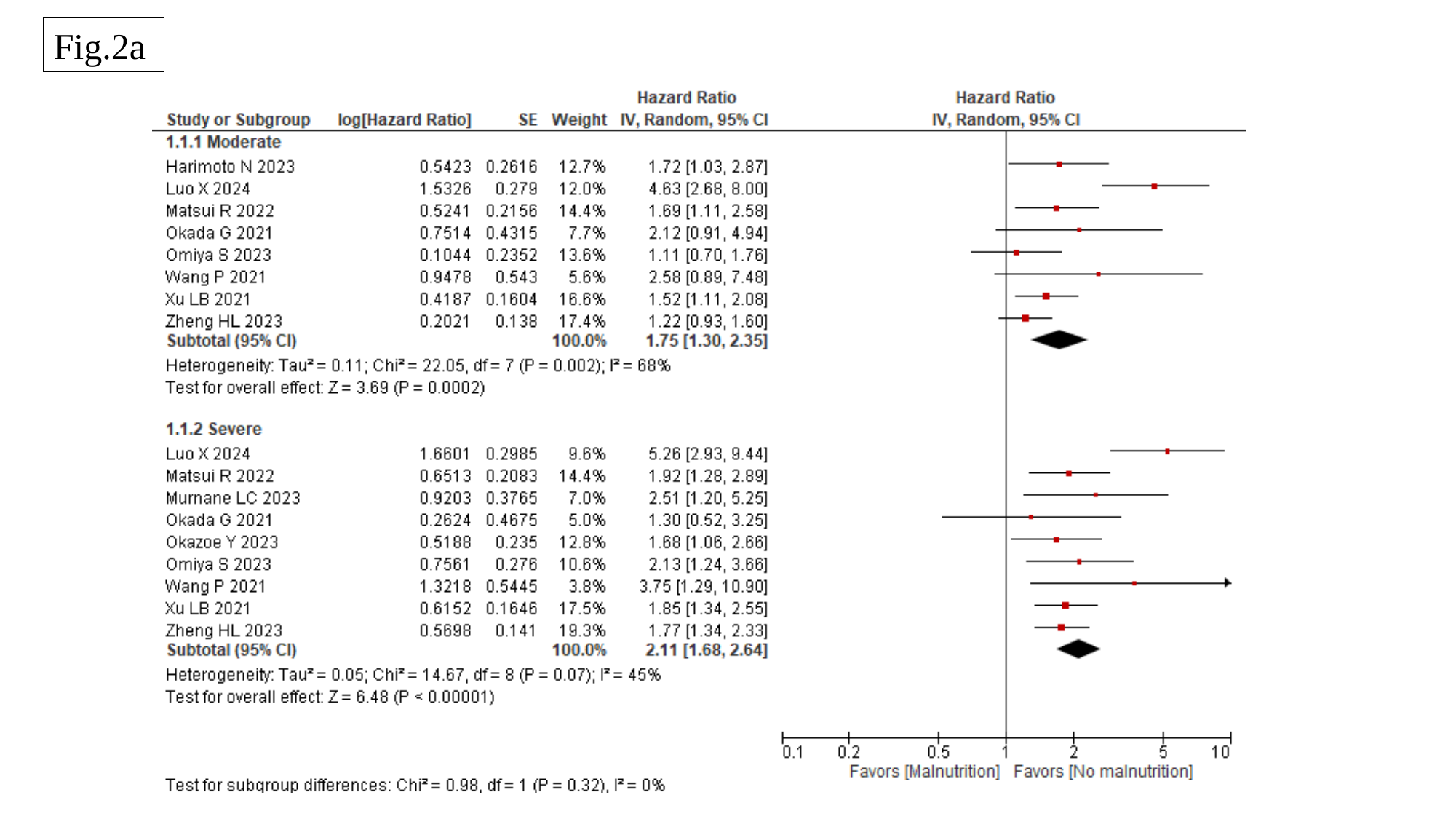

Fig.2a

## Slide 3
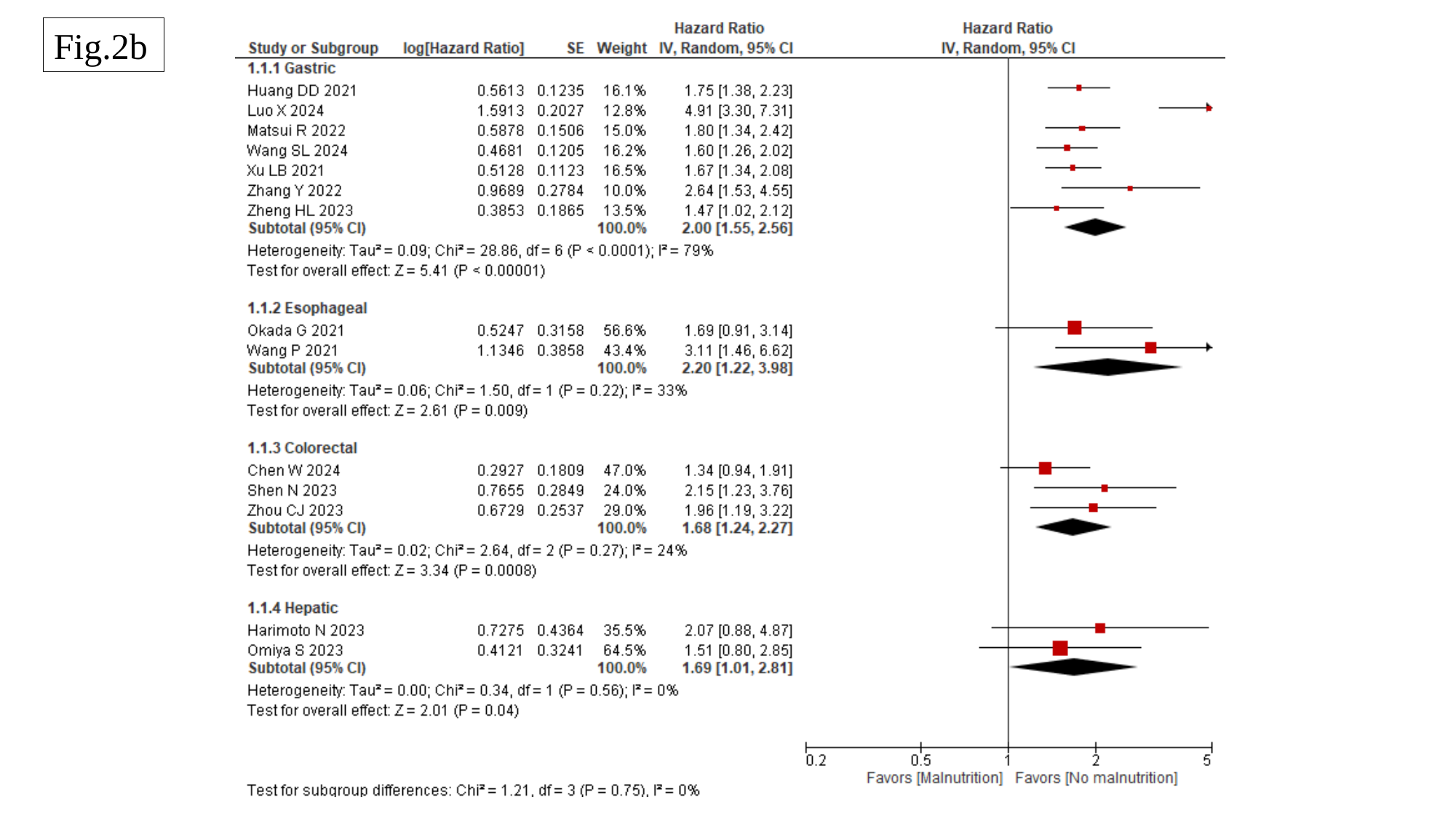

Fig.2b

## Slide 4
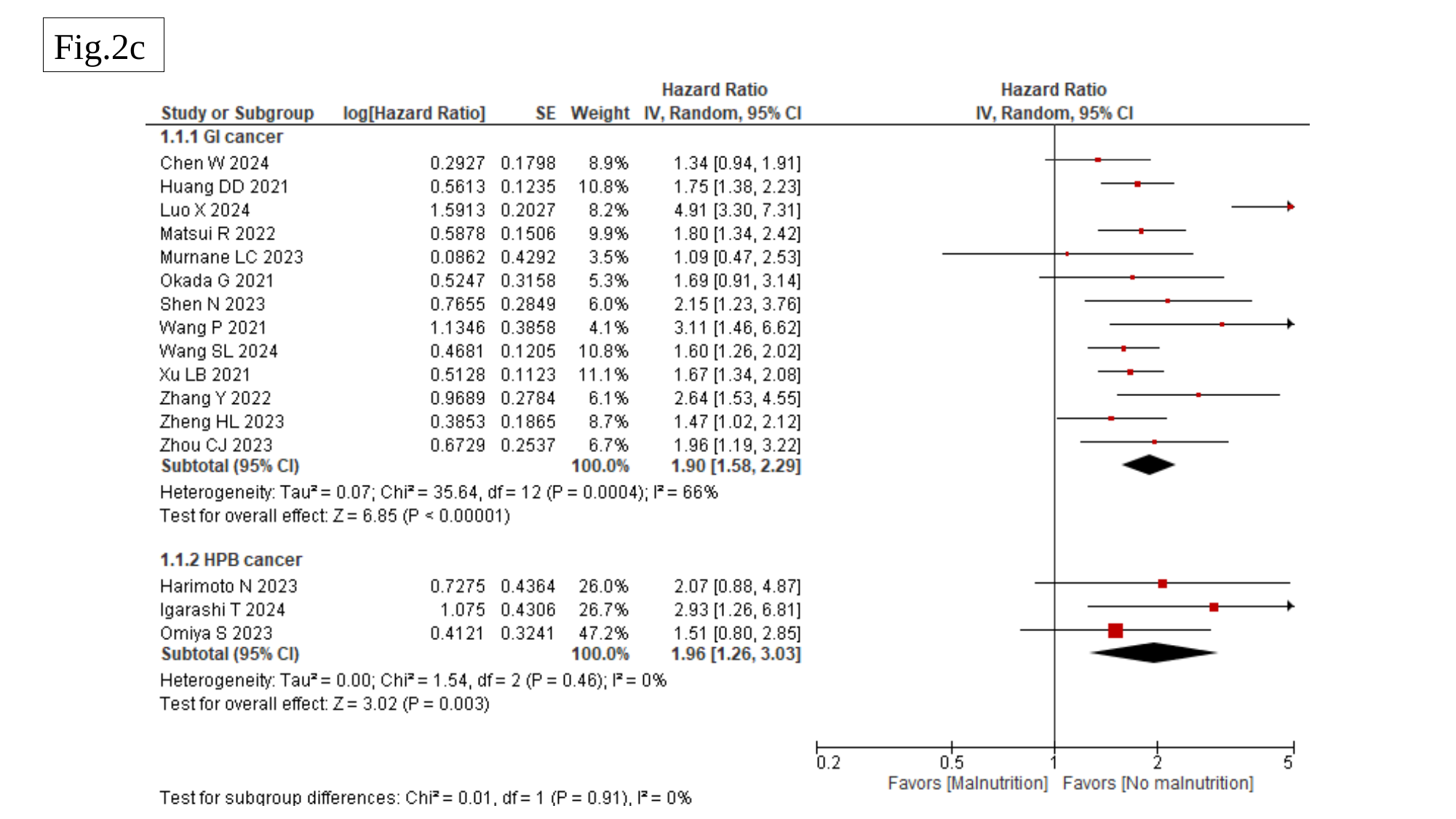

Fig.2c

## Slide 5
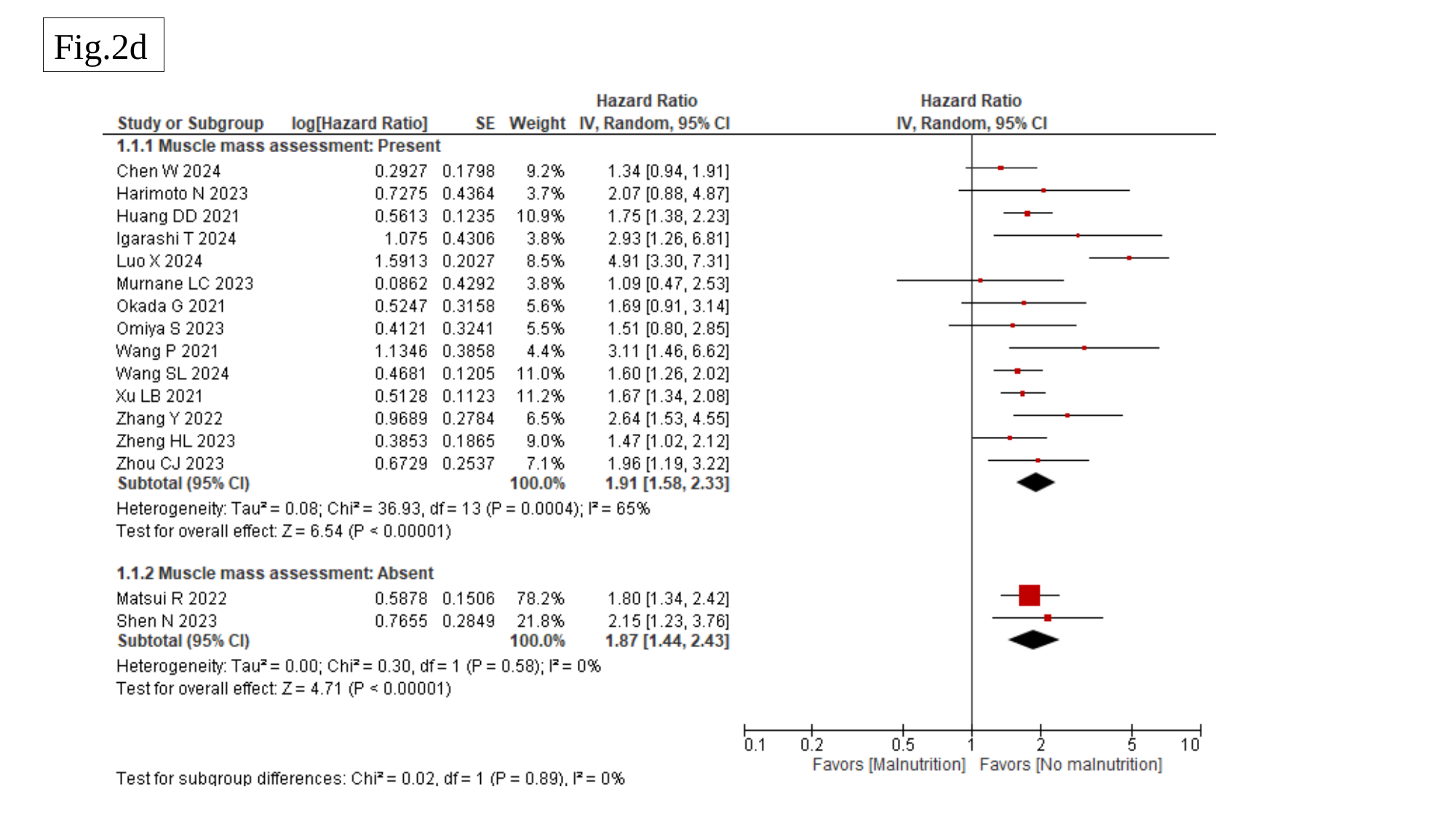

Fig.2d

## Slide 6
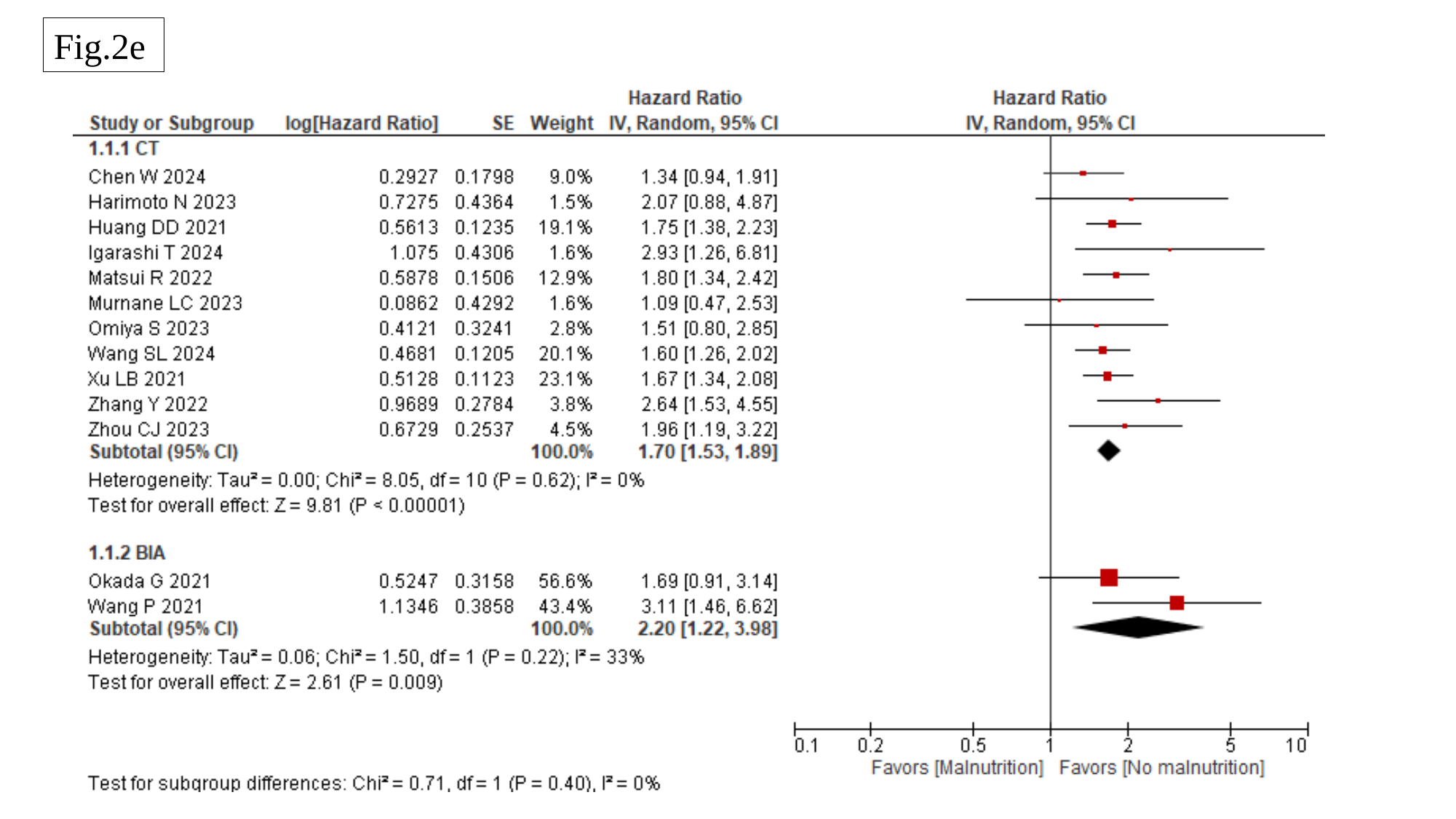

Fig.2e

## Slide 7
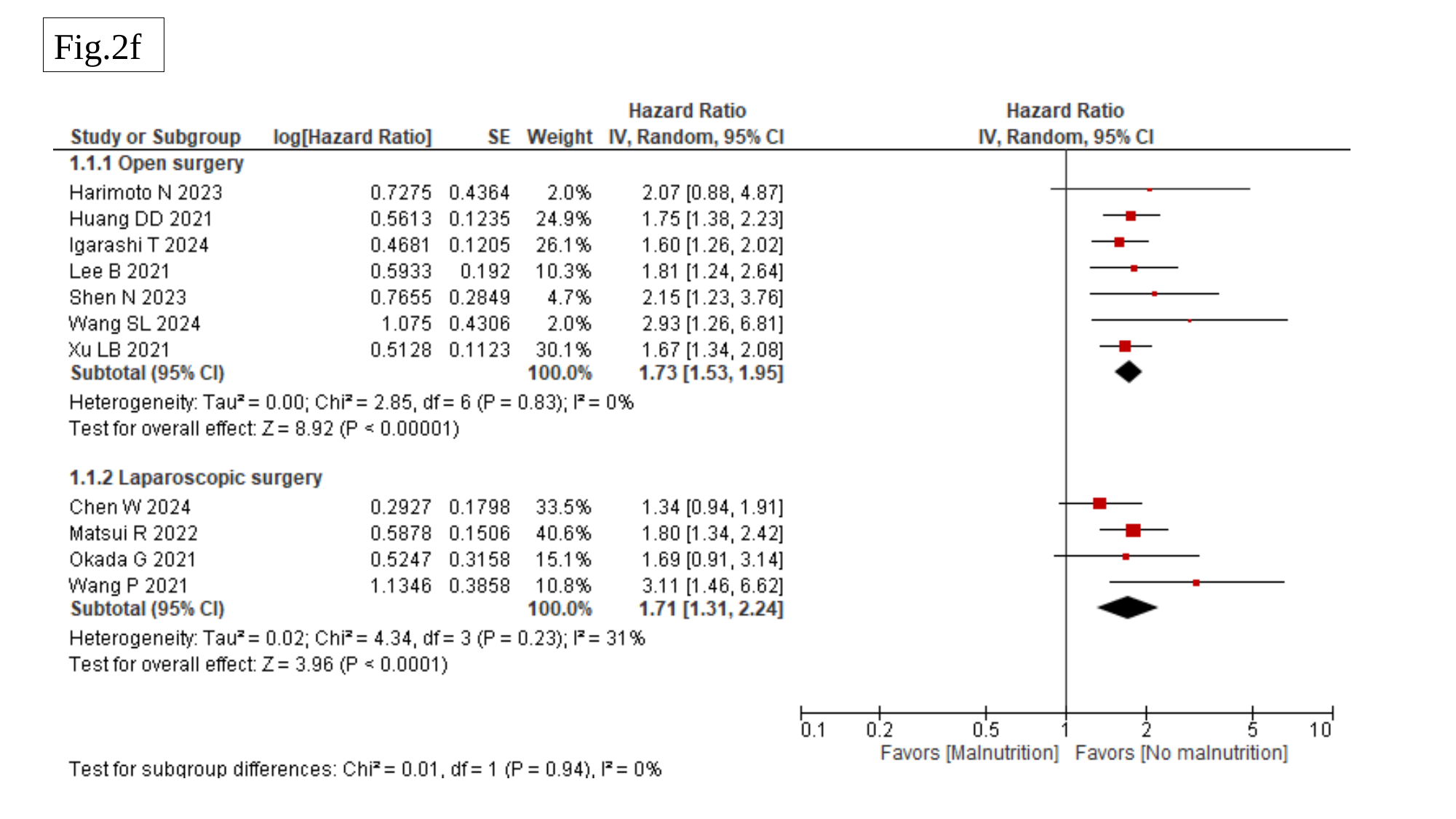

Fig.2f

## Slide 8
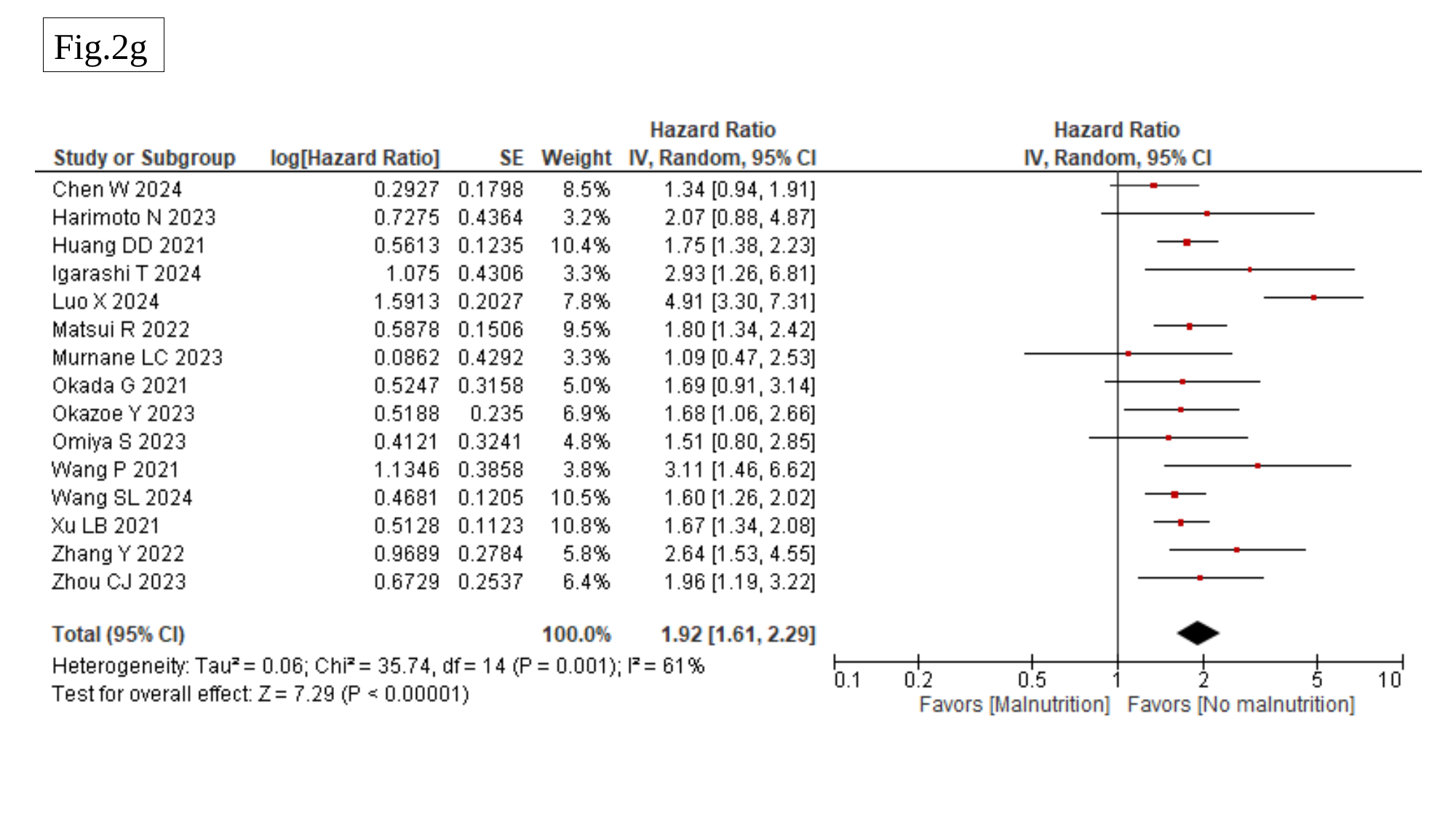

Fig.2g

## Slide 9
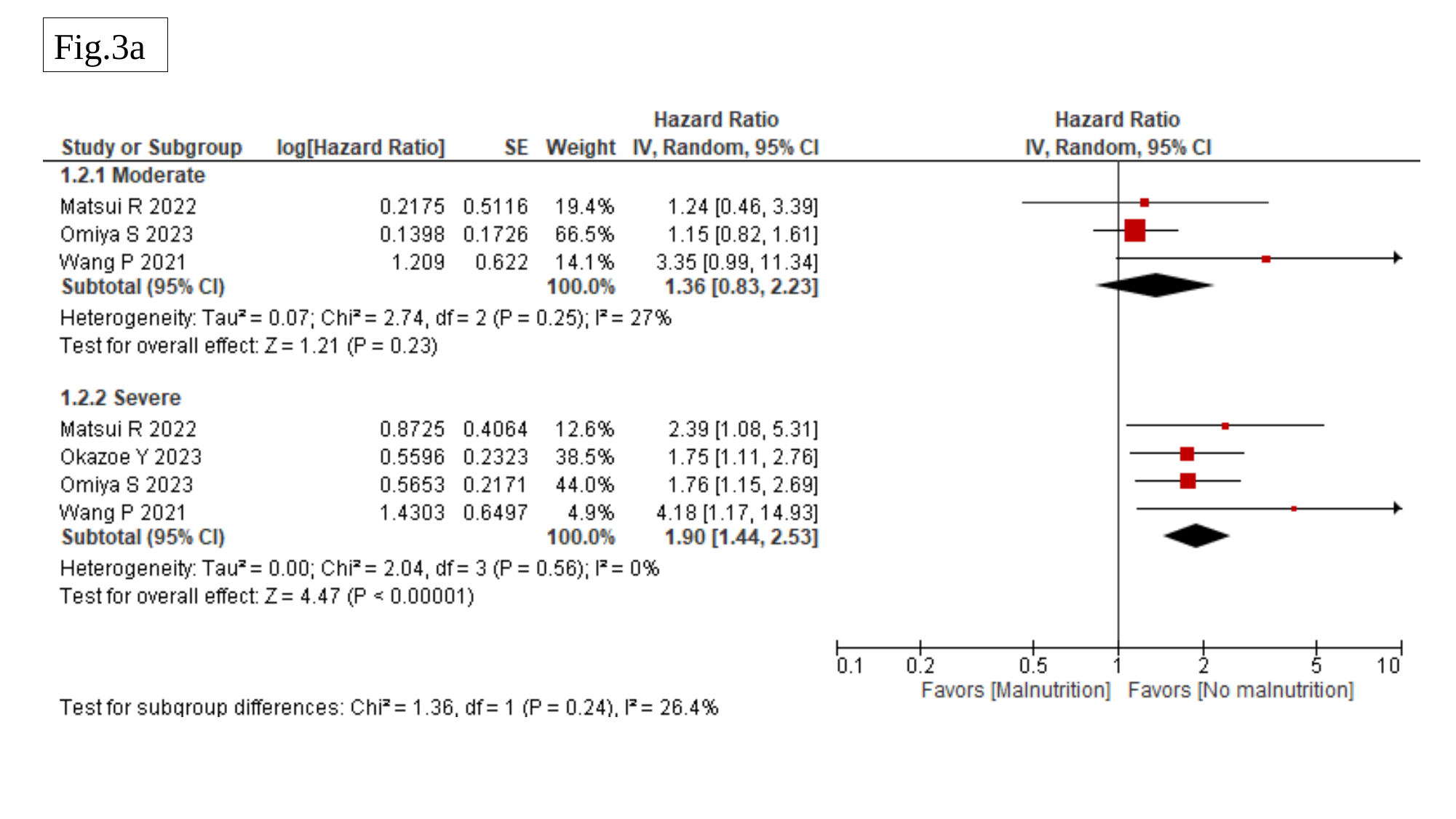

Fig.3a

## Slide 10
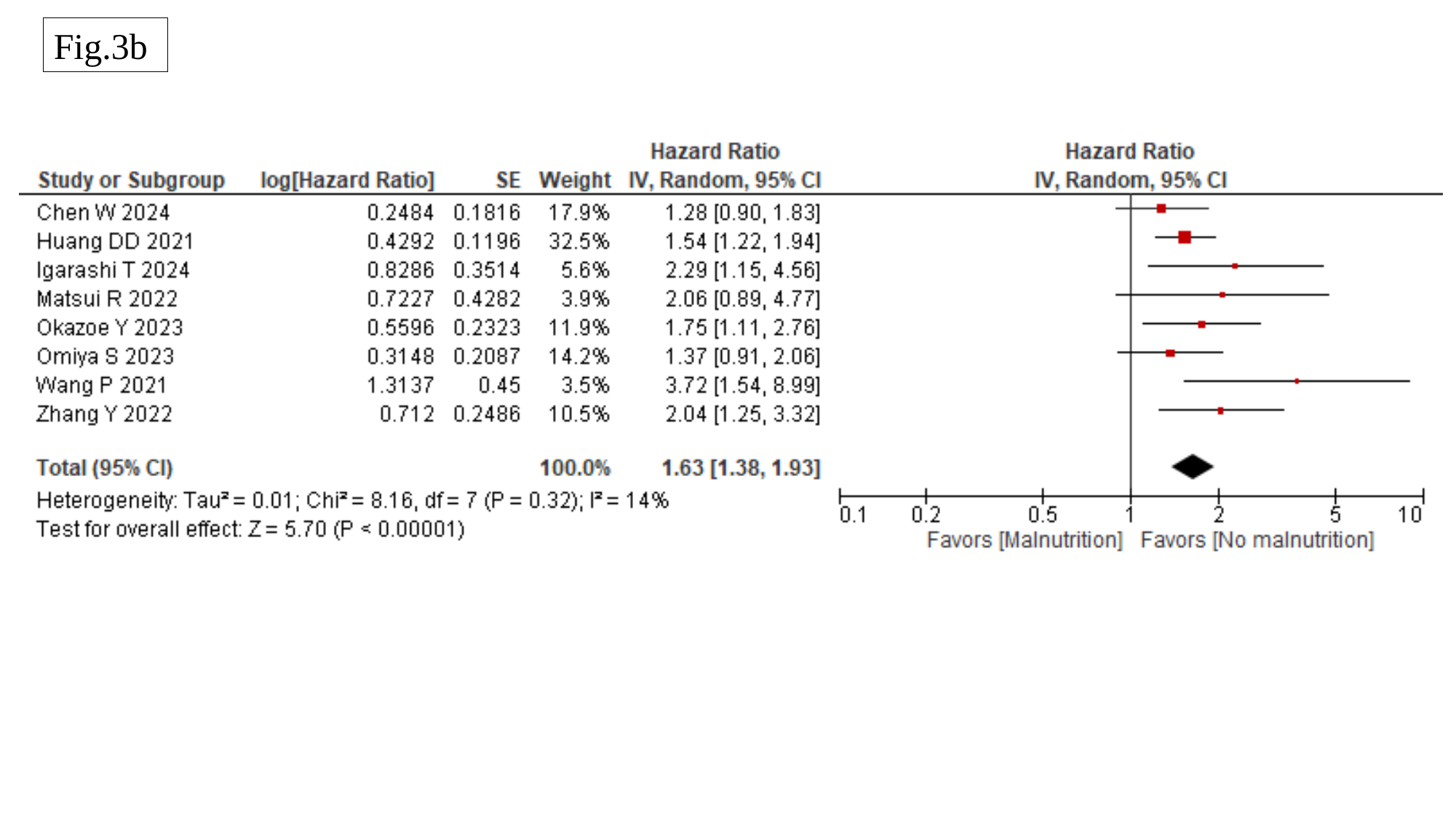

Fig.3b

## Slide 11
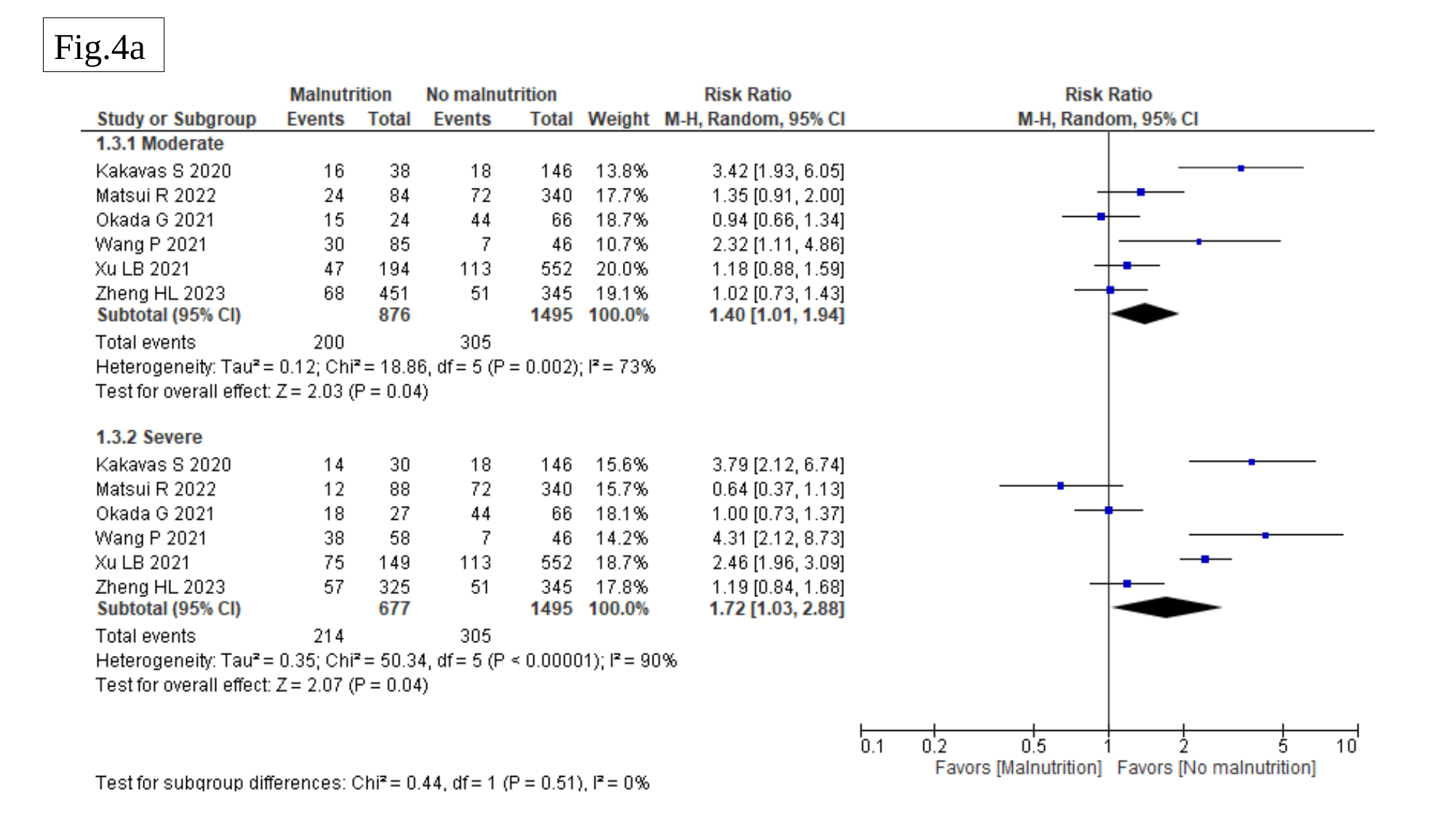

Fig.4a

## Slide 12
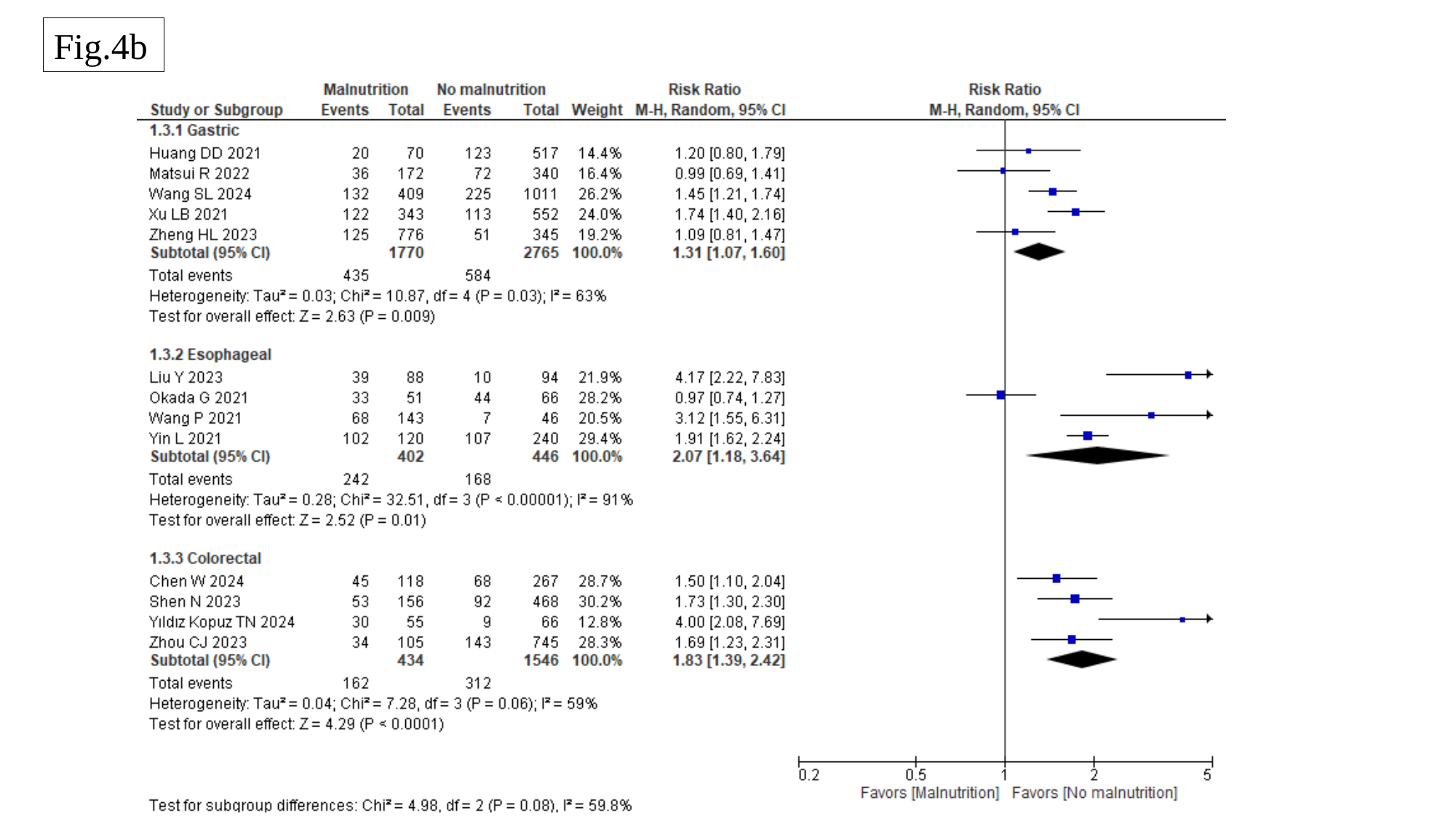

Fig.4b

## Slide 13
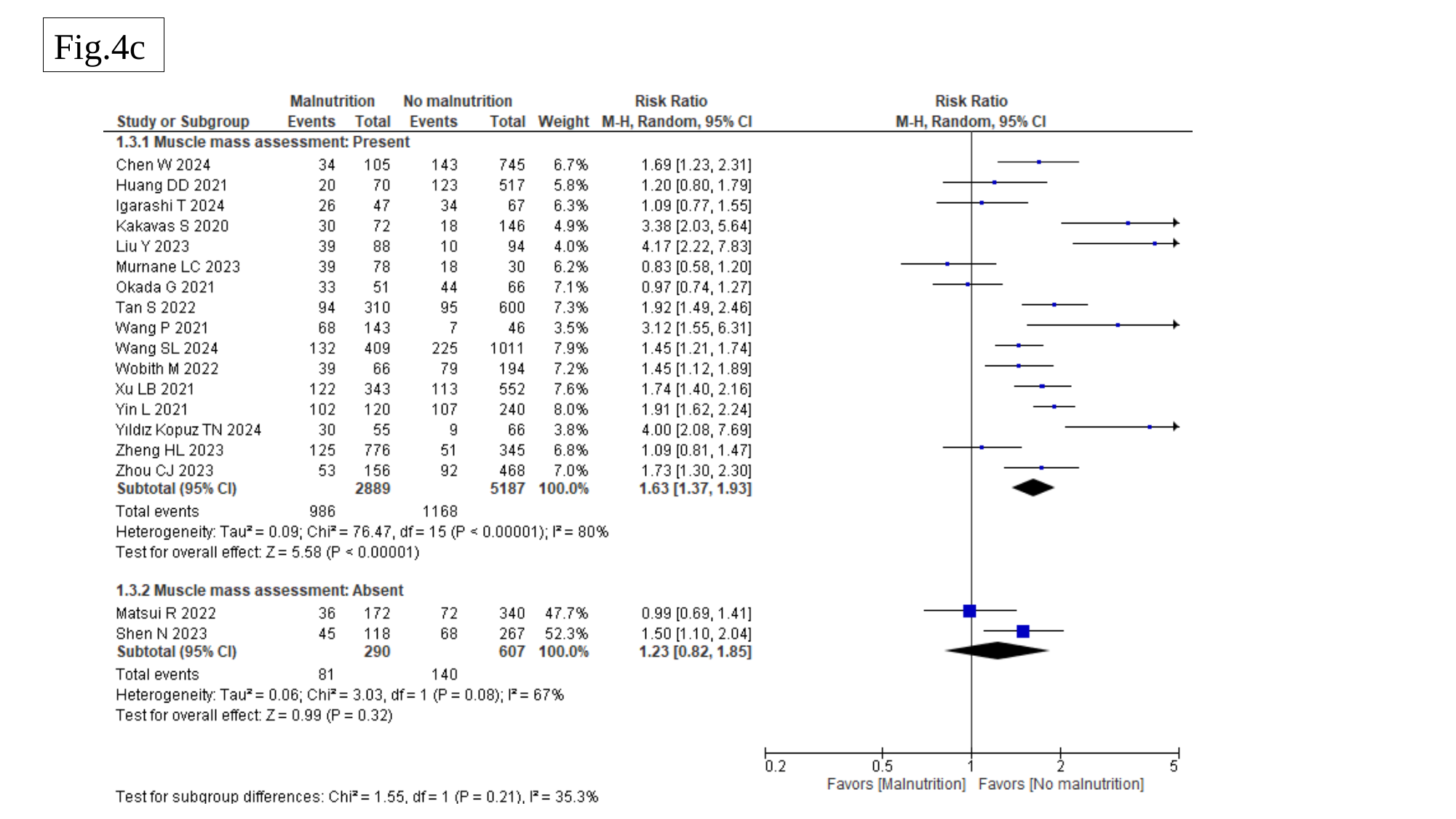

Fig.4c

## Slide 14
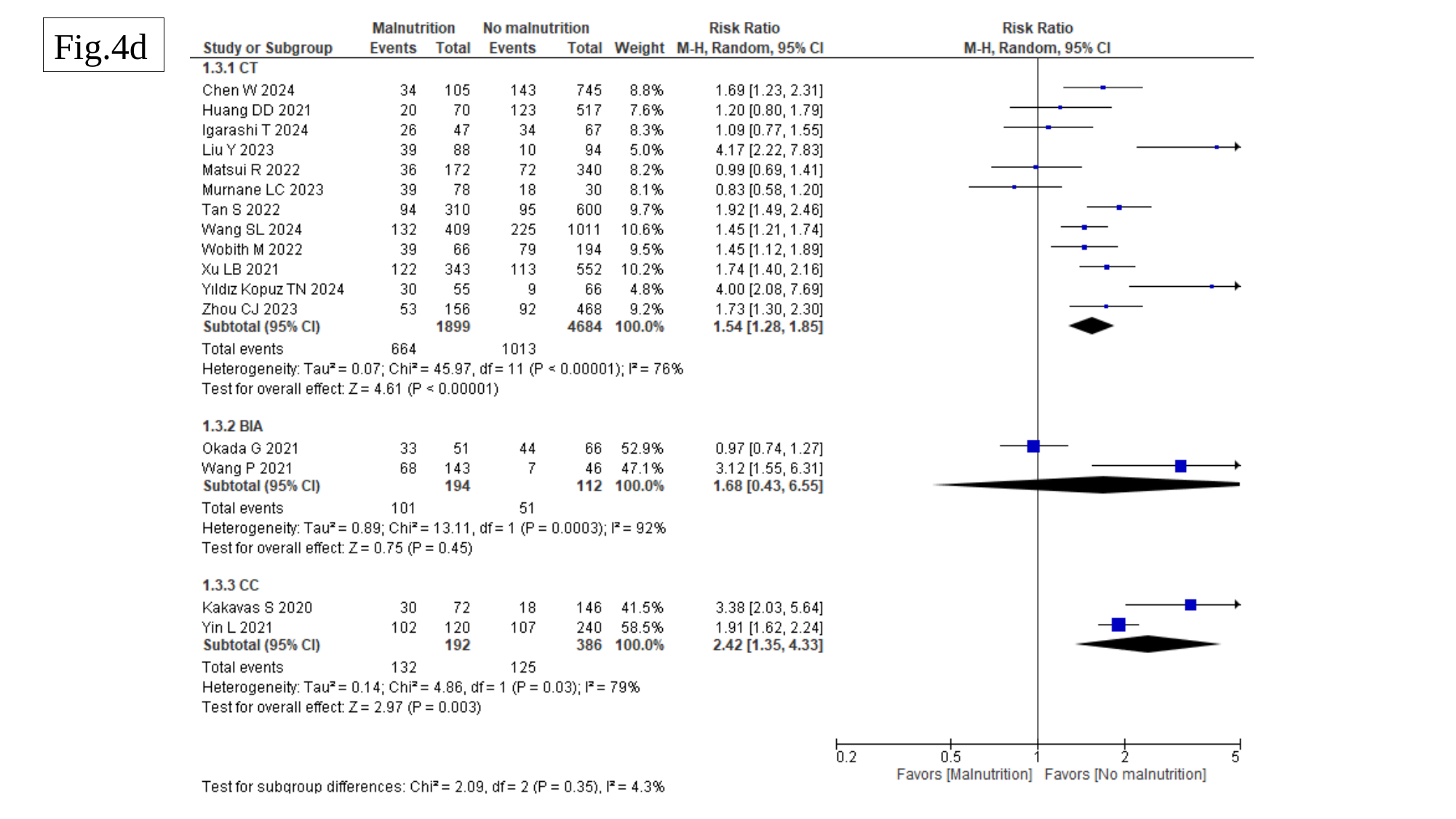

Fig.4d

## Slide 15
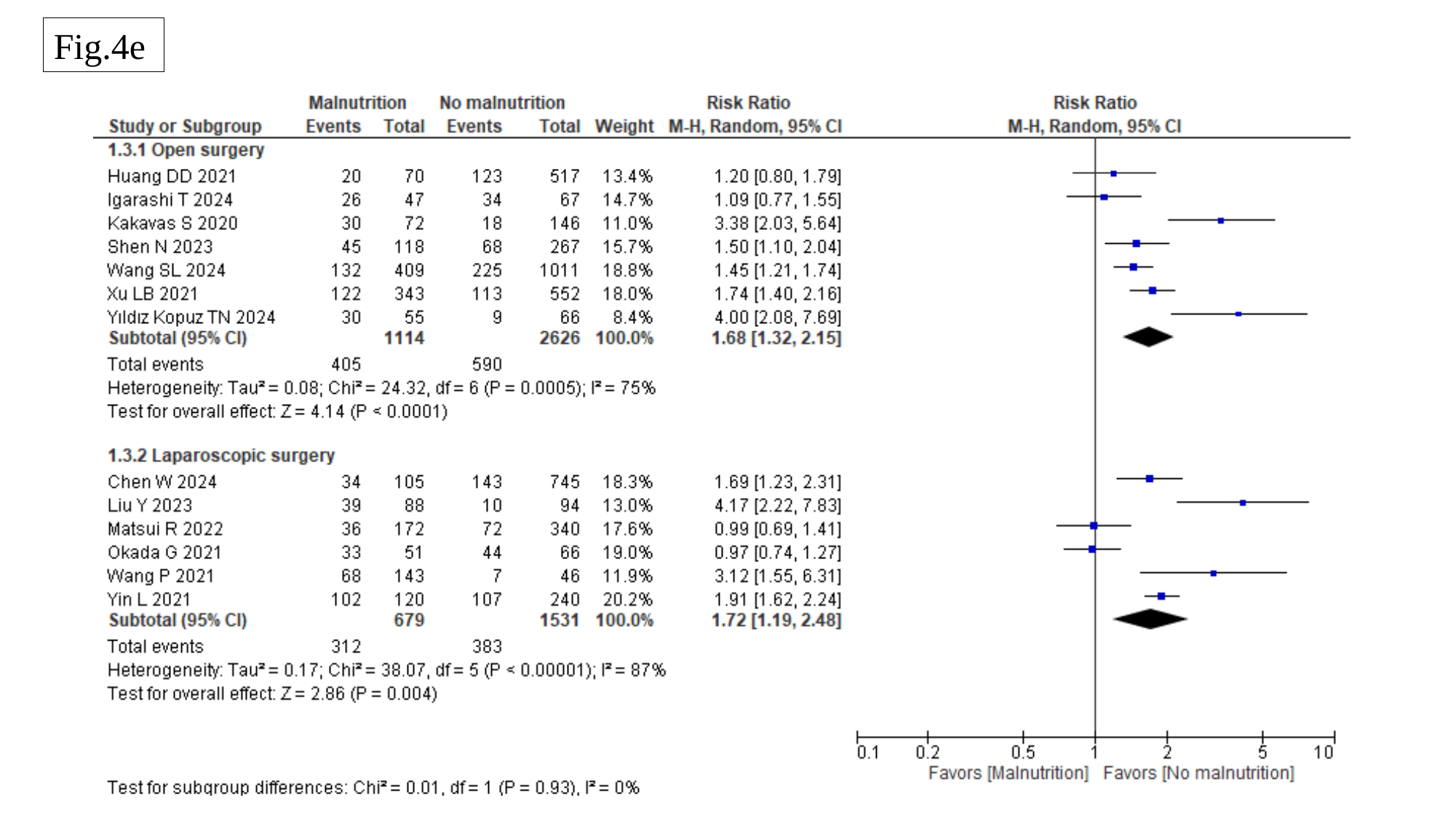

Fig.4e

## Slide 16
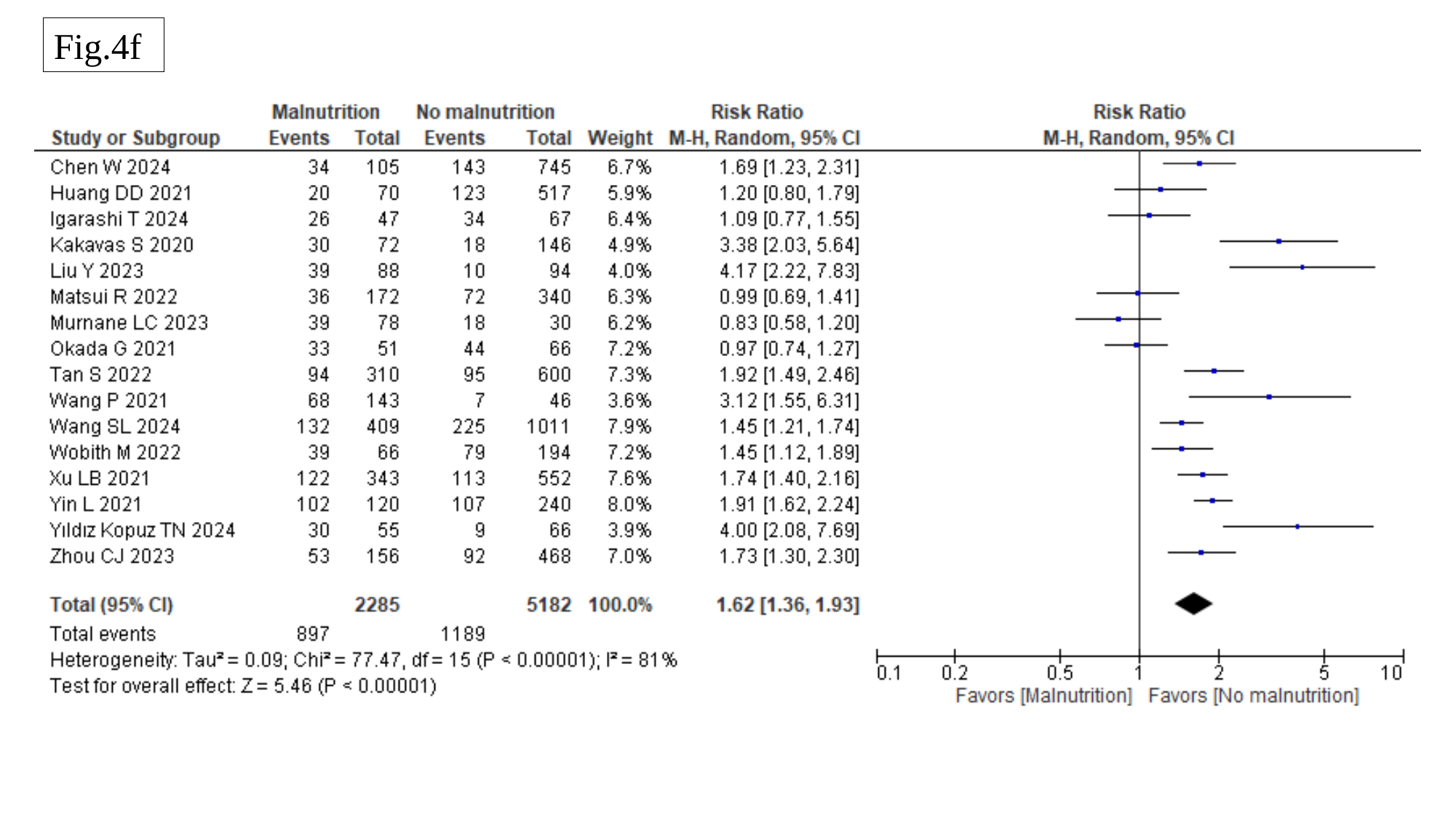

Fig.4f

## Slide 17
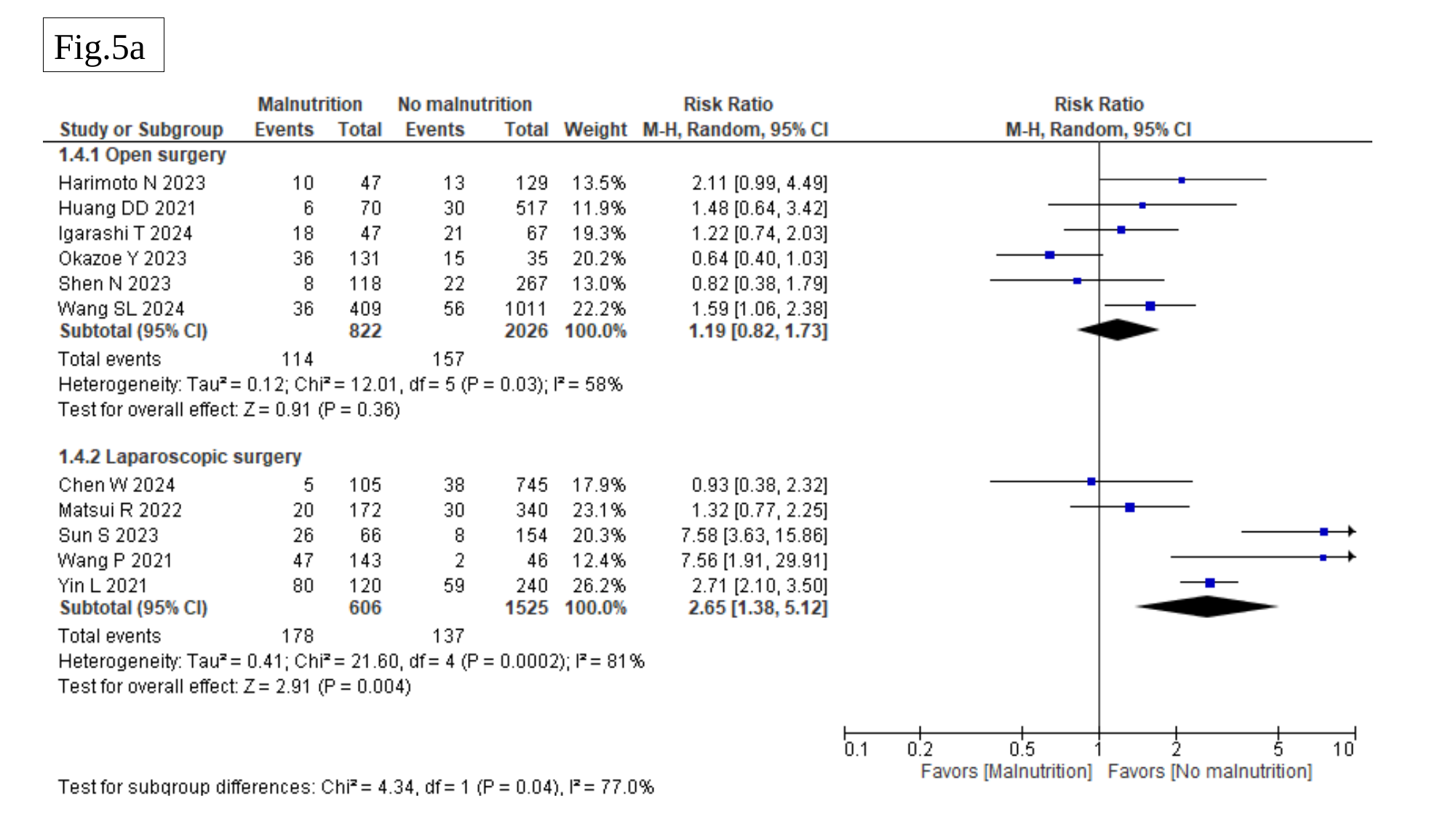

Fig.5a

## Slide 18
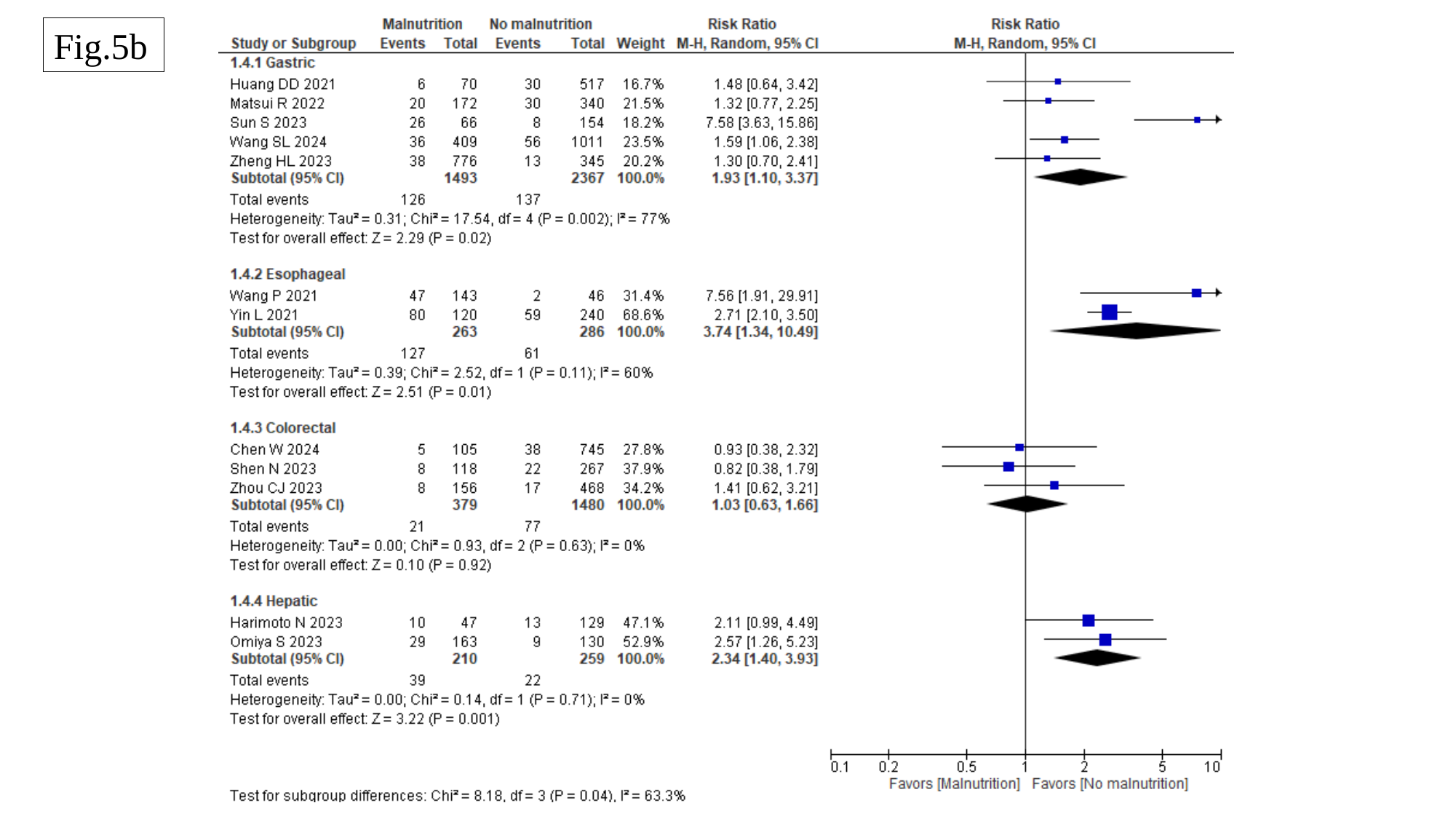

Fig.5b

## Slide 19
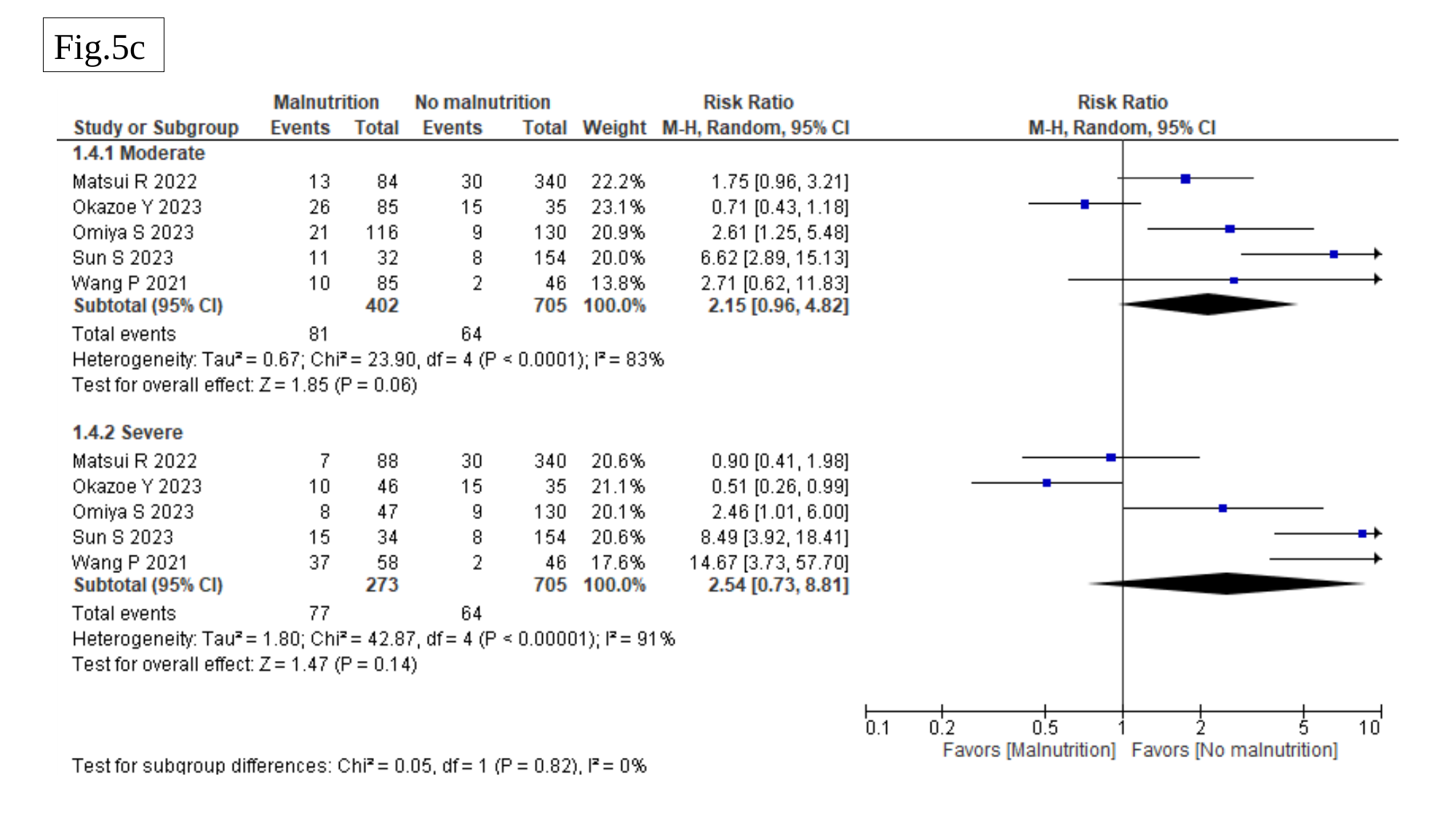

Fig.5c

## Slide 20
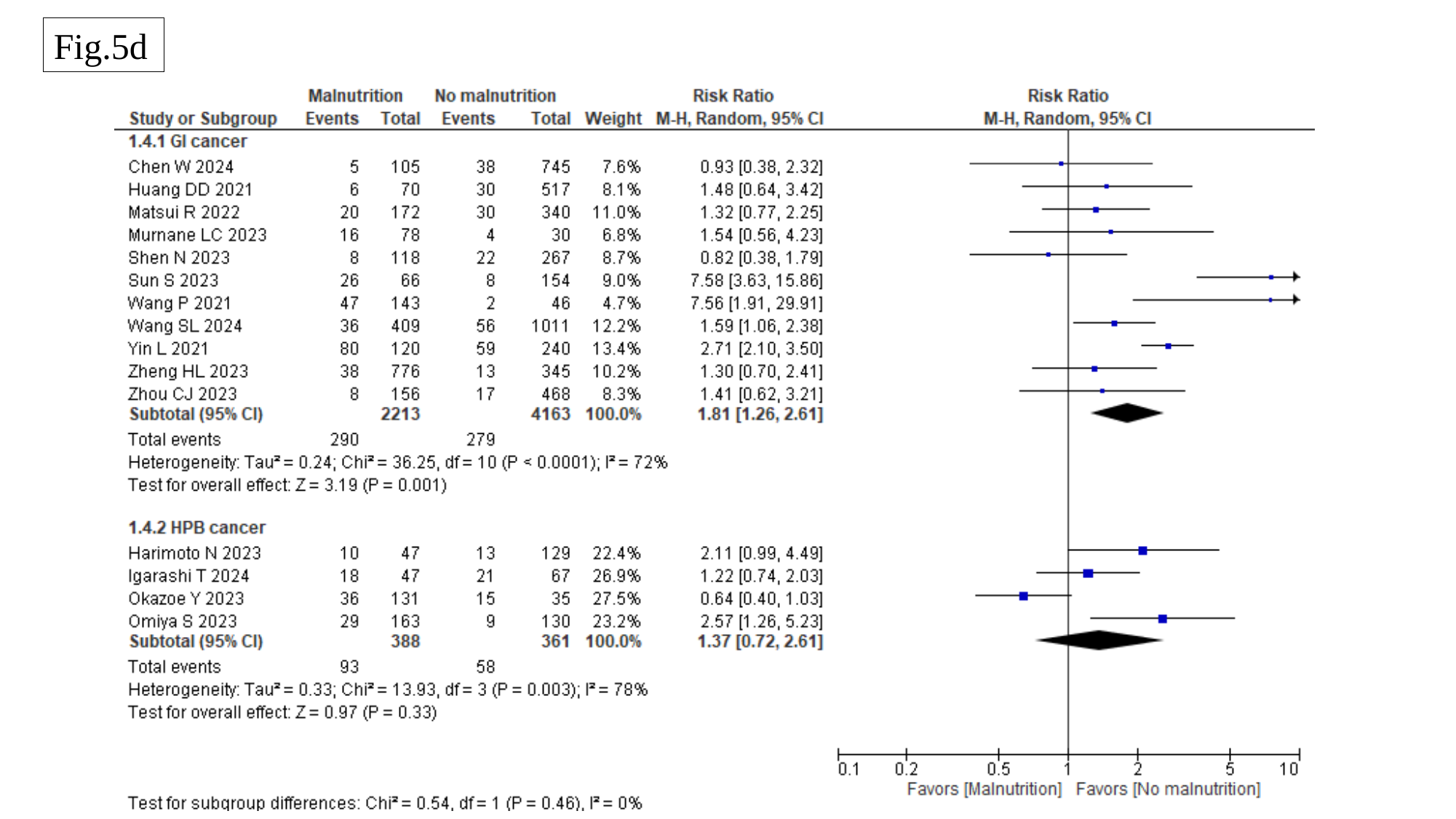

Fig.5d

## Slide 21
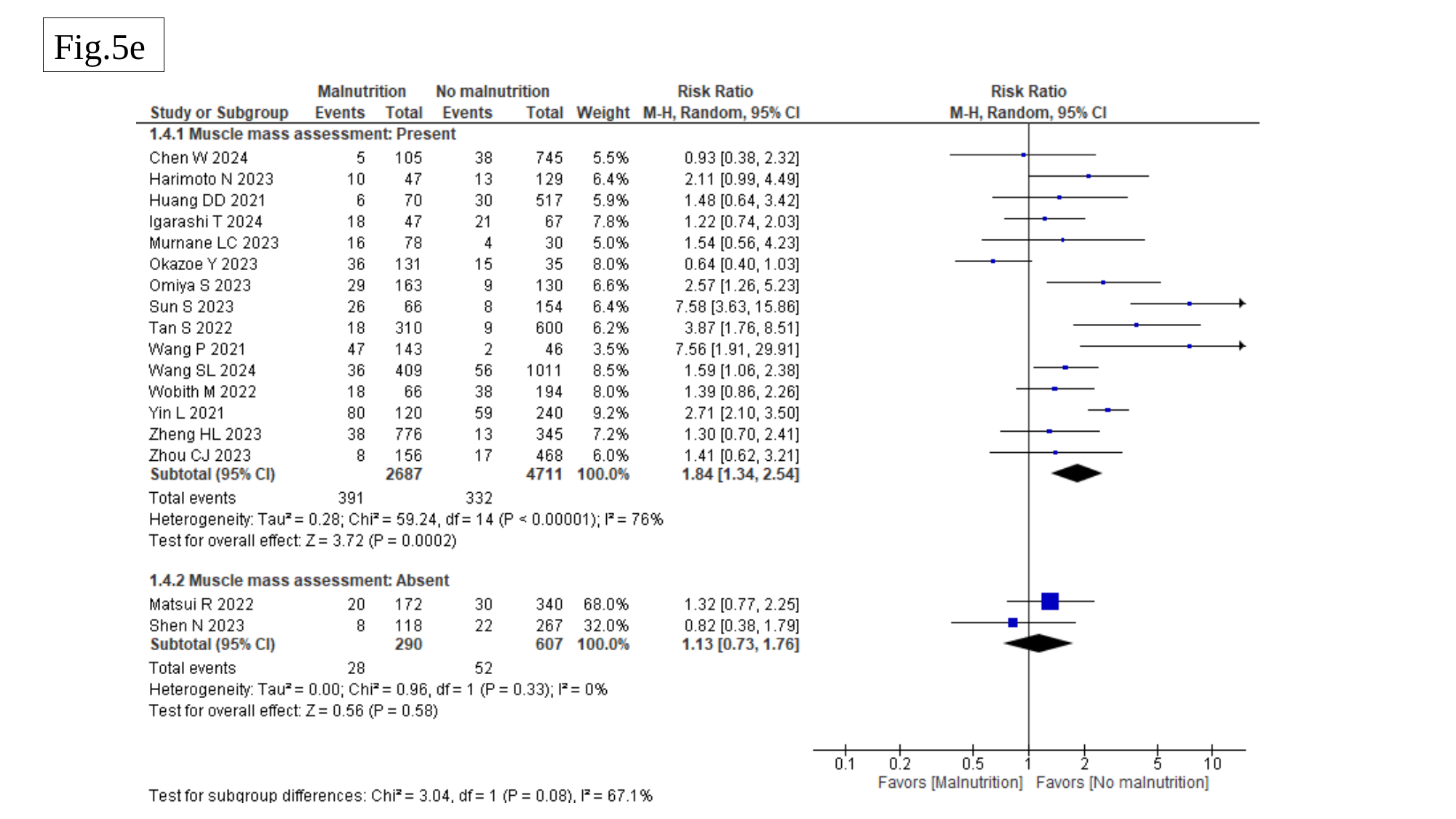

Fig.5e

## Slide 22
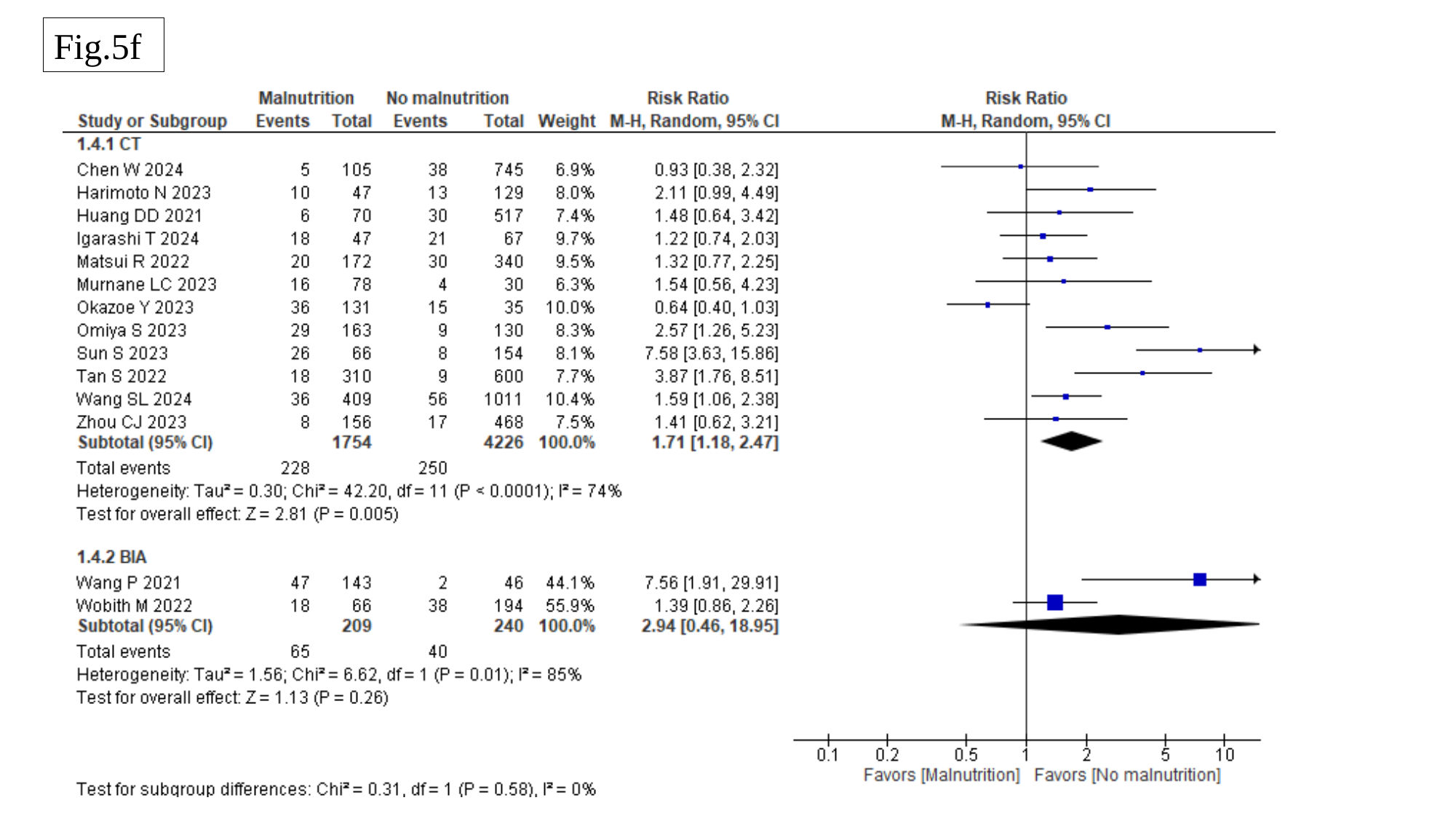

Fig.5f

## Slide 23
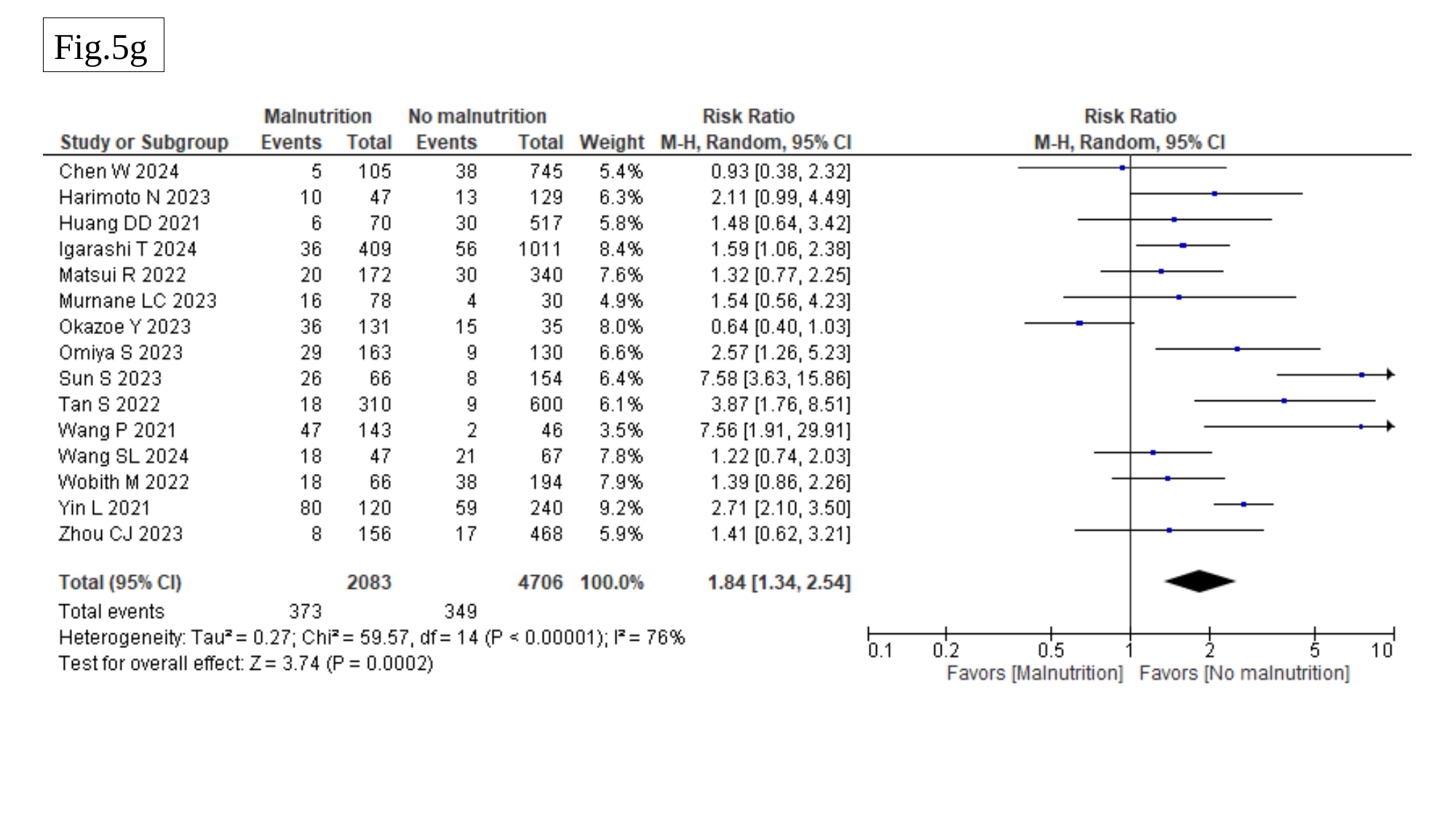

Fig.5g

## Slide 24
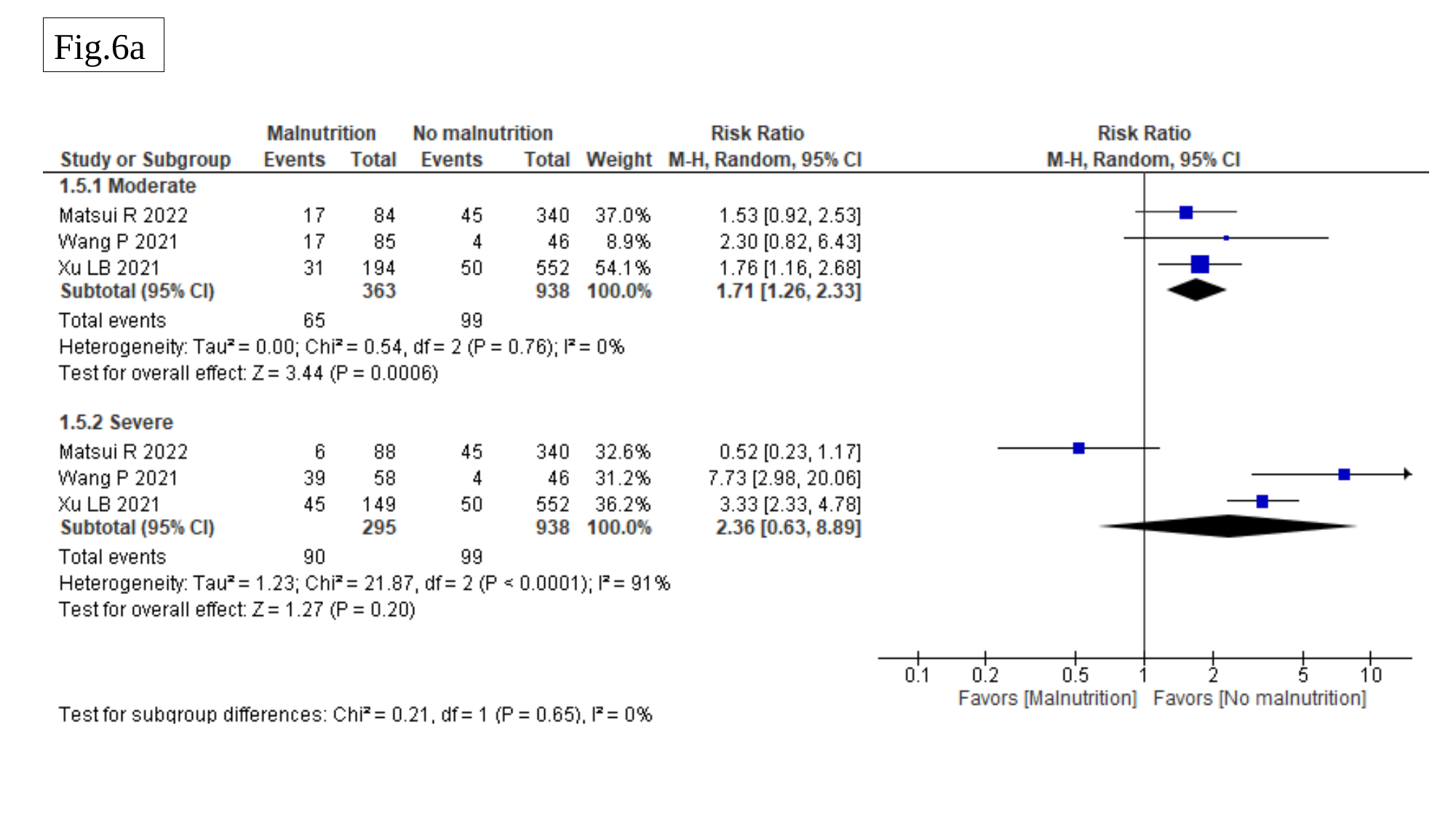

Fig.6a

## Slide 25
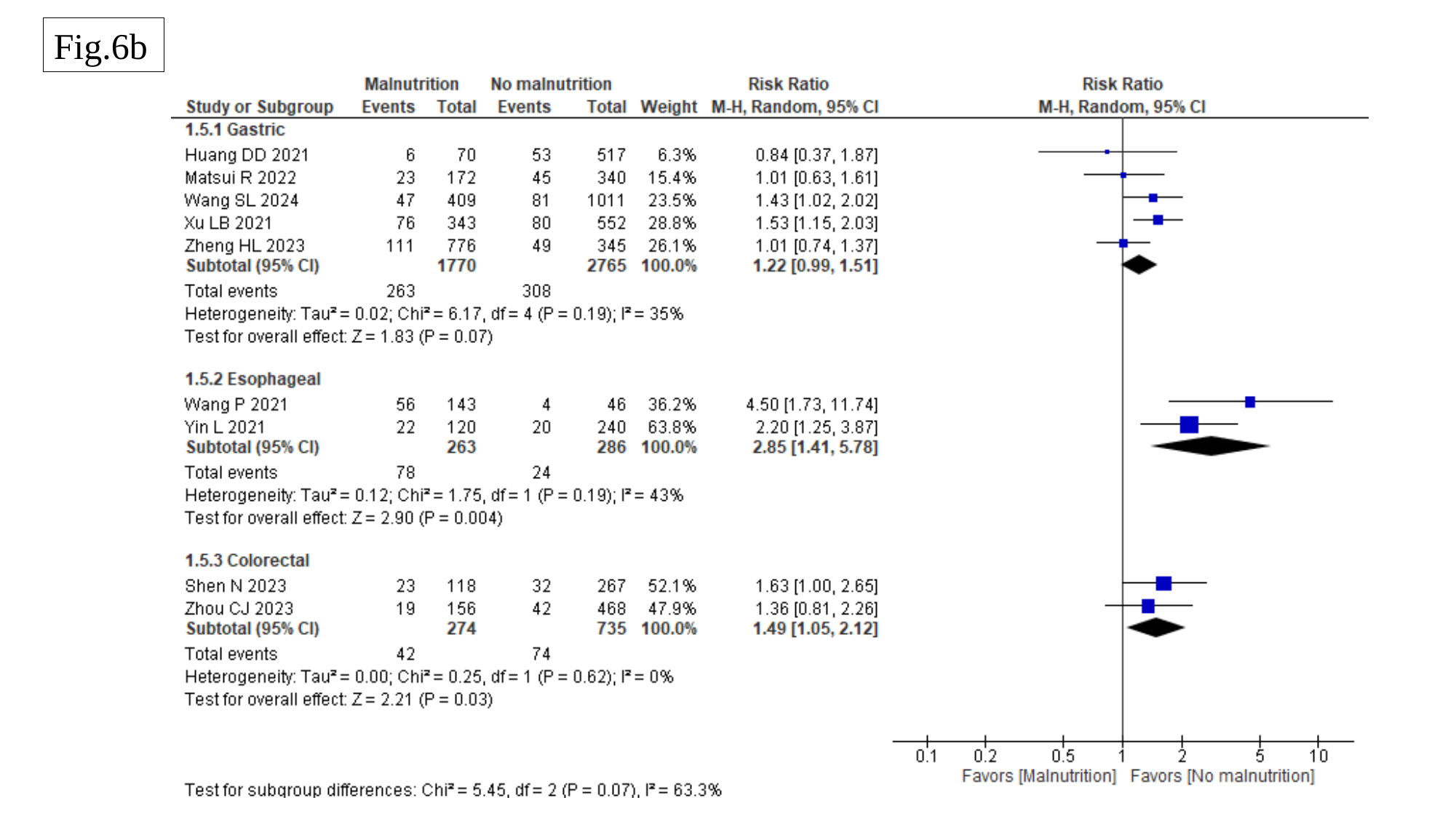

Fig.6b

## Slide 26
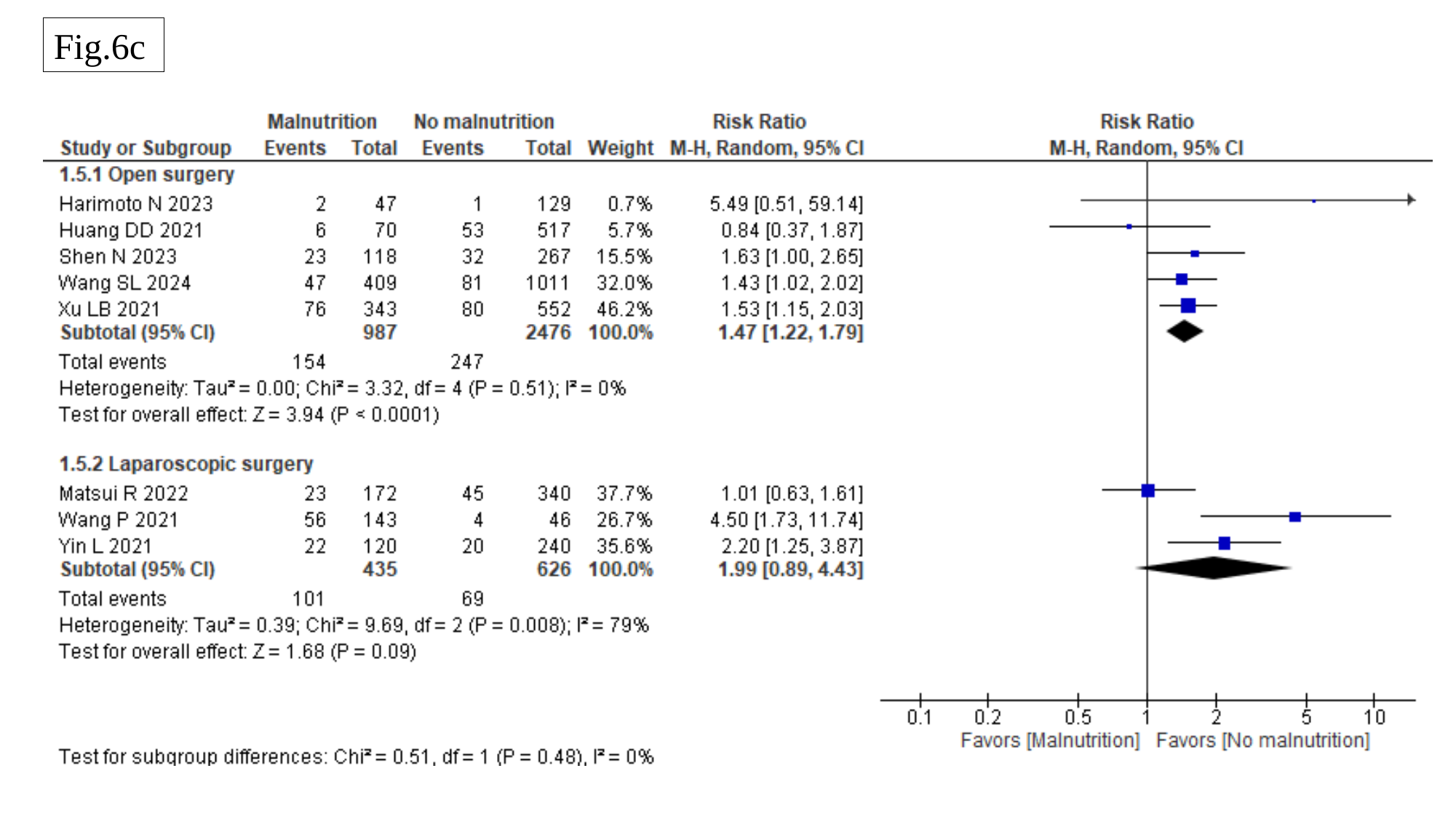

Fig.6c

## Slide 27
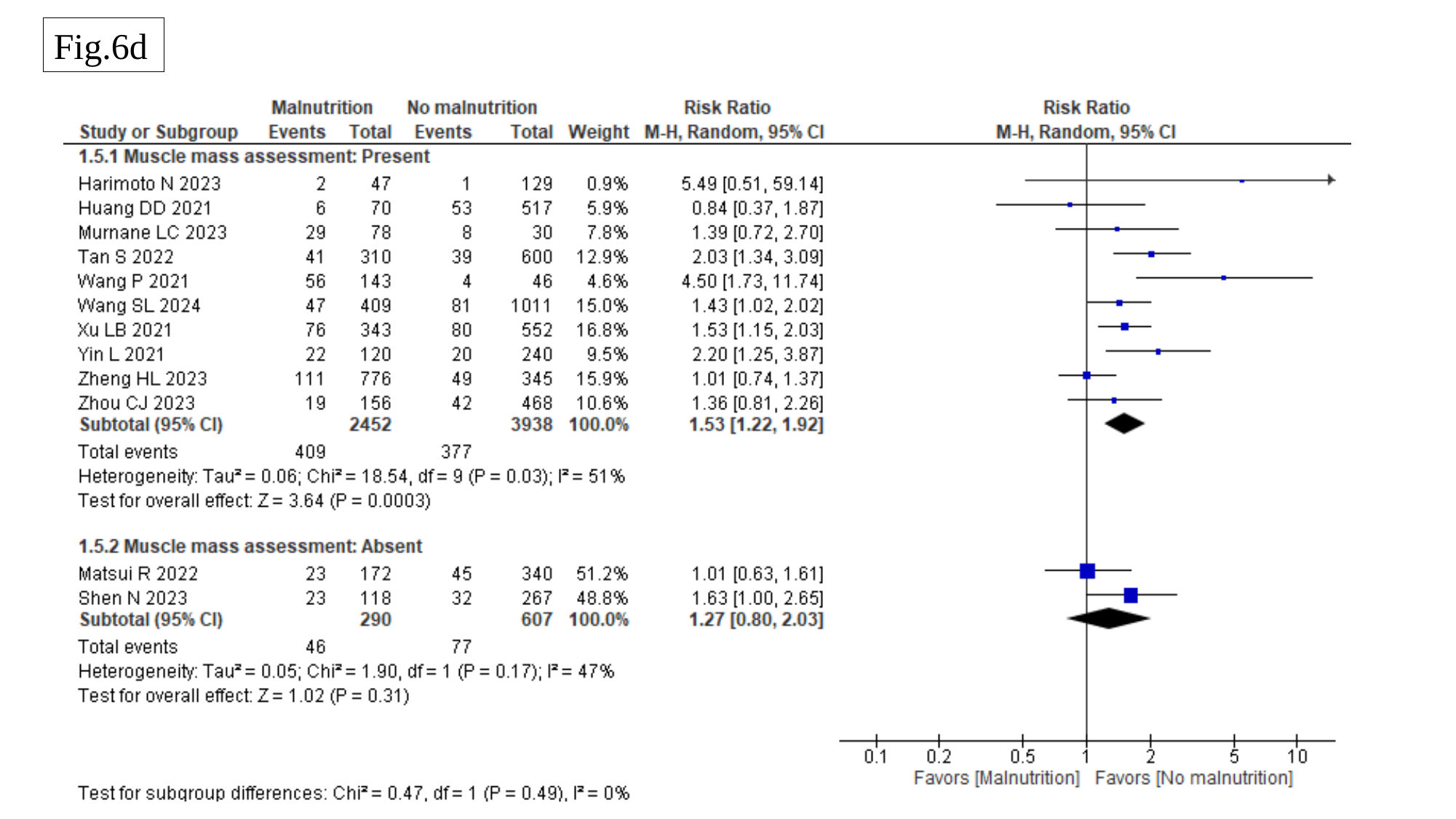

Fig.6d

## Slide 28
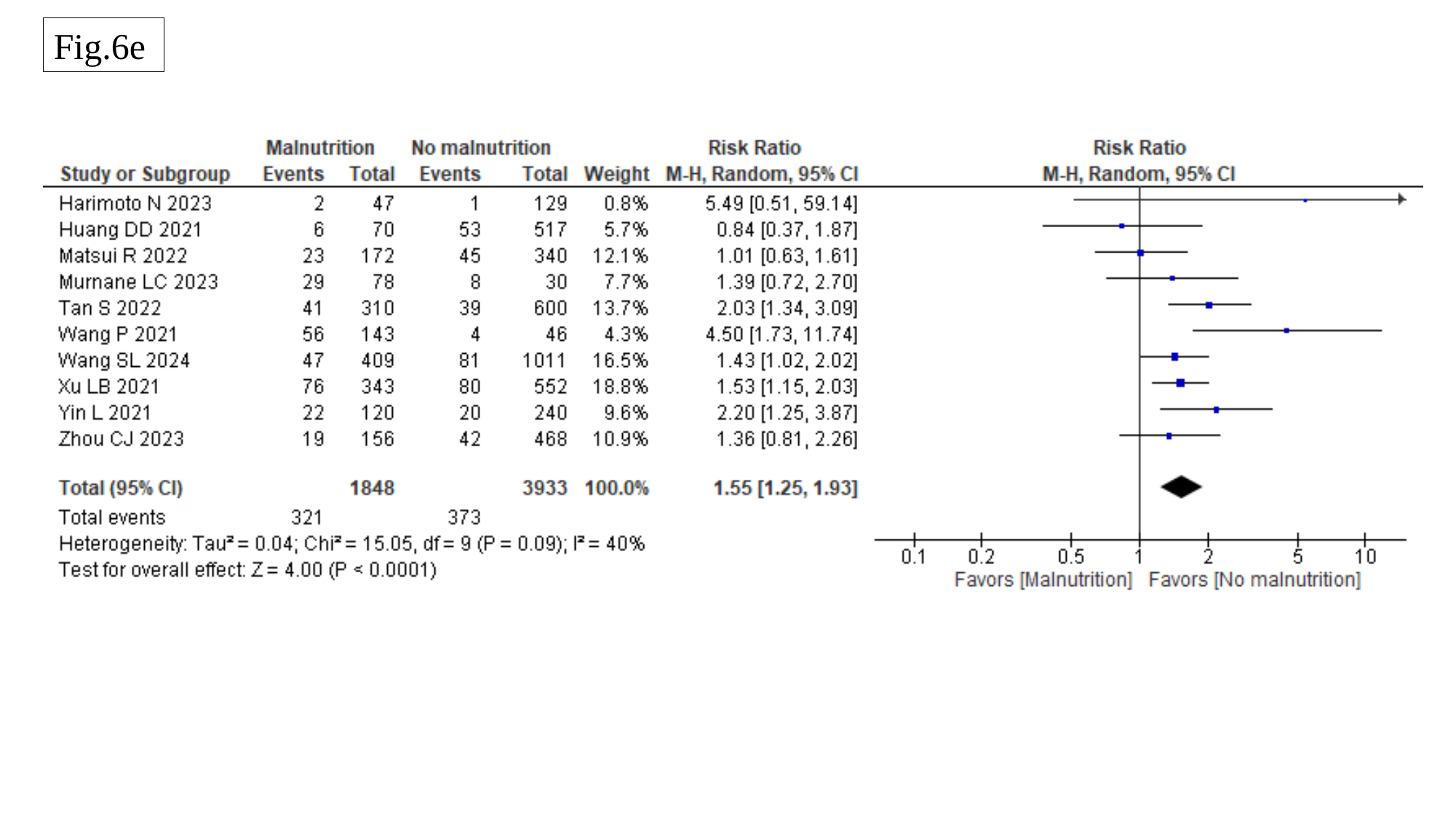

Fig.6e

## Slide 29
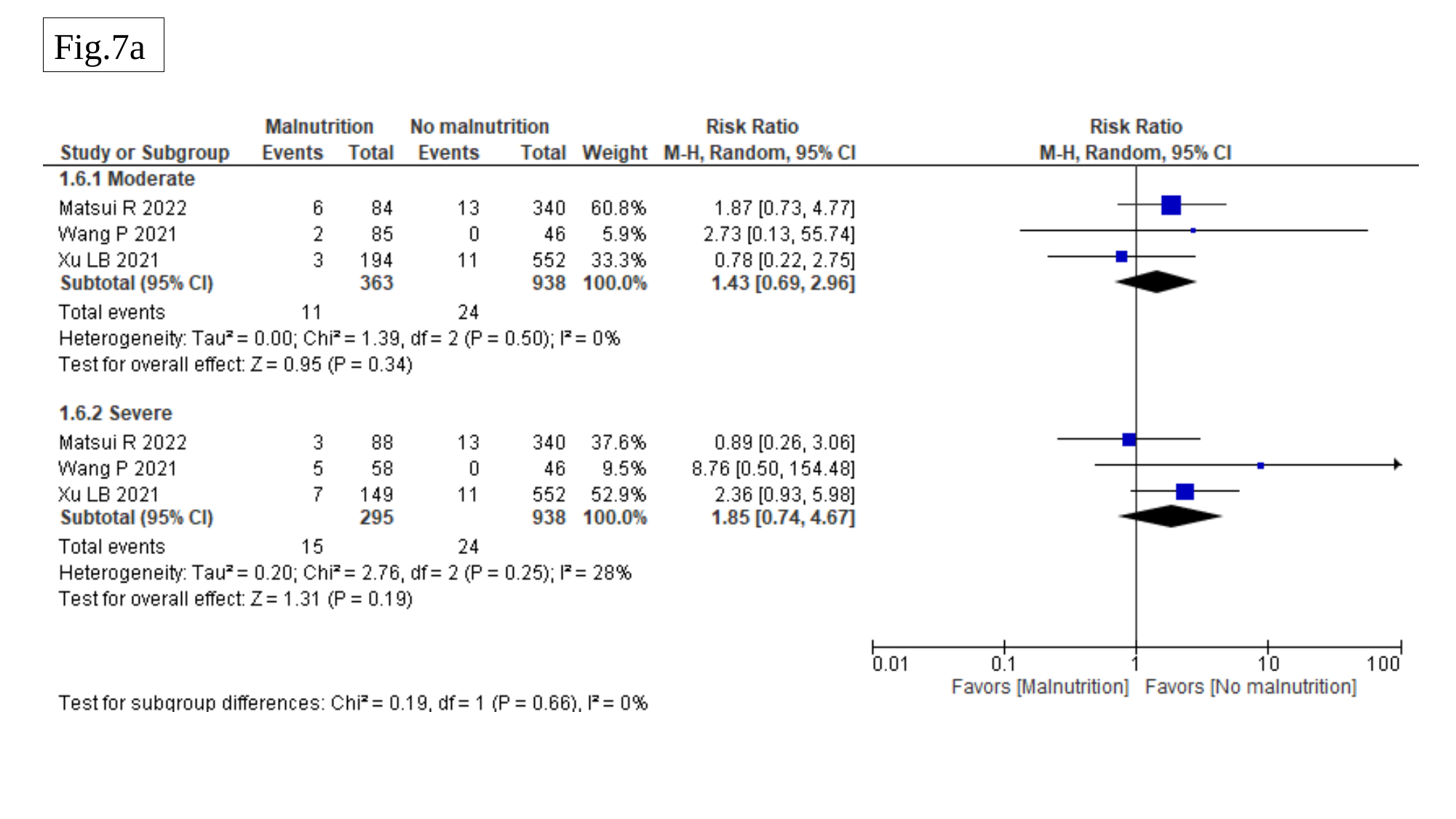

Fig.7a

## Slide 30
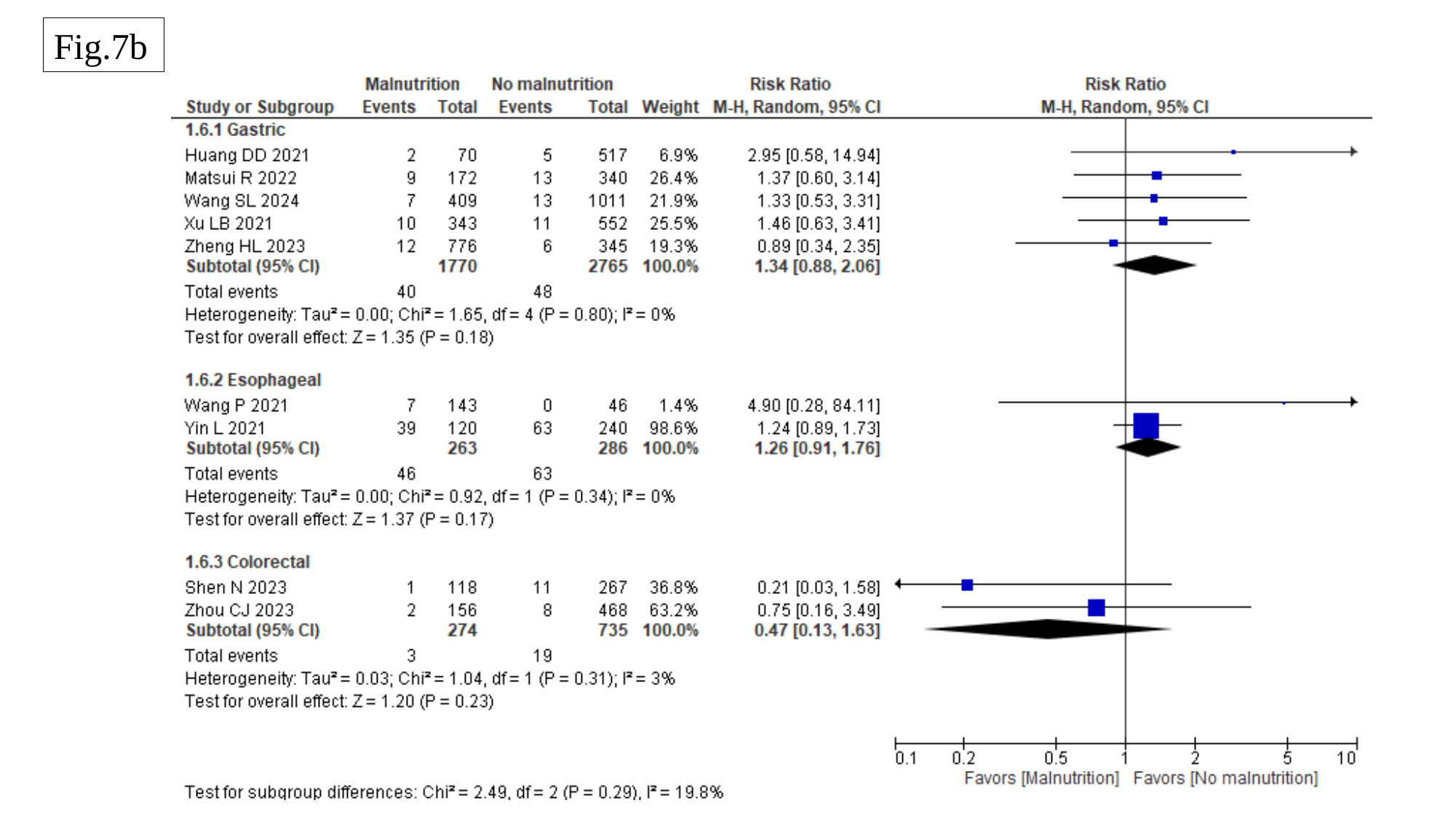

Fig.7b

## Slide 31
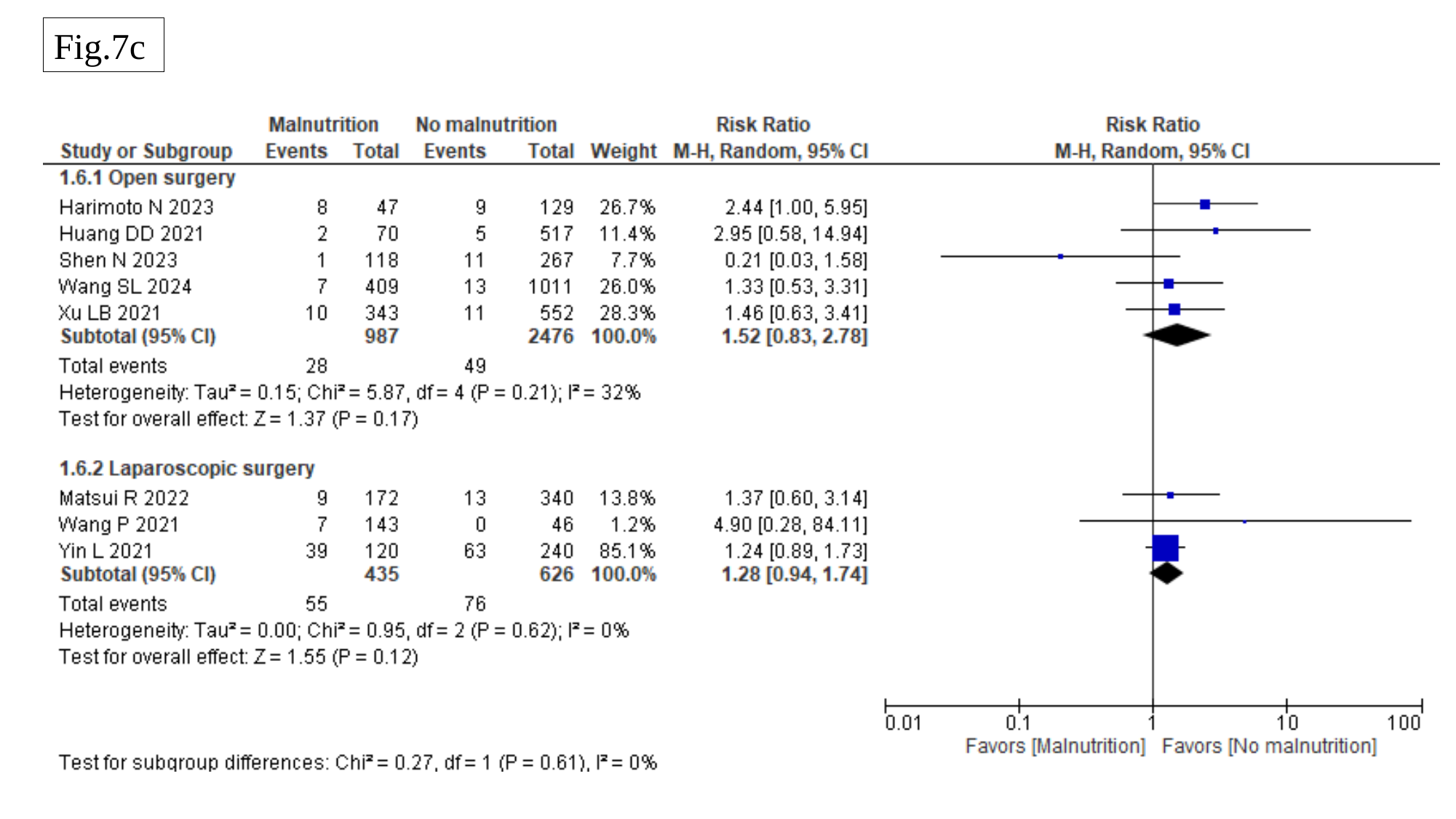

Fig.7c

## Slide 32
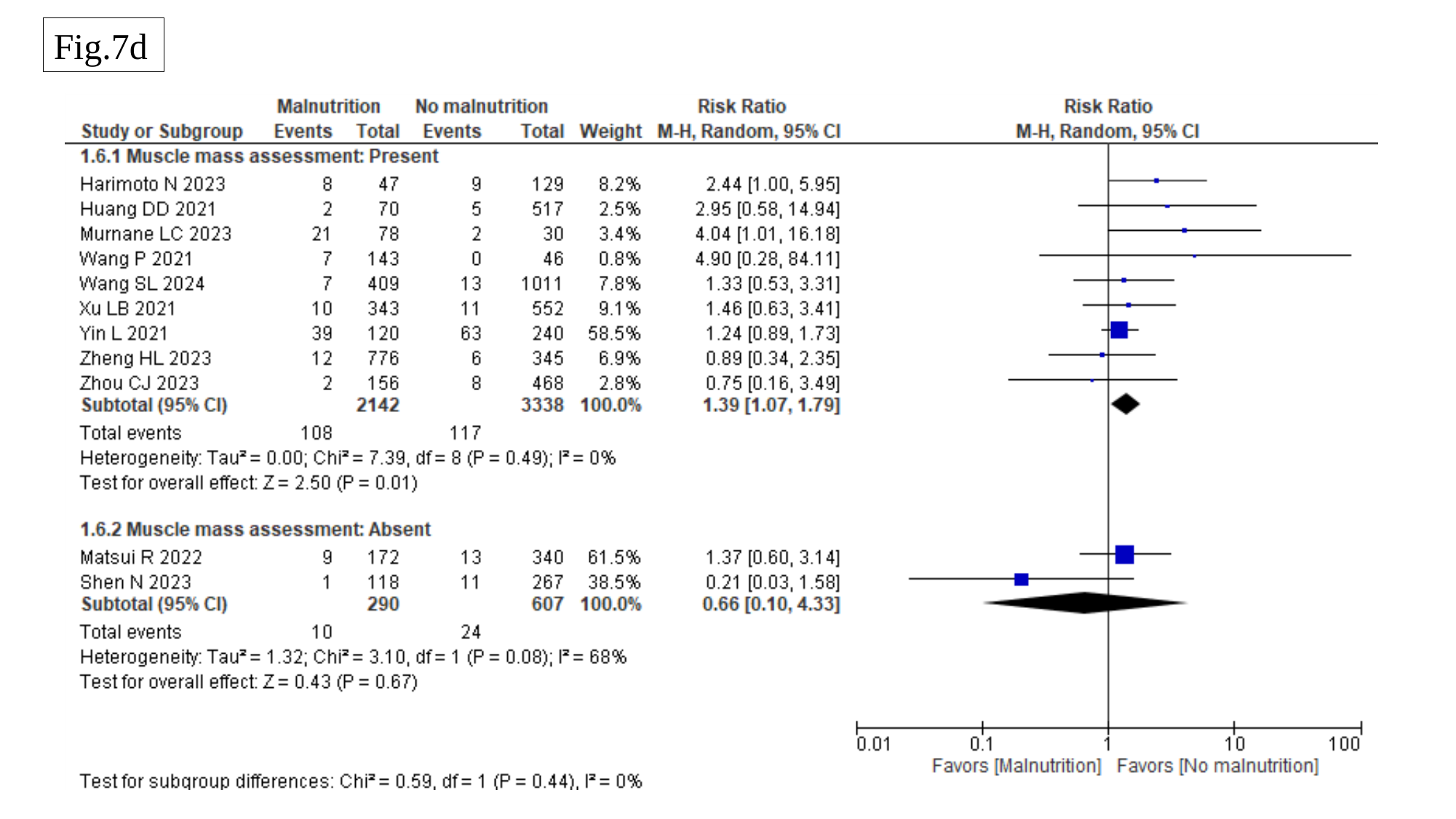

Fig.7d

## Slide 33
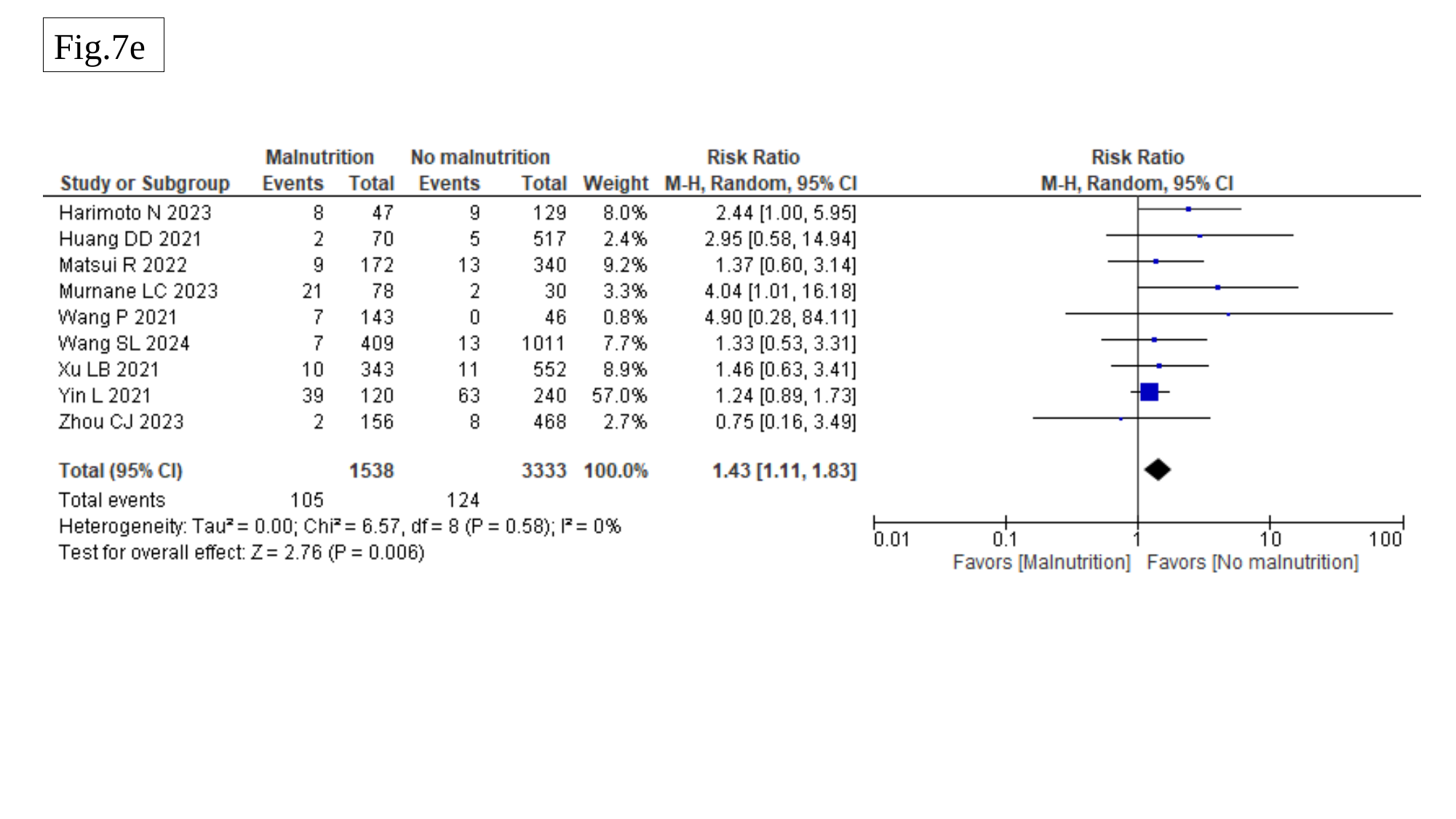

Fig.7e

## Slide 34
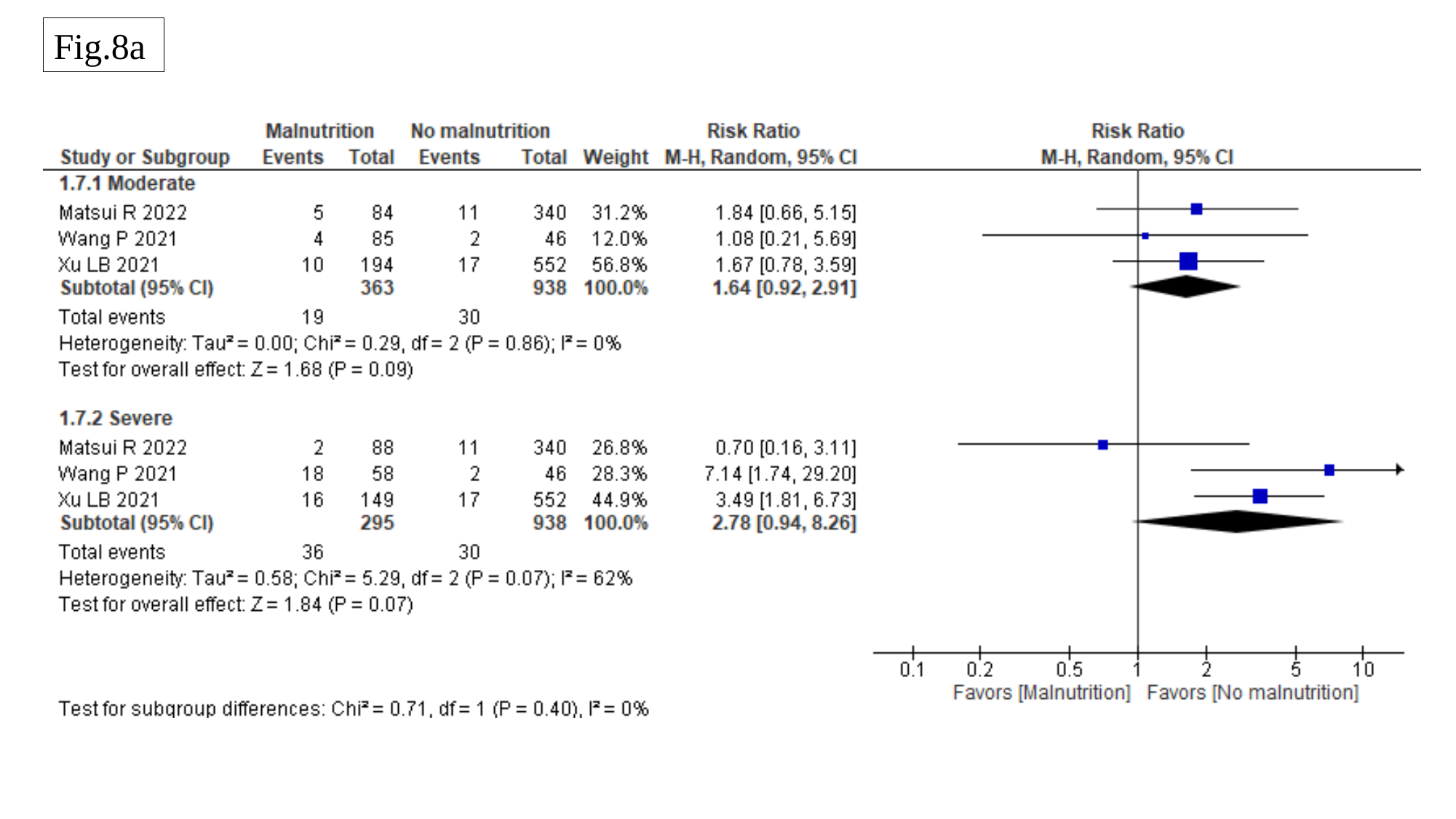

Fig.8a

## Slide 35
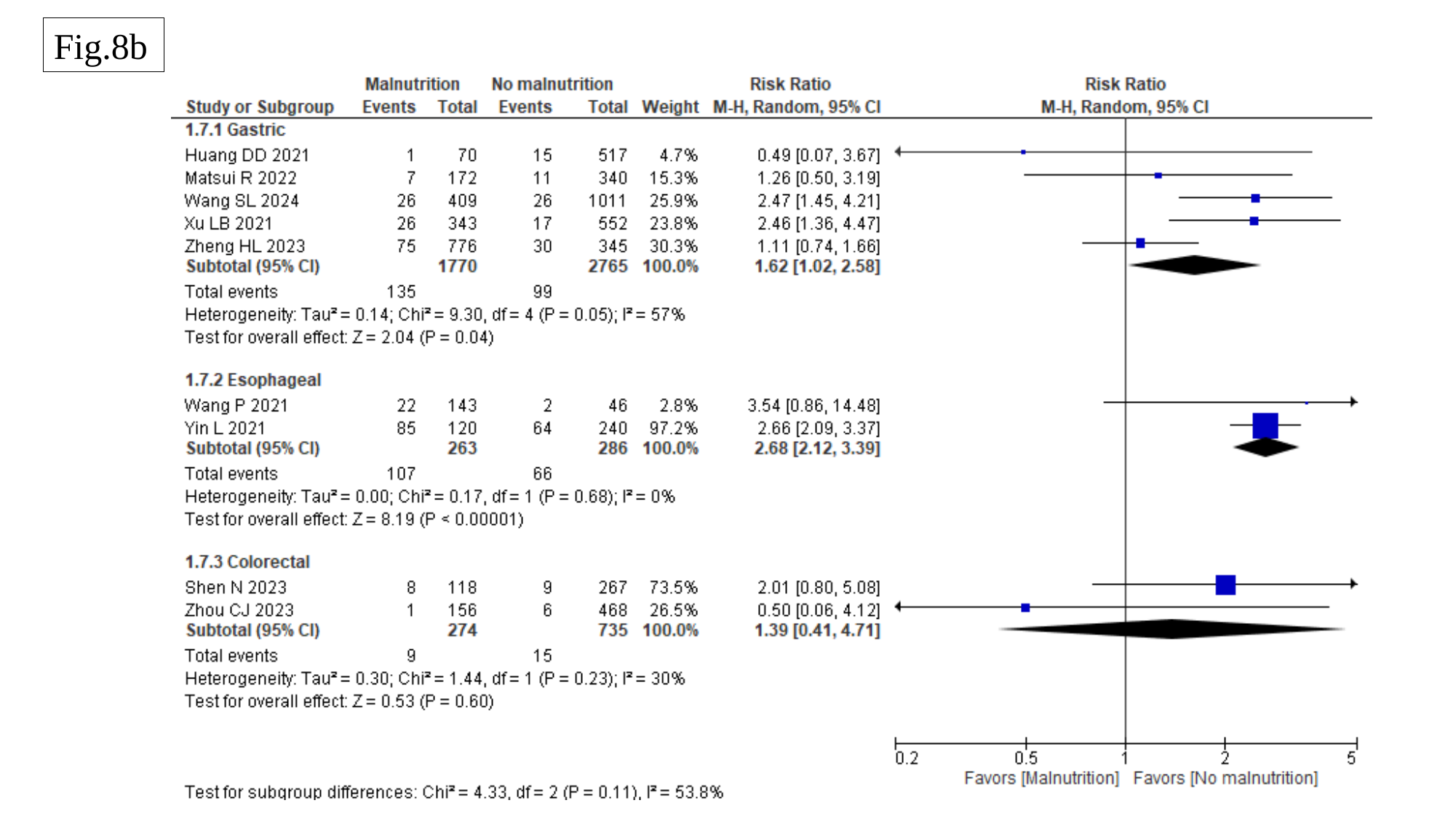

Fig.8b

## Slide 36
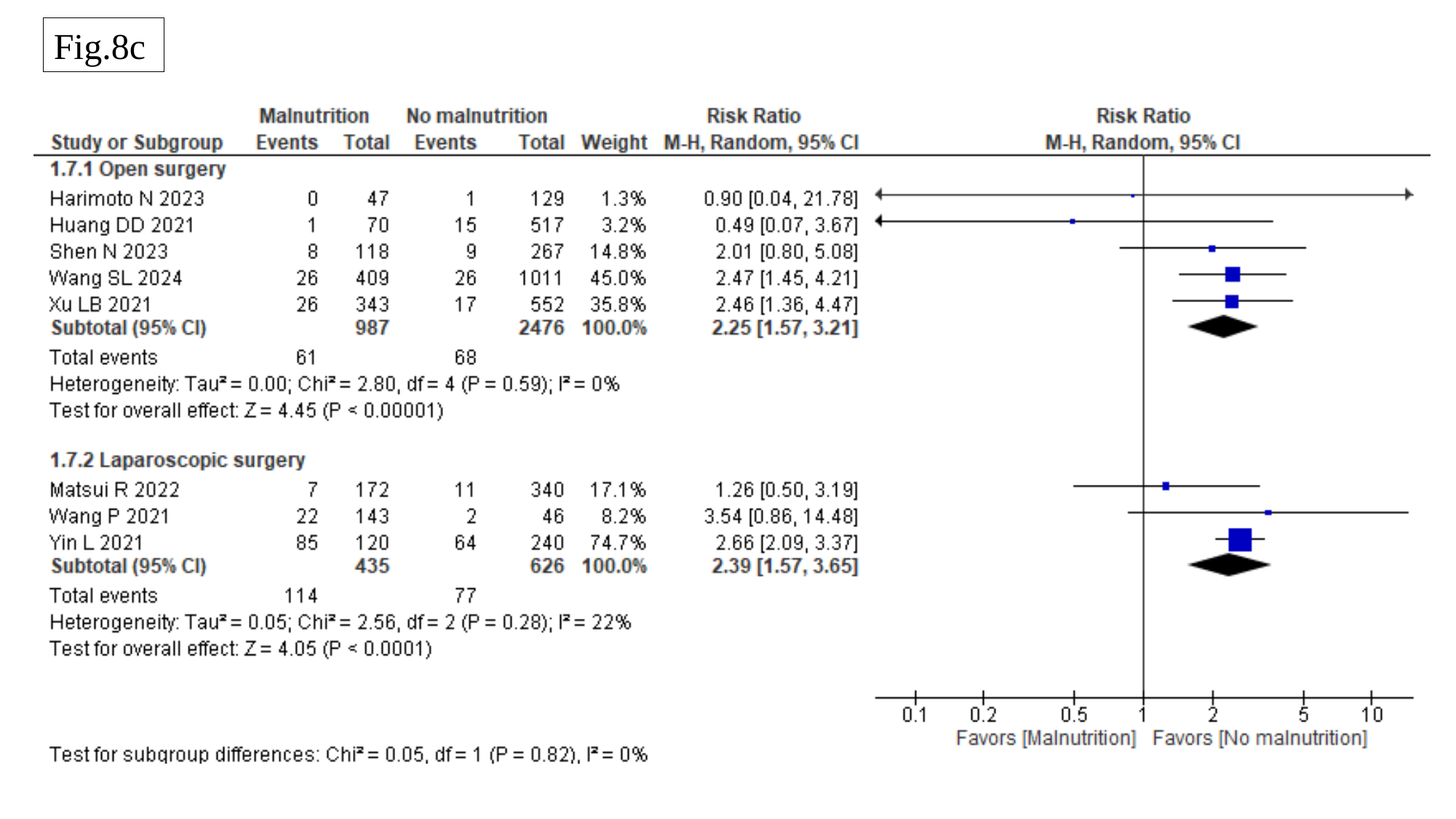

Fig.8c

## Slide 37
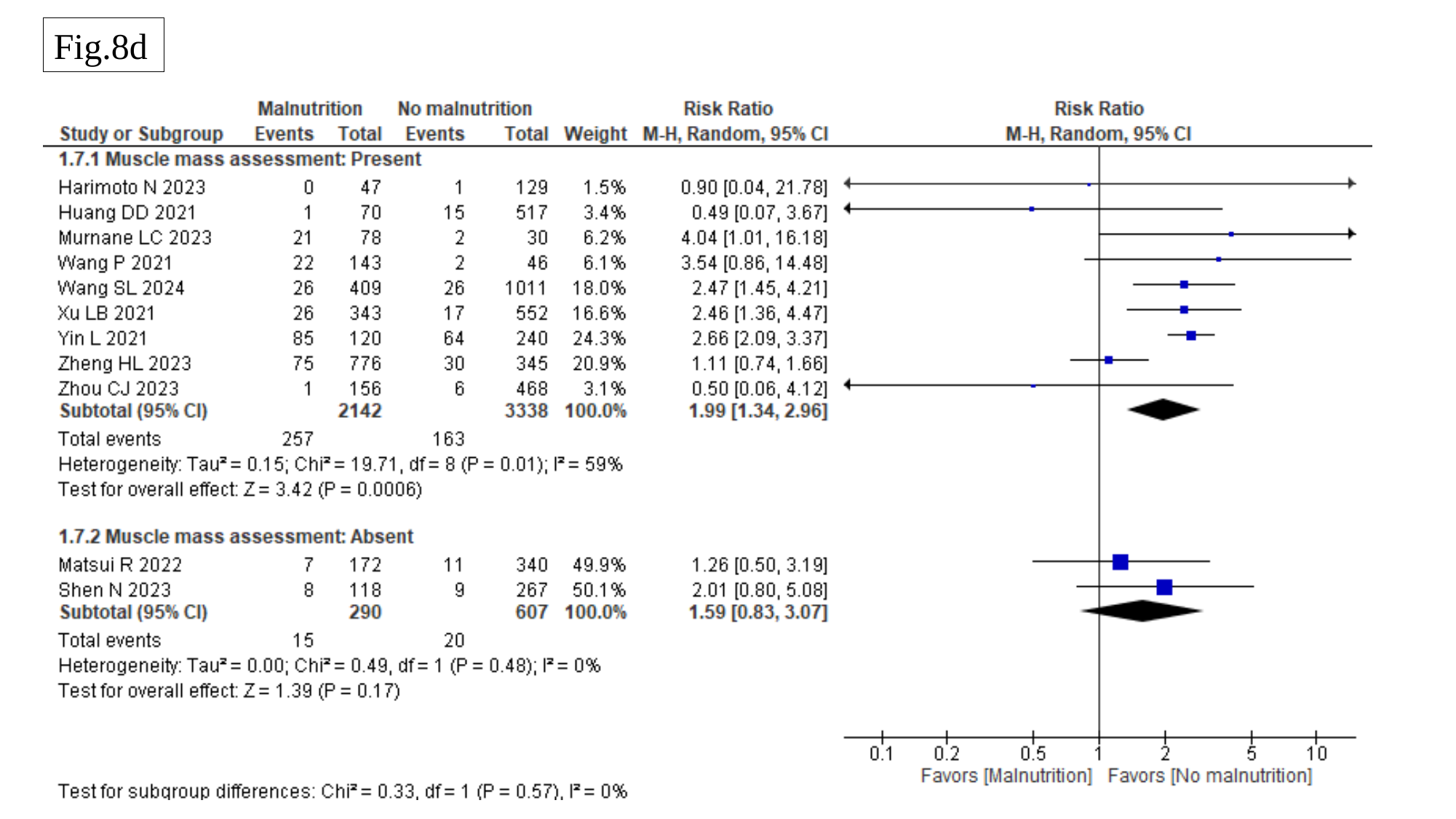

Fig.8d

## Slide 38
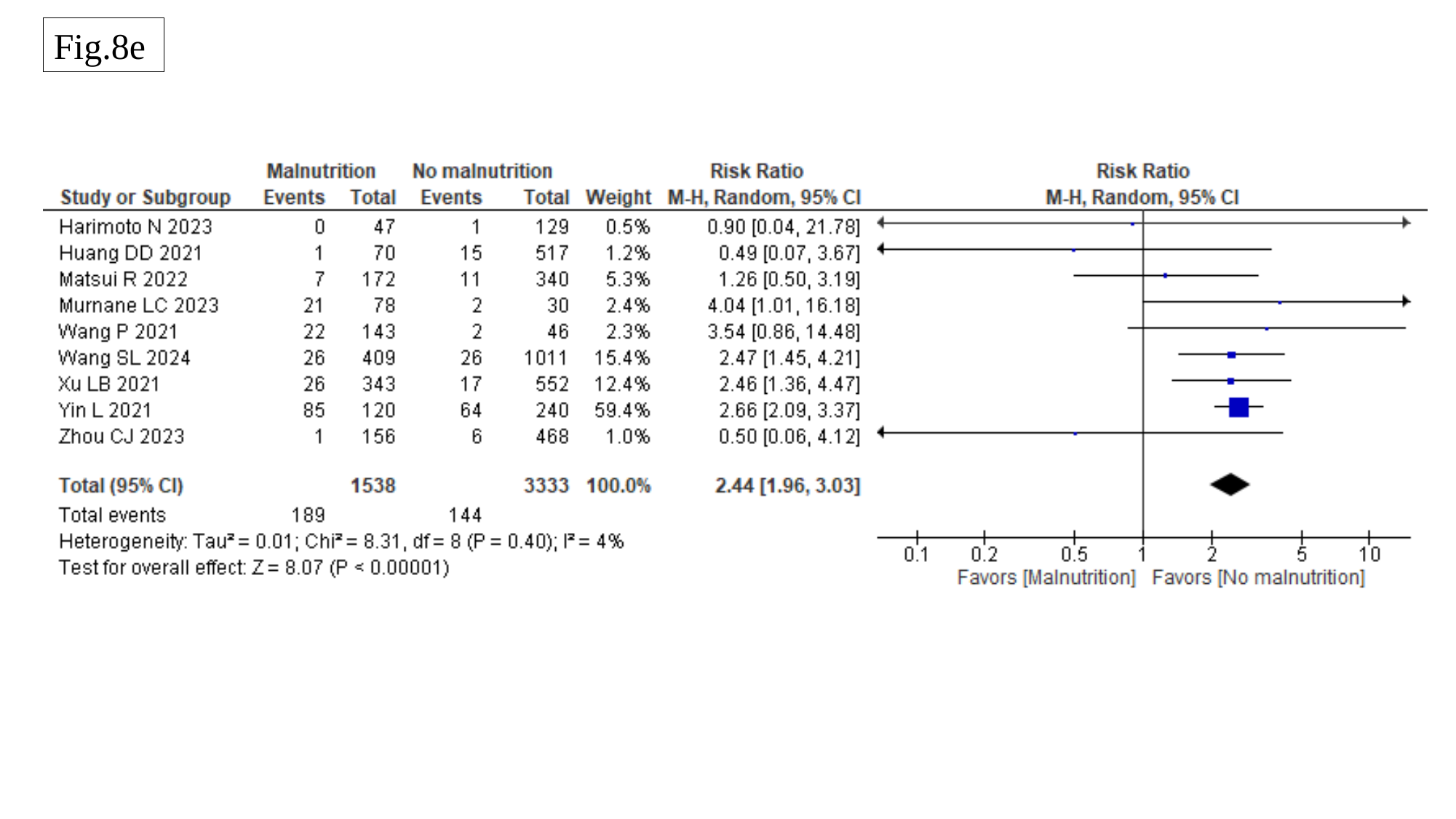

Fig.8e

## Slide 39
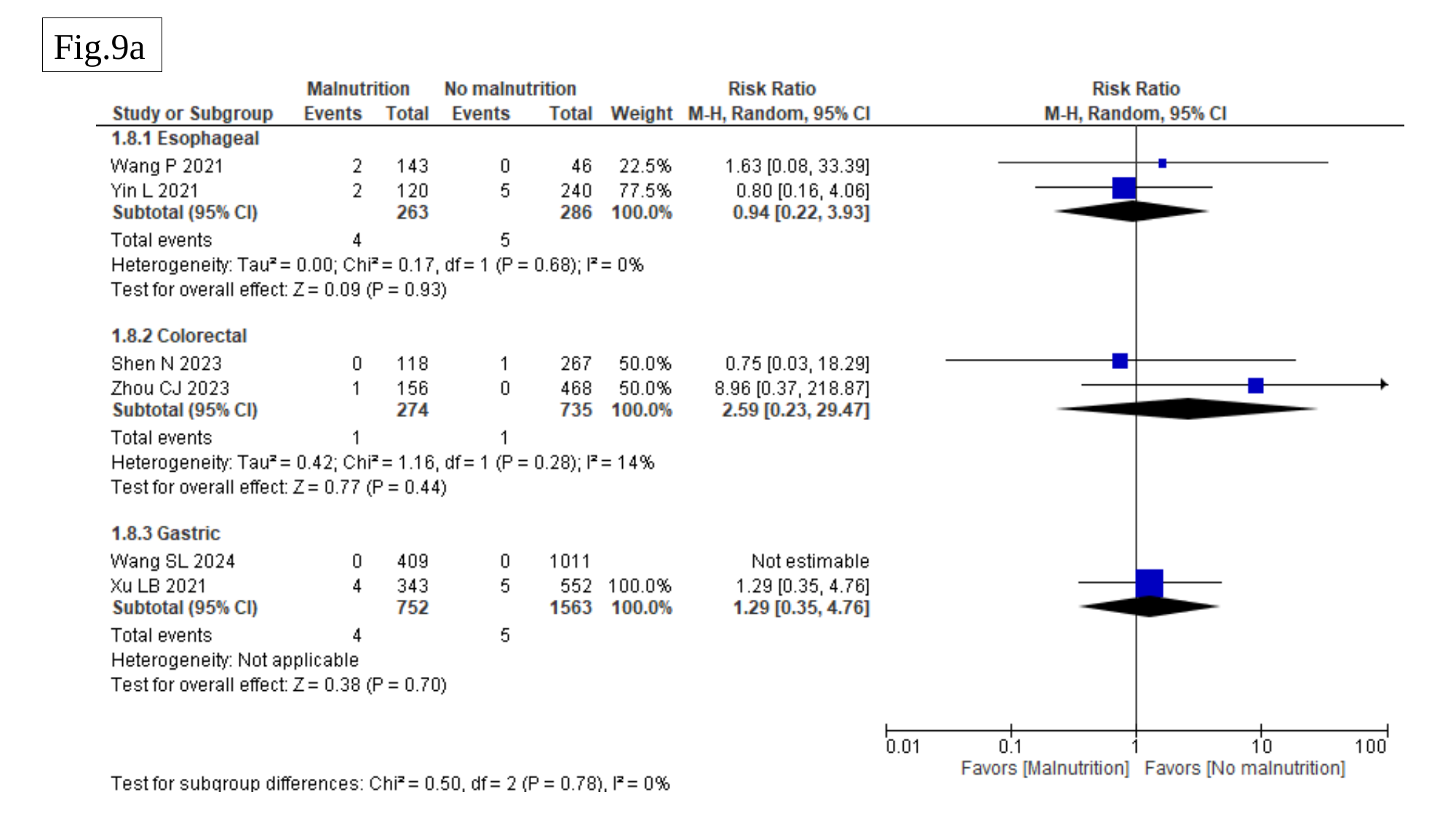

Fig.9a

## Slide 40
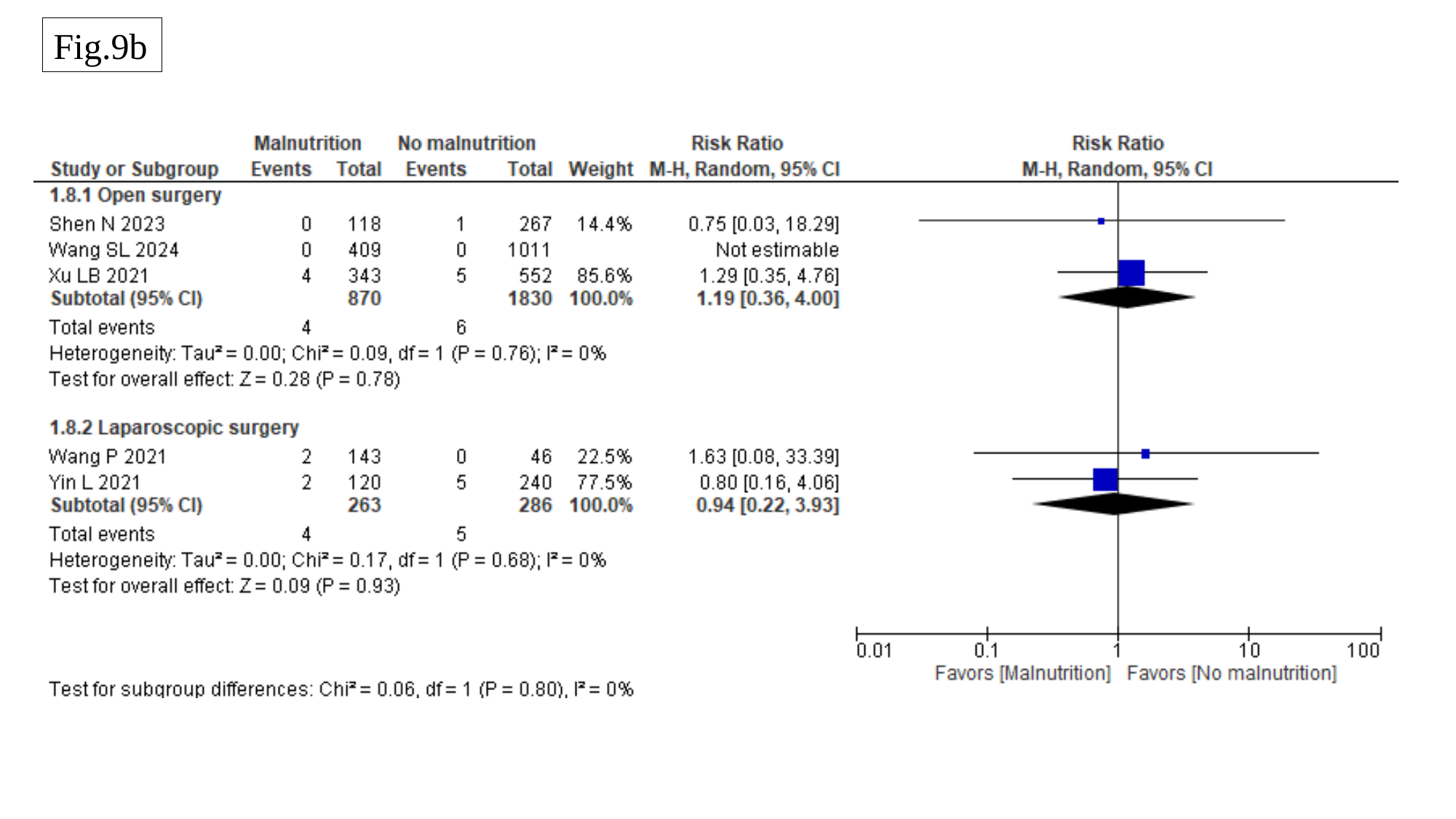

Fig.9b

## Slide 41
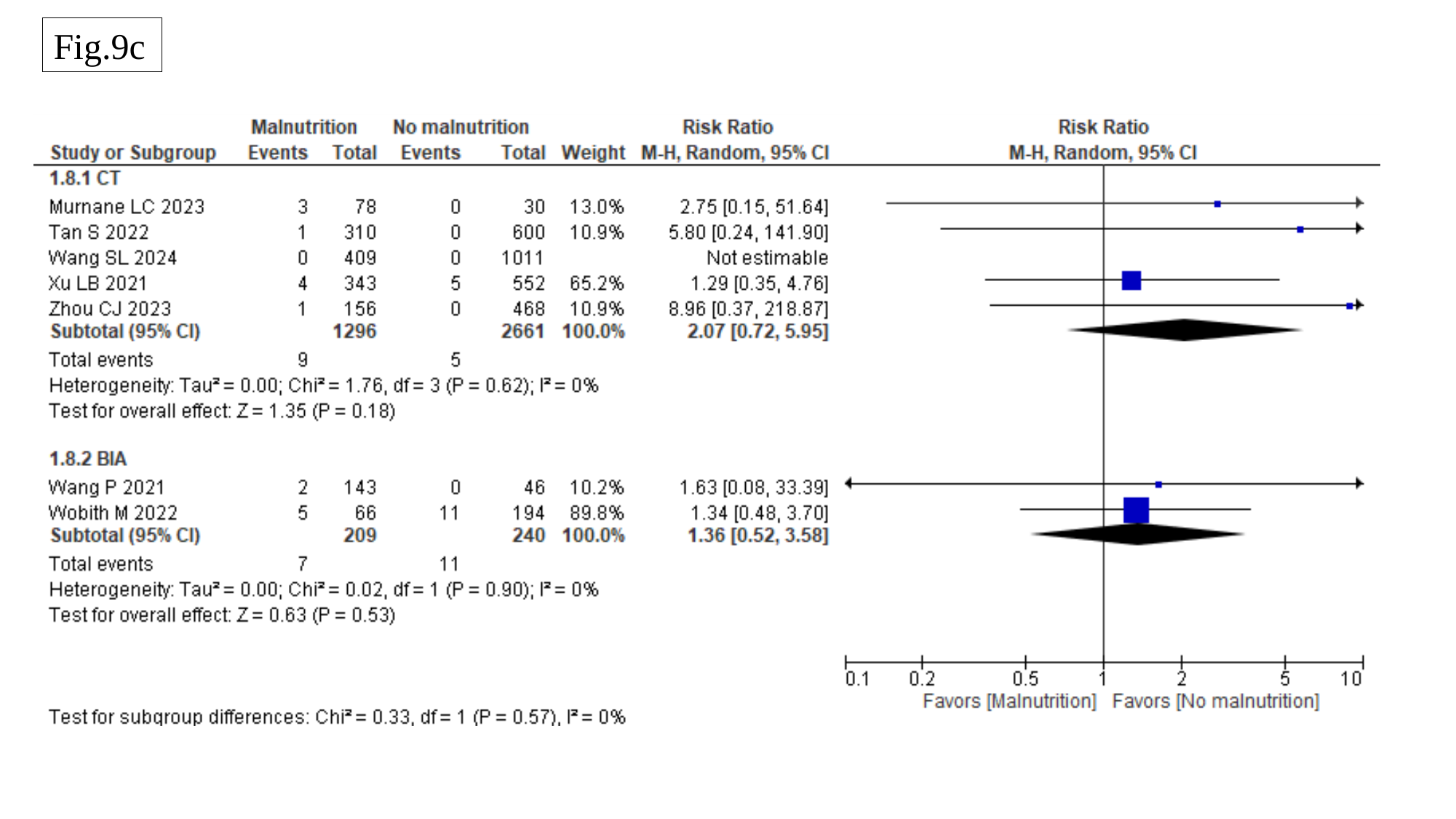

Fig.9c

## Slide 42
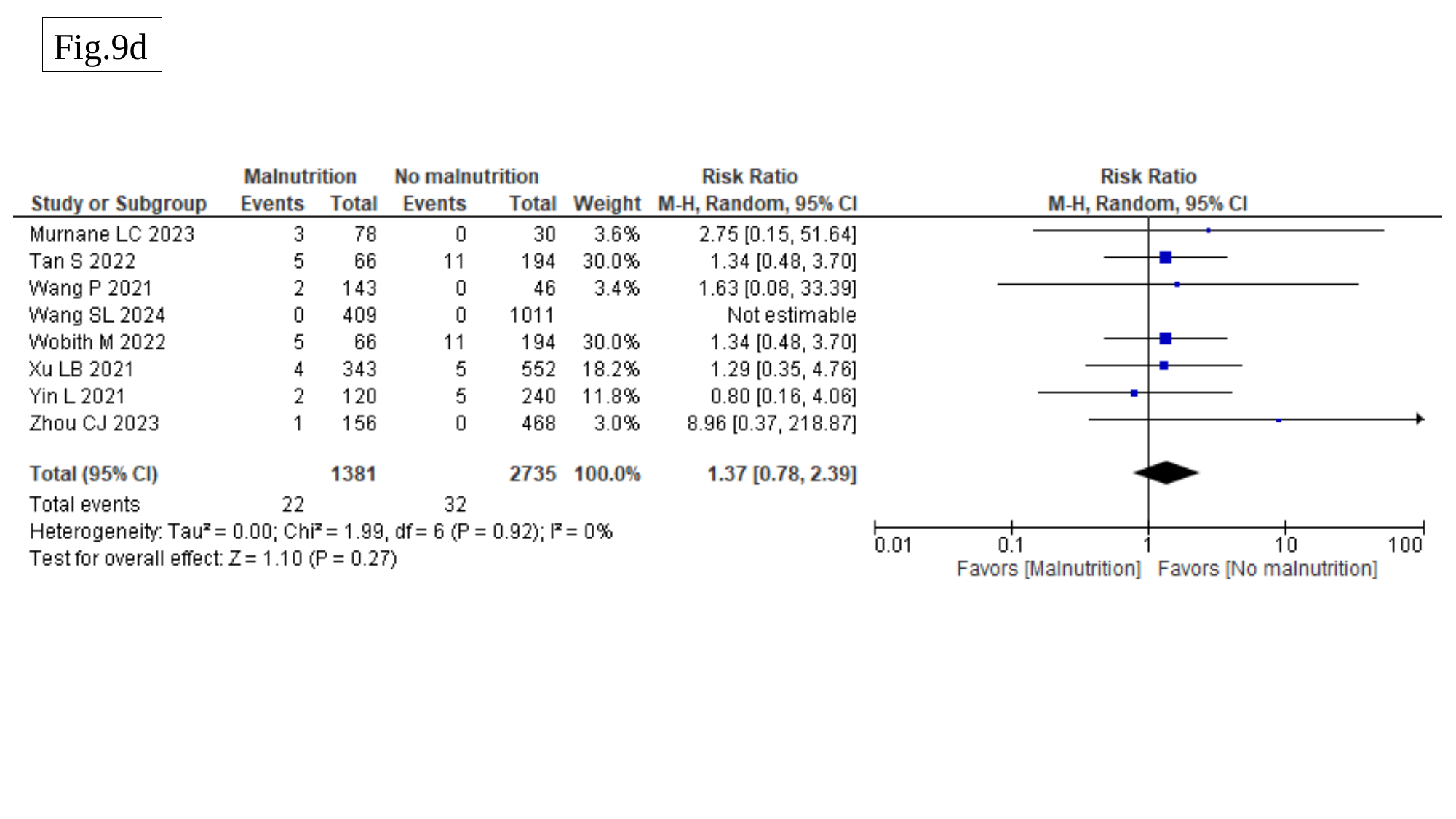

Fig.9d

## Slide 43
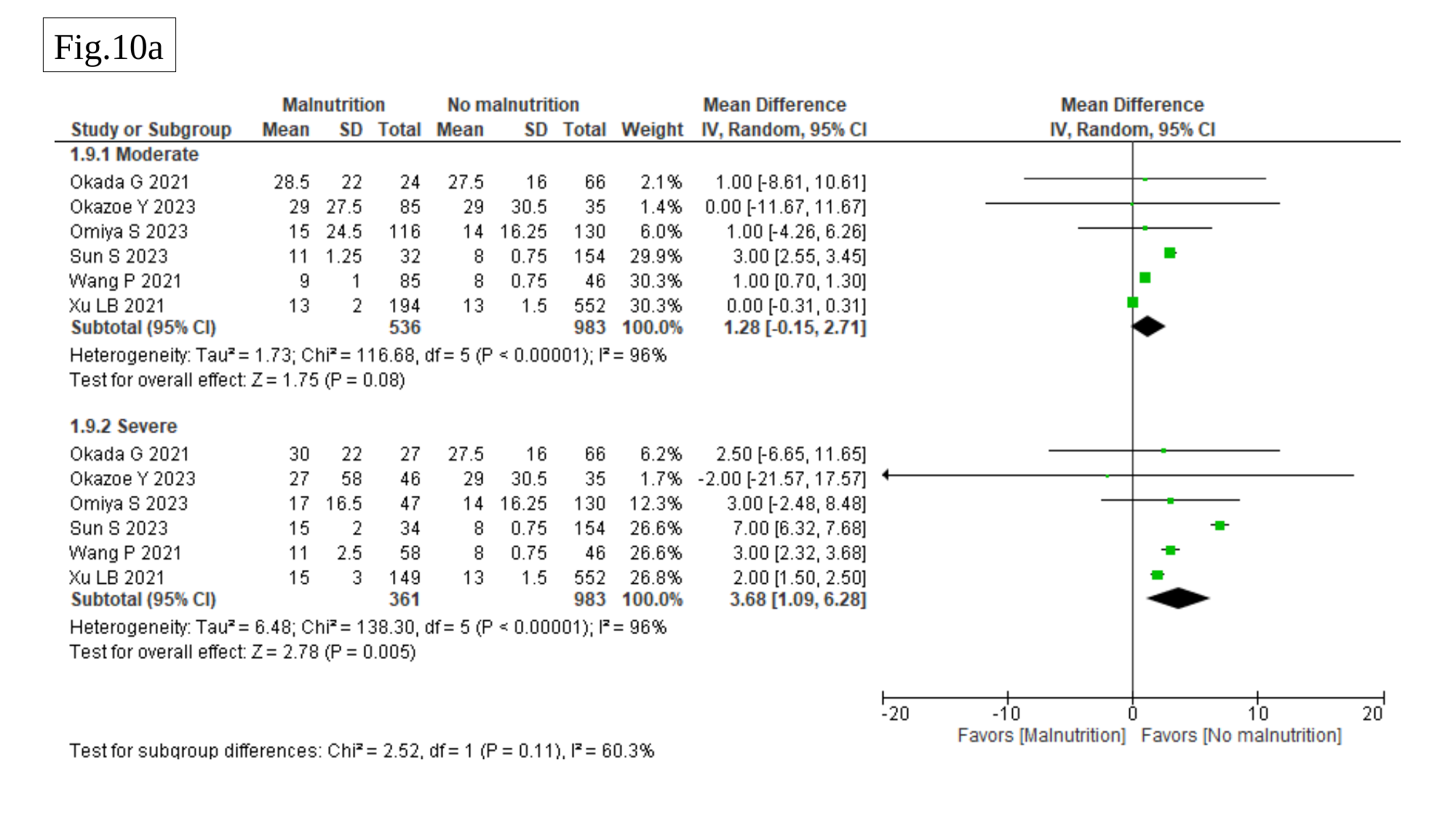

Fig.10a

## Slide 44
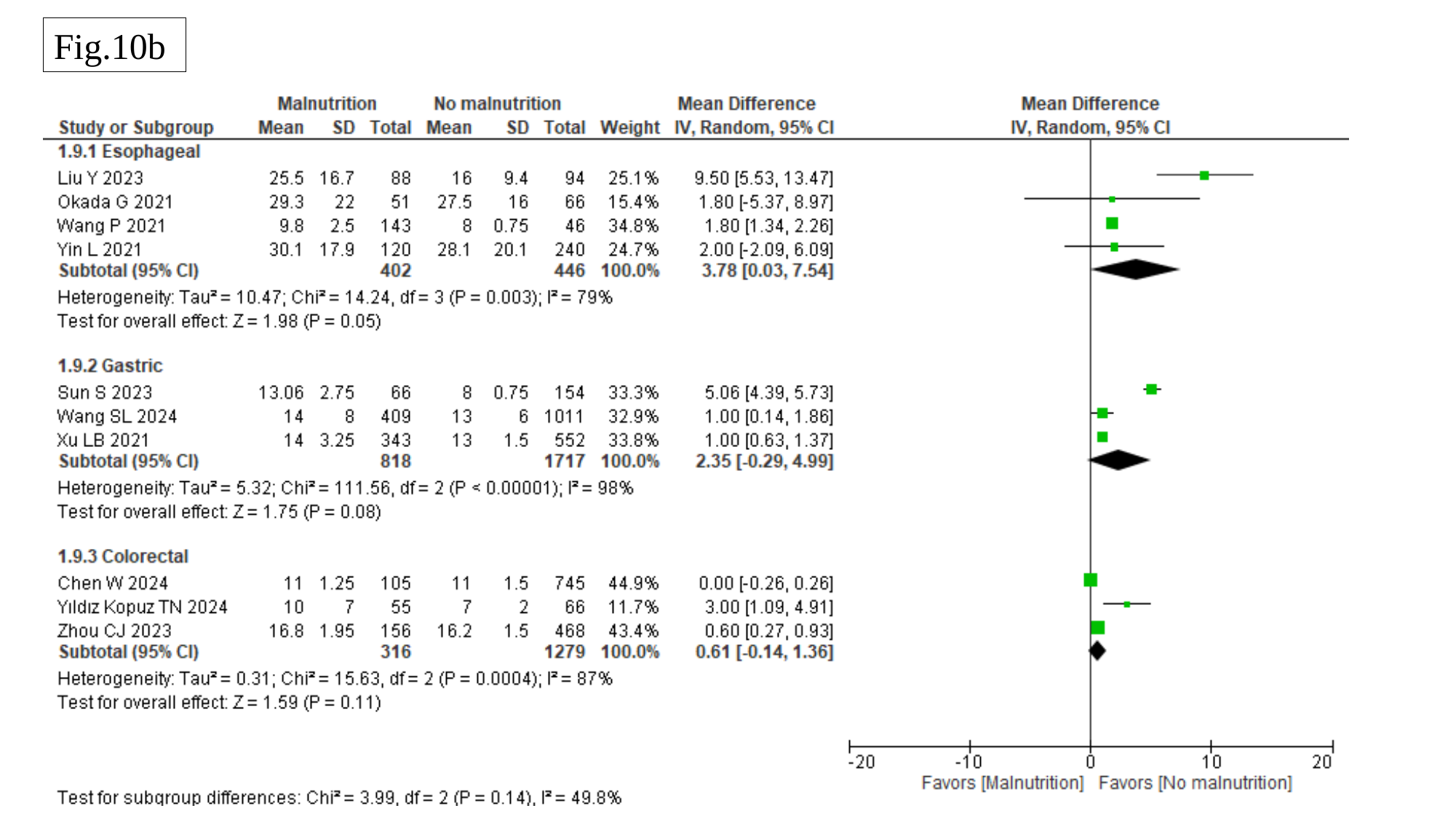

Fig.10b

## Slide 45
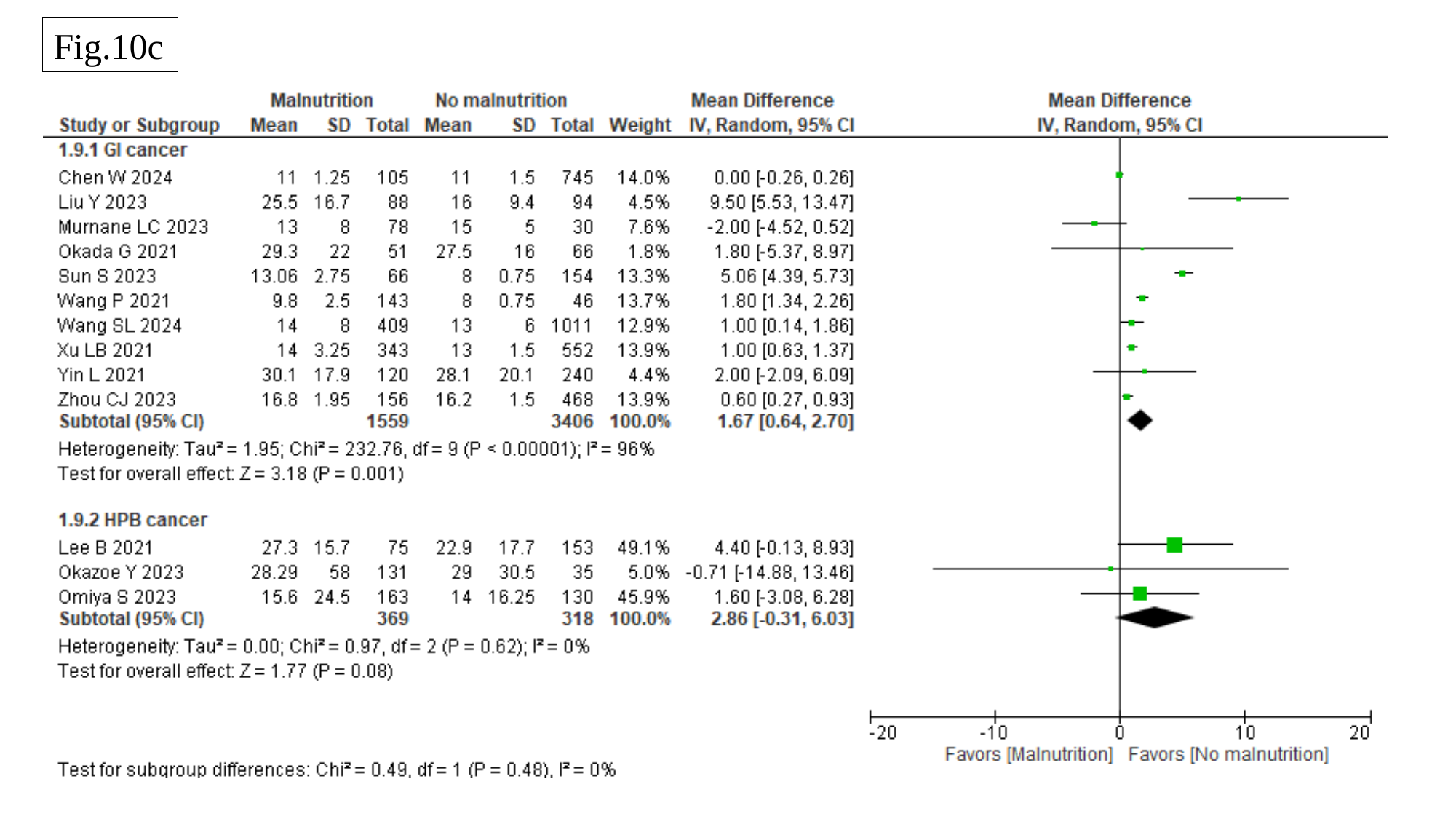

Fig.10c

## Slide 46
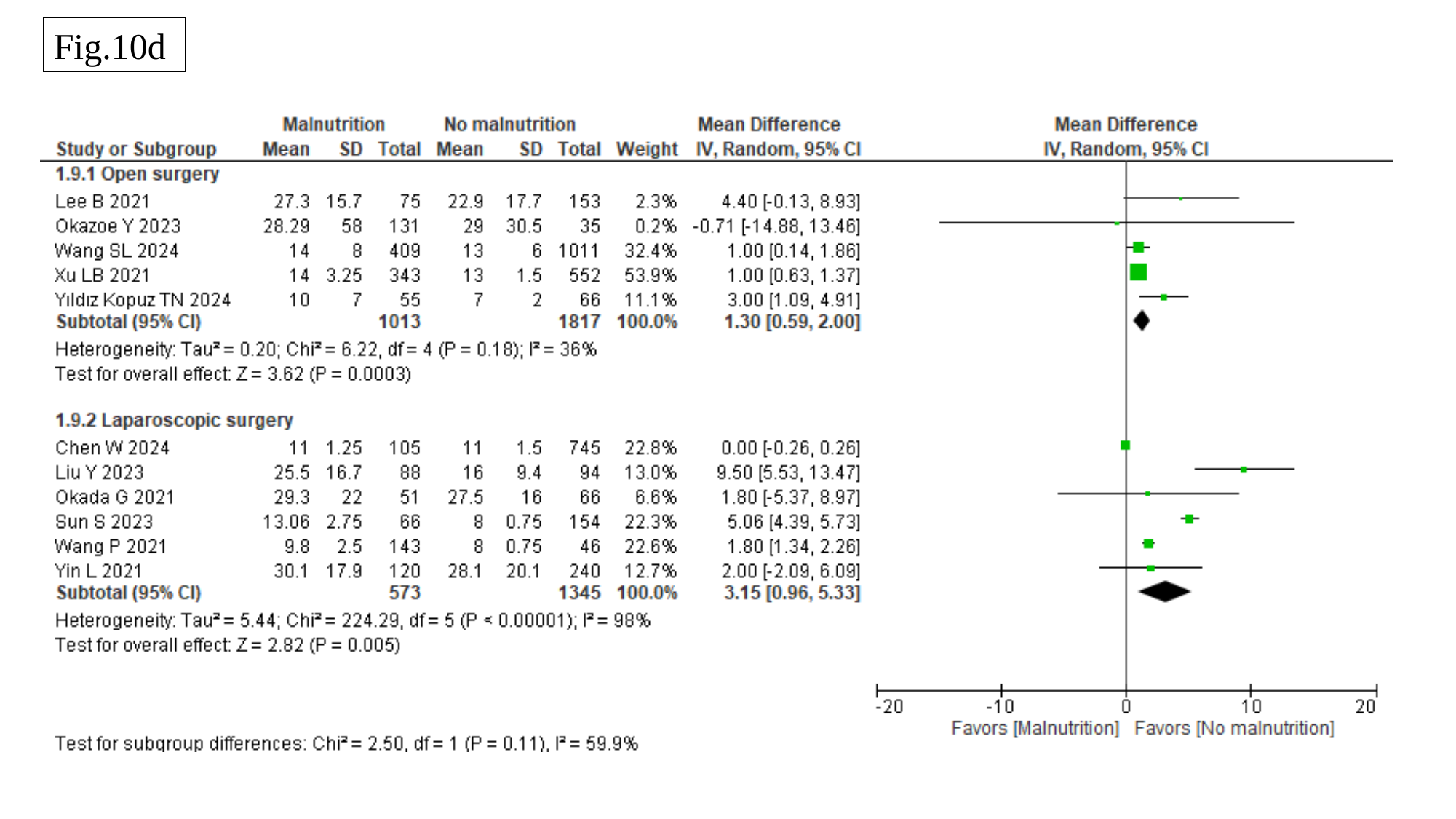

Fig.10d

## Slide 47
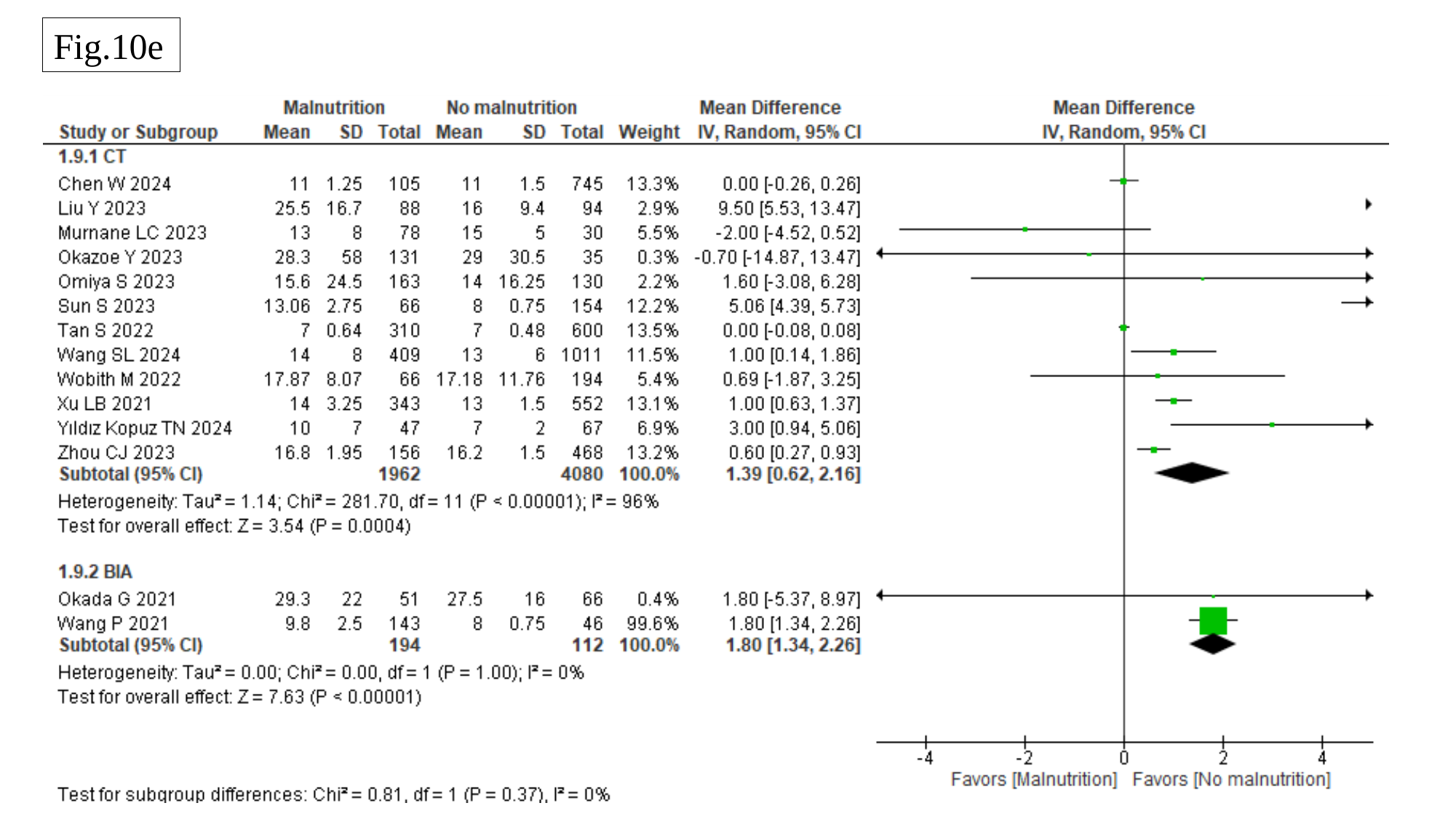

Fig.10e

## Slide 48
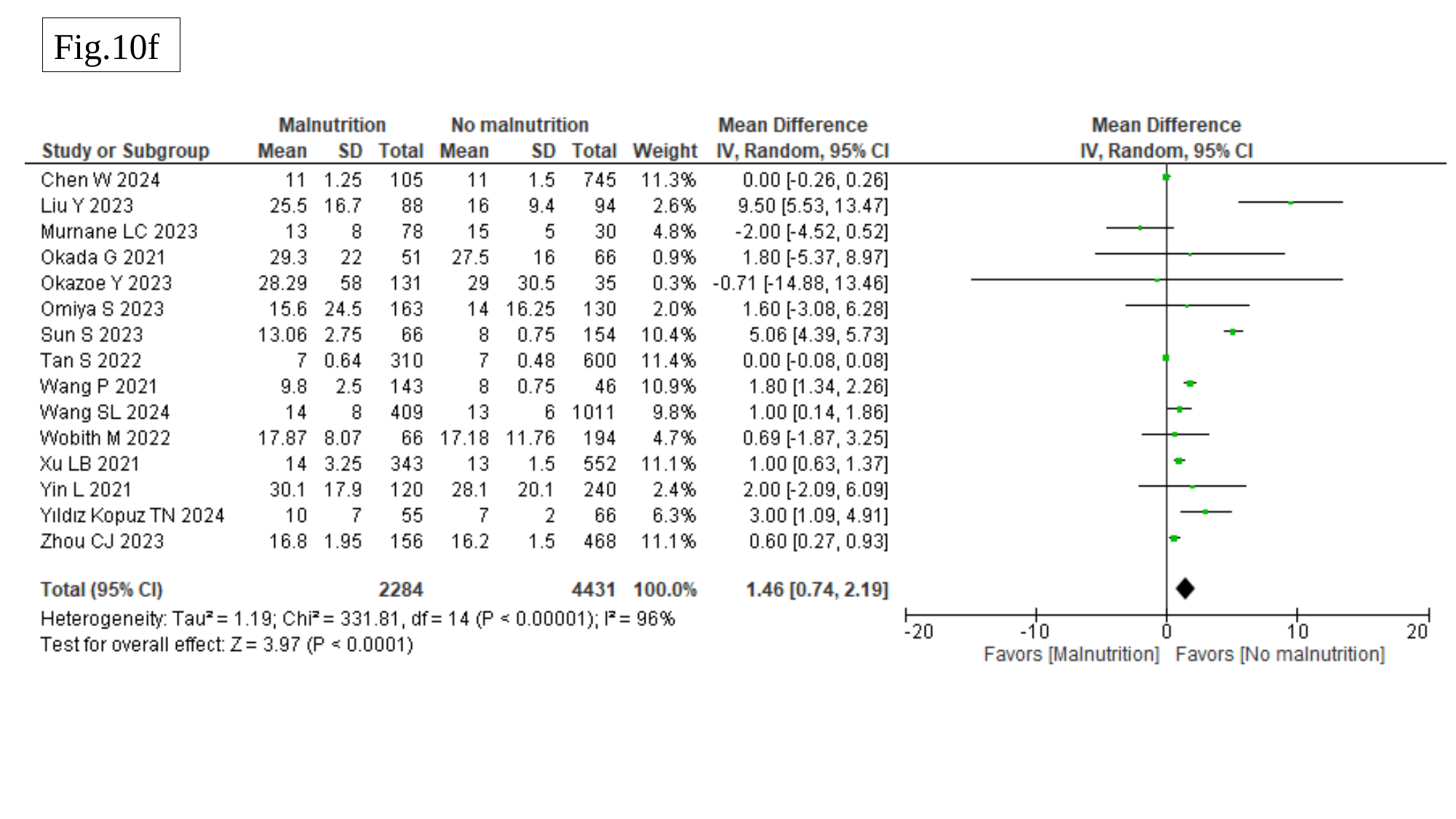

Fig.10f

## Slide 49
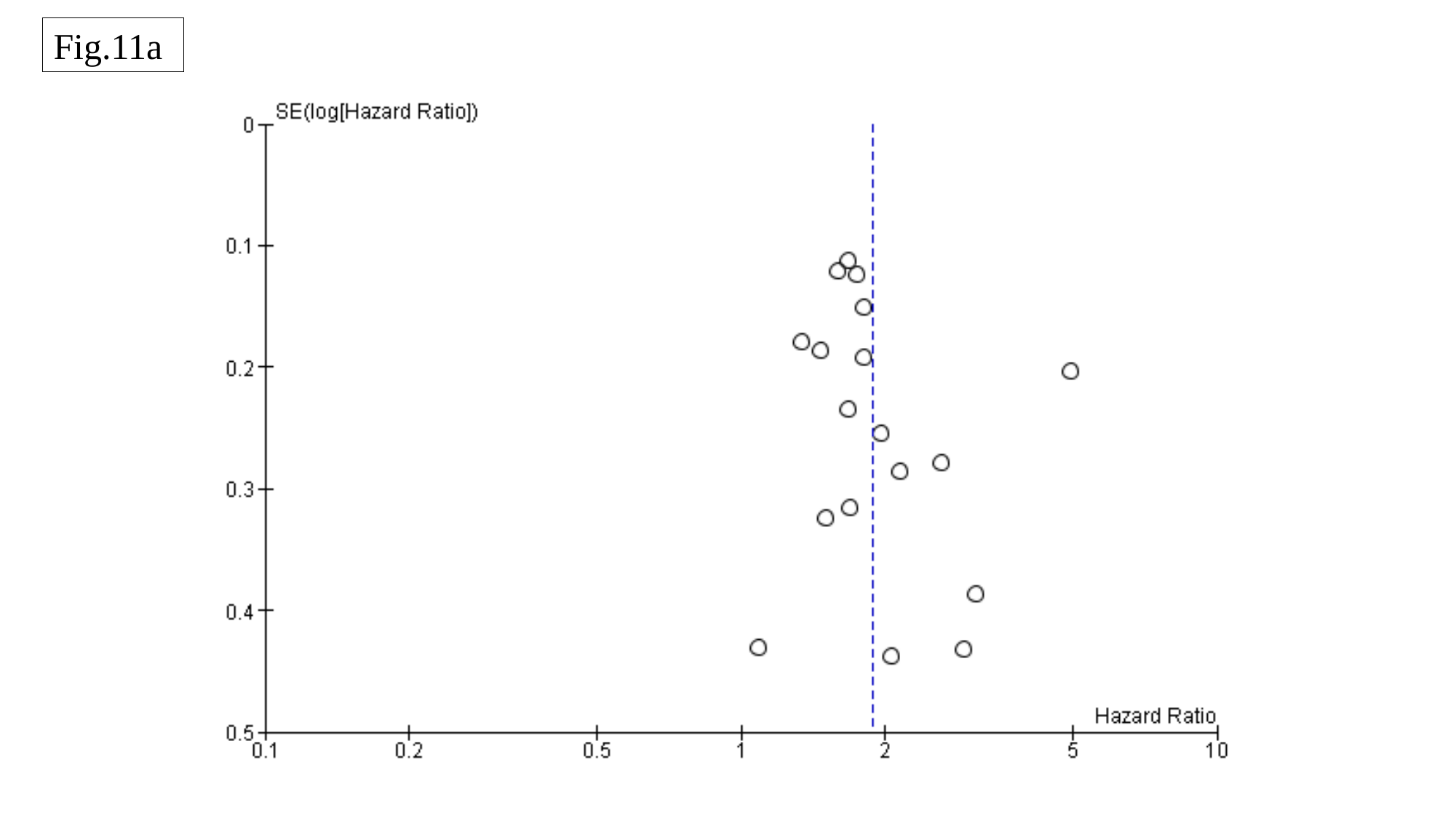

Fig.11a

## Slide 50
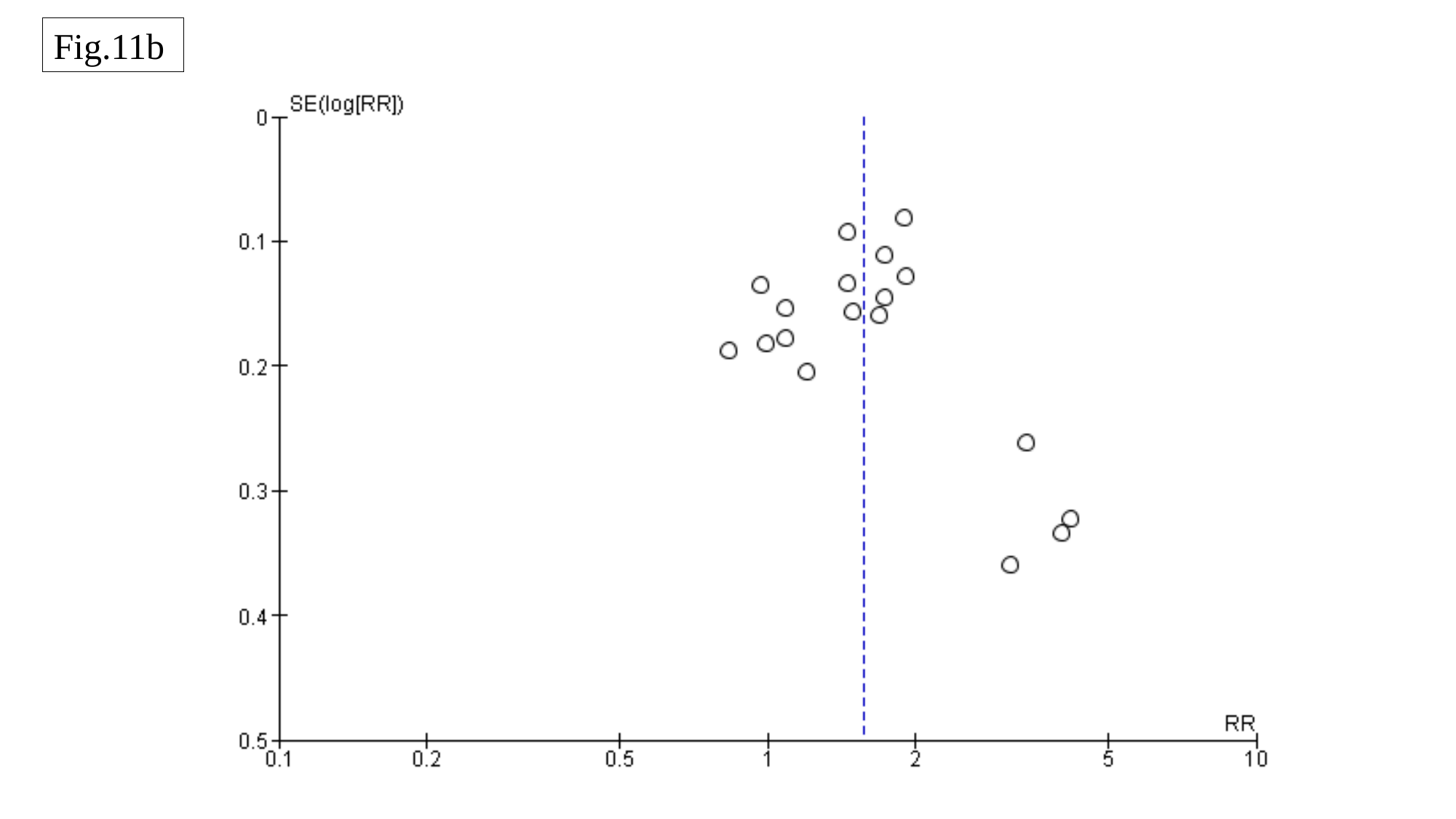

Fig.11b

## Slide 51
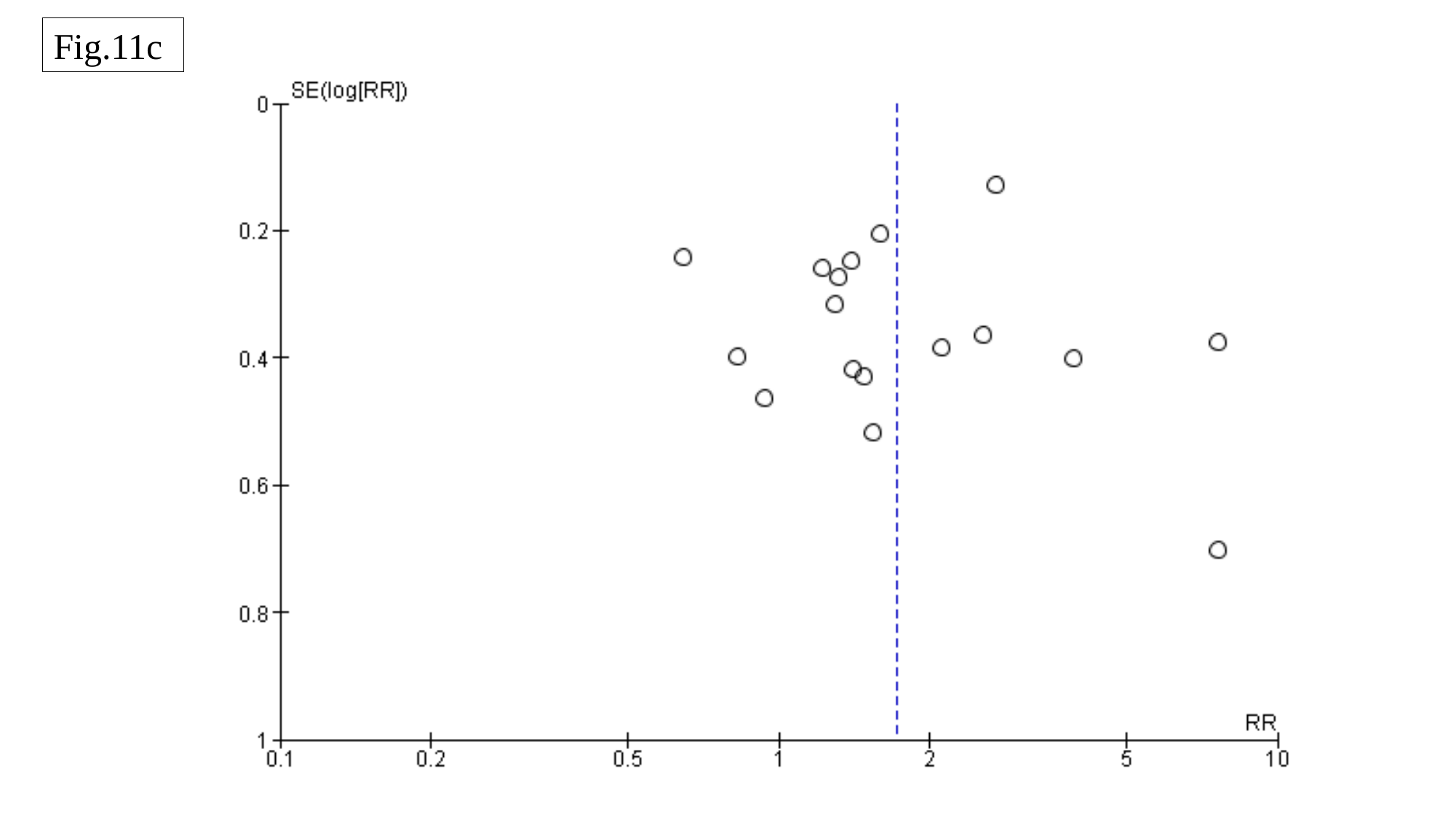

Fig.11c

## Slide 52
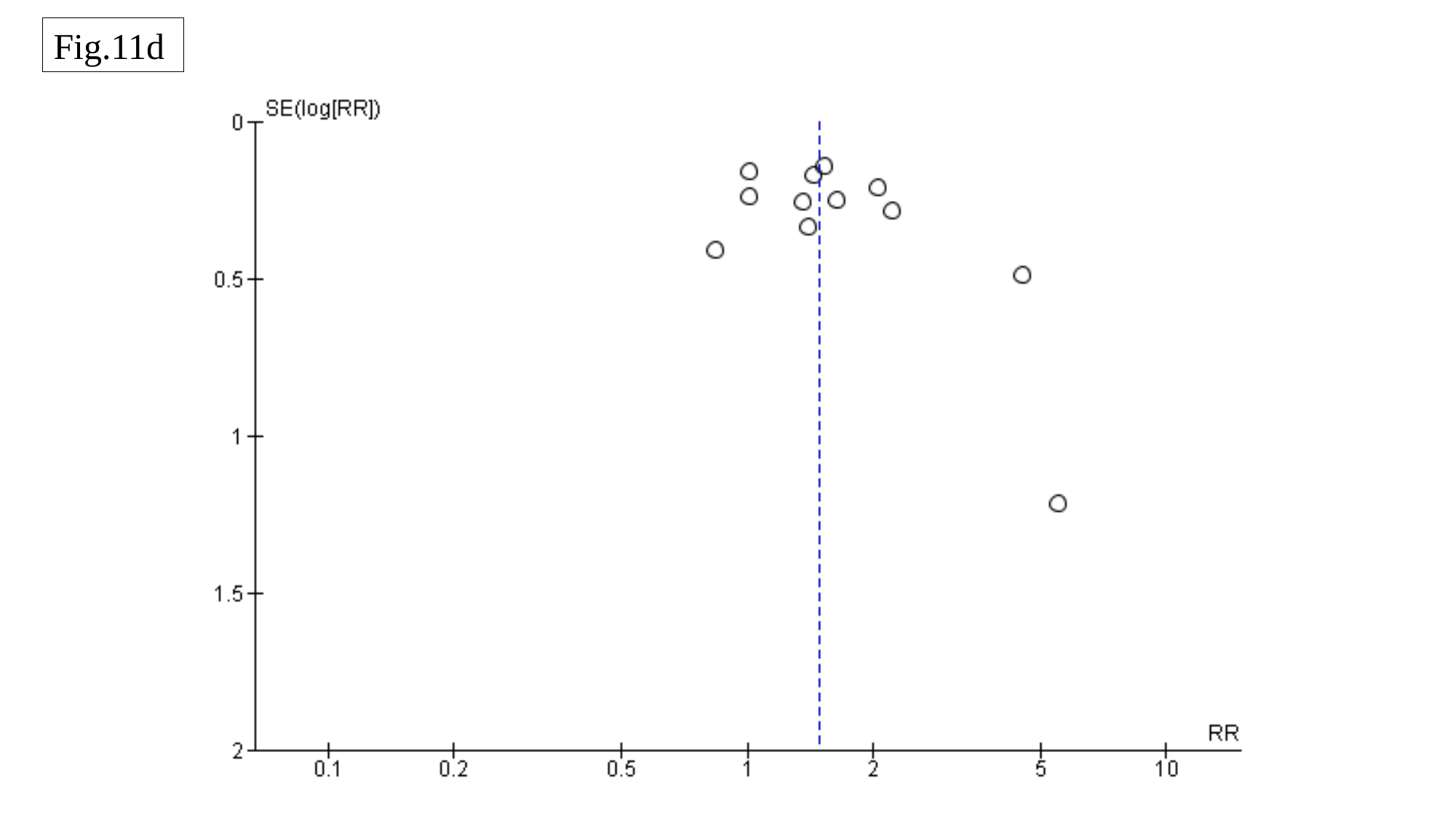

Fig.11d

## Slide 53
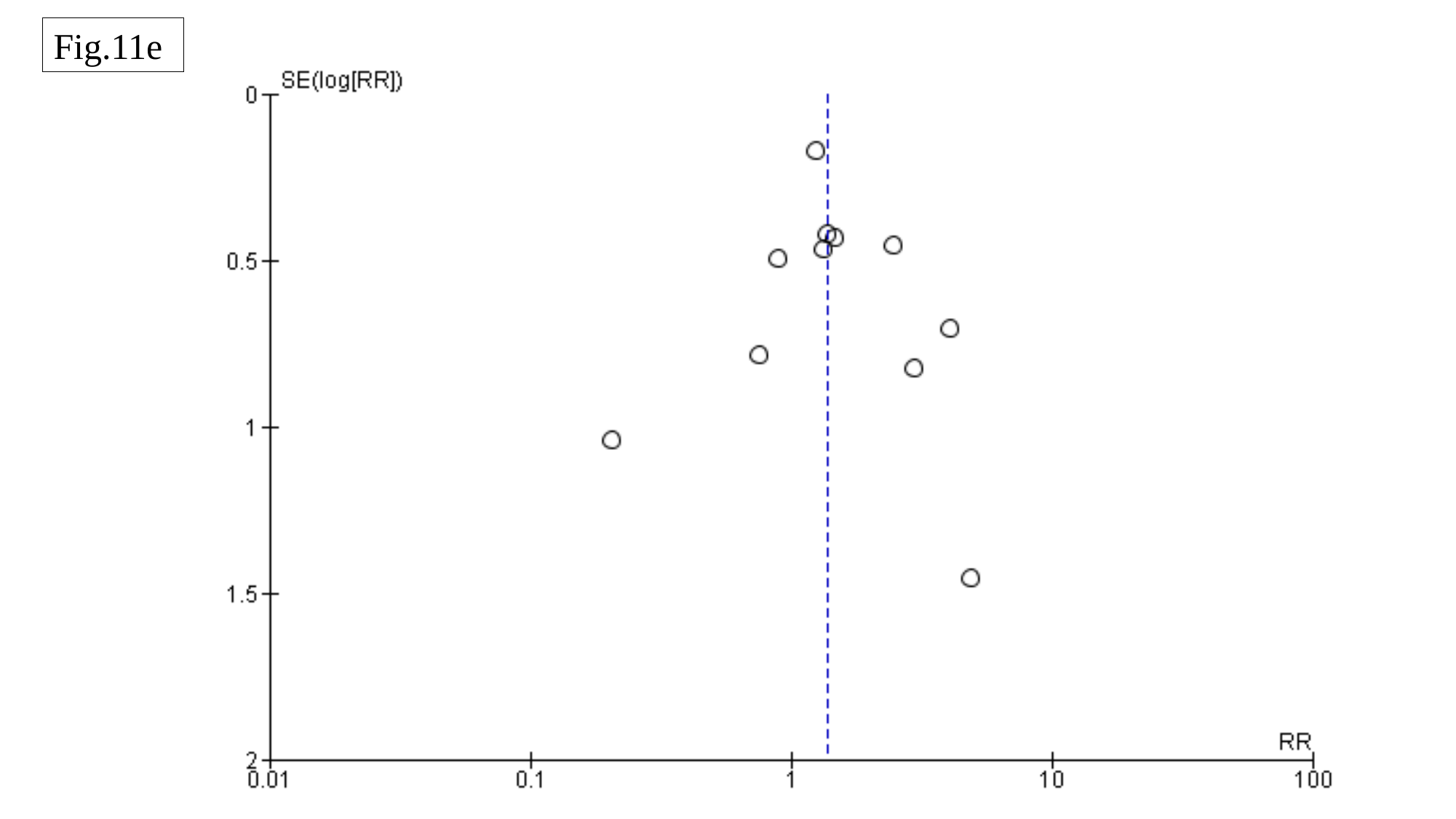

Fig.11e

## Slide 54
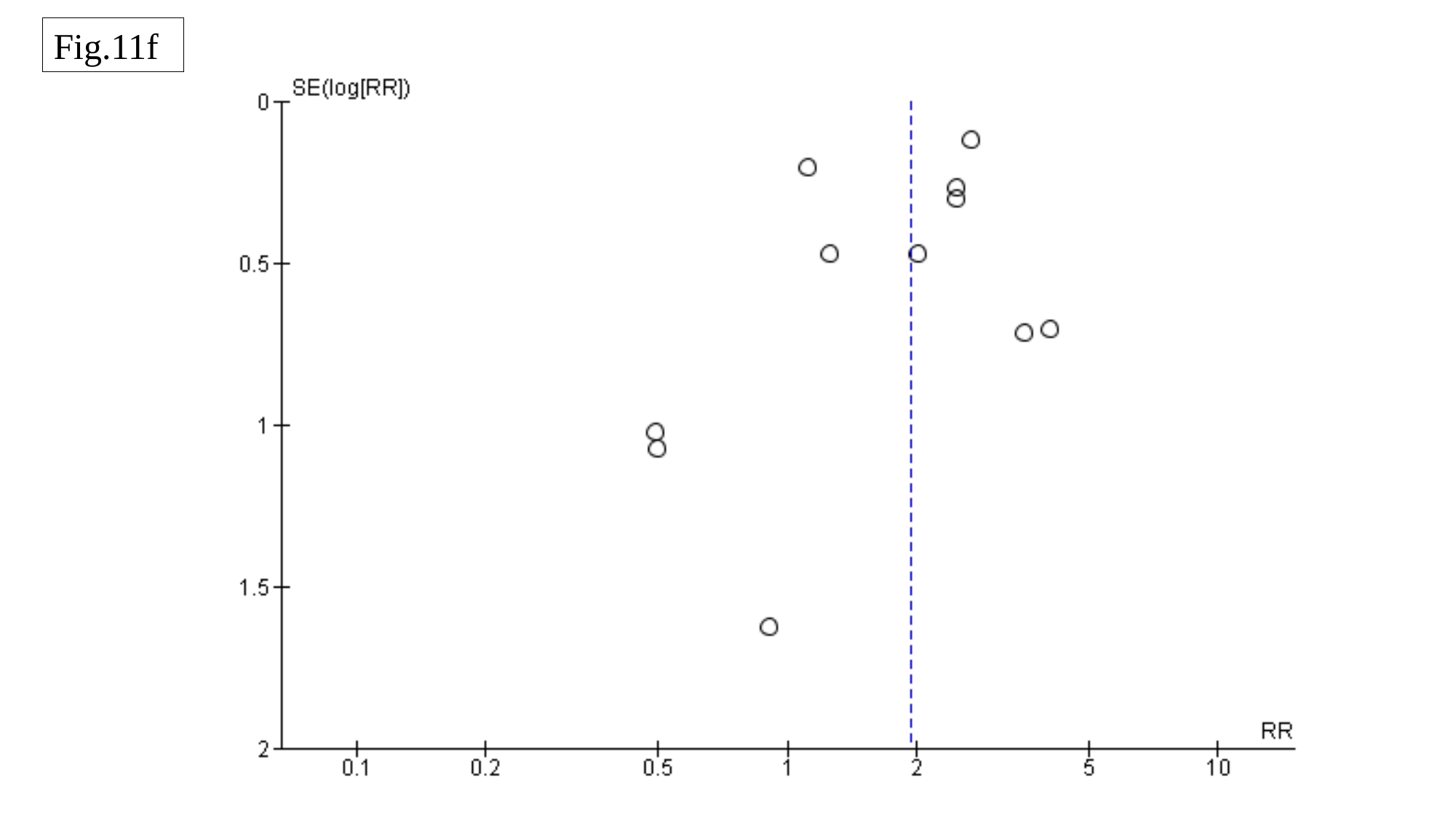

Fig.11f

## Slide 55
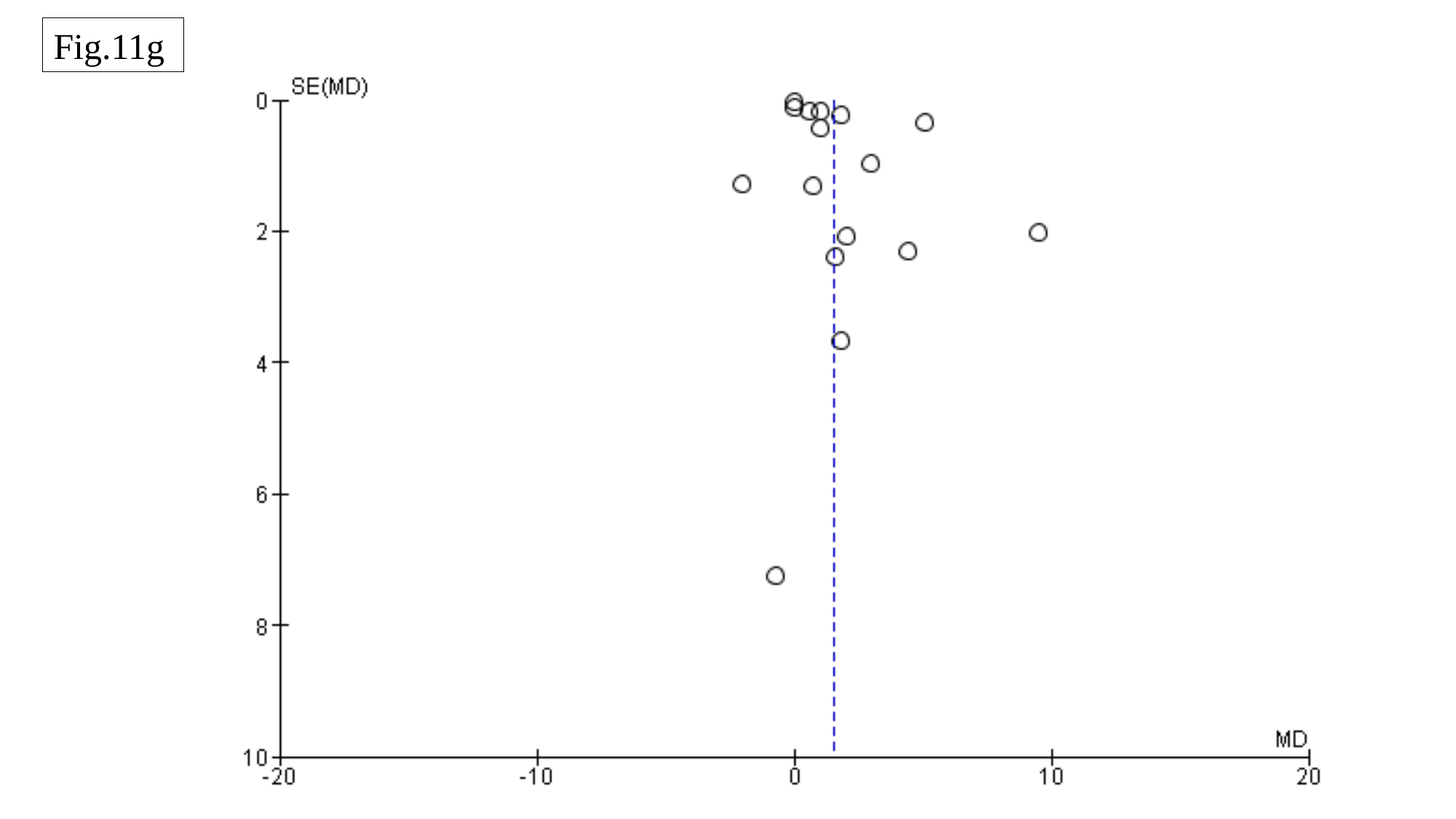

Fig.11g
