# Supplementary material for: Relationship Between GLIM‐Defined Malnutrition and Postoperative Outcomes After Curative Resection in Patients With Gastroenterological Cancer: Update Systematic Review and Meta‐Analysis
Source: Ann Gastroenterol Surg. 2026 Jan 27;10(2):348–62. doi: 10.1002/ags3.70173 (PMC12962040; doi:10.1002/ags3.70173)
Supplement: Supplementary file 3 — Appendix S2: The electronic database search strategy. Appendix S3: The trial registry search strategy. Appendix S4: Characteristics of studies excluded from the qualitative and quantitative synthesis. Appendix S5: Risk of bias for eligible studies using the Quality In Prognosis Studies tool. [file AGS3-10-348-s003.docx]

Appendix 2: The electronic database search strategy

**MEDLINE via PubMed**

#1. "Neoplasms"[Mesh] OR "carcinoma"[Mesh]

#2. Carcinom*[tiab] OR Cancer*[tiab] OR Neoplasm*[tiab] OR Malign*[tiab] OR tumo*[tiab]

#3. #1 OR #2

#4. "Global leadership initiative on malnutrition"[tiab] OR "GLIM"[tiab]

#5. #3 AND #4

**The cochrane central register of controlled trials (CENTRAL)**

#1. MeSH descriptor: [Neoplasms] explode all trees.

#2. MeSH descriptor: [Tumor] explodes all trees.

#3. MeSH descriptor: [Carcinoma] explodes all the trees.

#4. (neoplasm* OR tumor* OR carcinoma* OR cancer*):ti,ab,kw (word variations have been searched)

#5. #1 OR #2 OR #3 OR #4

#6. (“Global leadership initiative on malnutrition” OR “GLIM”):ti,ab,kw (Word variations have been searched)

#7. #5 AND #6

**EMBASE** **via ProQuest**

S1 EMB.EXACT.EXPLODE("neoplasm") OR EMB.EXACT.EXPLODE("carcinoma") OR (EMB.EXACT.EXPLODE("Cancer (crab)"))

S2 (ab(neoplasm* OR tumor* OR carcinoma* OR cancer*) OR ti(neoplasm* OR tumor* OR carcinoma* OR cancer*))

S3 S1 OR S2

S4 (ab("Global leadership initiative on malnutrition" OR "GLIM") OR ti("Global leadership initiative on malnutrition" OR "GLIM"))

S5 S3 AND S4

Appendix 3: The trial registry search strategy

**The world health organization international clinical trials platform search portal (WHO ICTRP)**

#1 Title (Global leadership initiative on malnutrition OR GLIM)

**ClinicalTrials.gov**

#1 Condition or disease (Global leadership initiative on malnutrition OR GLIM)

Appendix 4. Characteristics of studies excluded from the qualitative and quantitative synthesis

| **Study** | **Reason for exclusion** |
| --- | --- |
| Fierini D, et al. Clinical Nutrition Experimental 2020; 32: 23529393. | Wrong population |
| Xu JY, et al. Risk Manag Healthc Policy 2020;13:761-70. | Wrong population |
| Cereda E, et al. Clin Nutr 2021;40:3901-7. | Wrong exposure |
| IRCT20211027052884N1. | Wrong exposure |
| KCT0005529. | Wrong exposure |
| Movahed S, et al. Clin Nutr 2021;40:4449-55. | Wrong exposure |
| Ogata T, et al. Journal of Clinical Oncology 2021; 39:15. | Wrong exposure |
| Higashiguchi T, et al. Japanese Journal of Cancer and Chemotherapy 2020;47:1552-8. | Others |
| Abdel LO, et al. Clin Nutr 2019; 38:S262. | Insufficient data for outcome |
| Abdel LO, et al. Clin Nutr 2019; 38:S261-2. | Insufficient data for outcome |
| Azman M, et al. Int J Gerontol 2021; 15:266-71. | Insufficient data for outcome |
| Brumen BA, et al. Clin Nutr ESPEN 2020; 40:547-8. | Insufficient data for outcome |
| Chen X, et al. Chinese Journal of Clinical Nutrition 2020; 28:201-6. | Insufficient data for outcome |
| De LDL, et al. Clin Nutr ESPEN 2021; 46:S775-6. | Insufficient data for outcome |
| de Sousa MJP, et al. Ann Oncol 2021; 32:S817. | Insufficient data for outcome |
| Ehrsson YT, et al. Nutrients 2021; 13:1167. | Insufficient data for outcome |
| Einarsson S, et al. Clin Nutr ESPEN 2020; 40:149-55. | Insufficient data for outcome |
| Einarsson S, et al. Clin Nutr ESPEN 2020; 37:100-6. | Insufficient data for outcome |
| Garcia GC, et al. Clin Nutr ESPEN 2021; 46:S599. | Insufficient data for outcome |
| Gascón-Ruiz M, et al. Clin Nutr 2021; 40:3741-7. | Insufficient data for outcome |
| Gascón-Ruiz M, et al. Eur J Clin Nutr 2022; 76:698-702. | Insufficient data for outcome |
| Henriksen C, et al. Clin Nutr 2022; 41:329-36. | Insufficient data for outcome |
| Kaźmierczak-Siedlecka K, et al. Nutr Hosp 2020; 37:1179-85. | Insufficient data for outcome |
| Kiss N, et al. Clin Nutr 2022; 41:1102-11. | Insufficient data for outcome |
| Kiss N, et al. Clin Nutr ESPEN 2021; 46: S566-7. | Insufficient data for outcome |
| Li Y, et al. Chinese Journal of Clinical Nutrition 2020; 28: 297-302. | Insufficient data for outcome |
| Liu C, et al. Front Nutr 2021; 8:774636. | Insufficient data for outcome |
| Song HN, et al. Jpn J Clin Oncol 2022;52 :466-74. | Insufficient data for outcome |
| Migdanis I, et al. Clin Nutr 2019; 38: S146-7. | Insufficient data for outcome |
| Mueller TC, et al. Medicine (Baltimore) 2020; 99:e23642. | Insufficient data for outcome |
| Muresan BT, et al. Clin Nutr ESPEN 2020; 40: 547. | Insufficient data for outcome |
| Nunez AM, et al. Ann Oncol 2020; 31: S1046. | Insufficient data for outcome |
| Poulter S, et al. Nutrients 2021; 13:2602. | Insufficient data for outcome |
| Qin L, et al. Nutr Cancer 2021; 73:2732-9. | Insufficient data for outcome |
| Riad A, et al. Br J Surg 2021; 108:11. | Insufficient data for outcome |
| Sánchez-Torralvo FJ, et al. Support Care Cancer 2022; 30:1607-13. | Insufficient data for outcome |
| Sánchez-Torralvo FJ, et al. Clin Nutr ESPEN 2020; 40: 437-8. | Insufficient data for outcome |
| Santos CA, et al. Clin Nutr ESPEN 2021; 46: S720-1. | Insufficient data for outcome |
| Santos I, et al. Clin Nutr 2021; 40:5486-93. | Insufficient data for outcome |
| Sobrini P, et al. Eur Geriatr Med 2021; 12:653-6. | Insufficient data for outcome |
| Steer B, et al. Nutrients 2020; 12:3493. | Insufficient data for outcome |
| Tobberup R, et al. Clin Nutr ESPEN 2020; 40: 565-6. | Insufficient data for outcome |
| Valeriani L, et al. Clin Nutr ESPEN 2021; 46: S609-10. | Insufficient data for outcome |
| Van Der Meij BS, et al. Clin Nutr 2019; 38: S132. | Insufficient data for outcome |
| Wan M, et al. Nutr Cancer 2022:1-10. | Insufficient data for outcome |
| Wang P, et al. Dis Esophagus 2021; 34:54. | Insufficient data for outcome |
| Wang Y, et al. Nutrients 2021; 13:2744. | Insufficient data for outcome |
| Yin L, et al. JPEN J Parenter Enteral Nutr 2021; 45:1736-48. | Insufficient data for outcome |
| Zhang K, et al. Clin Nutr ESPEN 2020; 40:558. | Insufficient data for outcome |
| Zhang L, et al. Clin Nutr ESPEN 2020; 40:480. | Insufficient data for outcome |
| Zhang T, et al. Chinese Journal of Clinical Nutrition 2020; 28: 144-50. | Insufficient data for outcome |
| Zhang Z, et al. Nutrition. 2021; 83:111072. | Insufficient data for outcome |
| Zhou LP, et al. Nutrition 2021; 84:111044. | Insufficient data for outcome |
| Zhuang B, et al. Chinese Journal of Clinical Nutrition 2020; 28: 207-13. | Insufficient data for outcome |
| Chen WZ, et al. Eur J Surg Oncol. 2023; 49: 376-83. | Wrong exposure |
| Gao X, et al. Nutrition. 2022; 102: 111748. | Wrong exposure |
| GlobalSurg Collaborative and NIHR Global Health Unit on Global Surgery. Lancet Glob Health. 2023; 11: e341-9. | Wrong exposure |
| Soria-Utrilla V, et al. Int J Environ Res Public Health. 2022; 19: 13548. | Wrong study design |
| Wang L, et al. Front Nutr. 2023; 10: 1135854. | Wrong study design |
| Zhou L, et al. Front Nutr. 2023; 10: 1116243. | Wrong population |
| Takimoto M, et al. Nutrients. 2022; 14: 943. | Insufficient data for outcome |
| Cai W, et al. Front Nutr. 2022; 9: 995295. | Duplicate records |
| Chen WZ, et al. Front Nutr. 2022; 9: 960670. | Duplicate records |
| Matsui R, et al. Surg Today. 2023; 53: 578-87. | Duplicate records |
| Matsui R, et al. J Clin Med. 2023; 12: 1579. | Duplicate records |
| Song HN, et al. Jpn J Clin Oncol. 2022; 52: 466-74. | Duplicate records |
| Xu LB, et al. Front Oncol. 2022; 12: 851091. | Duplicate records |

Appendix 5: Risk of bias for eligible studies using the Quality In Prognosis Studies tool

| **Exposure** | **Outcome** | **Author (published year)** | **Bias domains** | | | | | |
| --- | --- | --- | --- | --- | --- | --- | --- | --- |
|  |  |  | **Study participation** | **Study attrition** | **Prognostic factor measurement** | **Outcome measurement** | **Study confounding** | **Statistical analysis and reporting** |
| **GLIM malnutrition** | **Overall survival** | **Huang DD (2022) [31]** | Low | High | Low | Low | Moderate | Low |
|  |  | **Lee B (2021) [33]** | Moderate | High | High | High | High | High |
|  |  | **Okada G (2021) [34]** | Low | Moderate | Moderate | Low | Moderate | Low |
|  |  | **Wang P (2021) [35]** | Low | High | Low | Low | Moderate | Low |
|  |  | **Xu LB (2021) [36]** | Low | High | Low | Low | Moderate | Low |
|  |  | **Matsui R (2022) [39]** | Low | High | Moderate | Low | Moderate | Moderate |
|  |  | **Murnane LC (2023) [41]** | Low | High | Low | Low | Moderate | Moderate |
|  |  | **Okazoe Y (2023) [42]** | Low | High | Moderate | Low | Moderate | Moderate |
|  |  | **Zhang Y (2022) [45]** | Low | Low | Low | Low | Moderate | Moderate |
|  |  | **Zheng HL (2022) [46]** | Low | High | Moderate | Low | Moderate | Low |
|  |  | **Omiya S (2023) [48]** | Low | High | Low | Low | Moderate | Moderate |
|  |  | **Harimoto N (2023) [49]** | Low | High | Low | High | Moderate | Moderate |
|  |  | **Shen N (2023) [50]** | Low | High | Moderate | Low | Moderate | Moderate |
|  |  | **Zhou CJ (2023) [51]** | Low | High | Low | Low | Moderate | Moderate |
|  |  | **Chen W (2024) [53]** | Low | High | Low | Low | Moderate | Moderate |
|  |  | **Wang SL (2024) [54]** | Low | High | Low | Low | Moderate | Moderate |
|  |  | **Igarashi T (2024) [55]** | Moderate | High | Low | Low | Moderate | Moderate |
|  |  | **Luo X (2024) [57]** | Low | High | Low | High | Moderate | Moderate |
|  | **Relapse-free survival** | **Huang DD (2021) [30]** | Low | High | Low | Low | Moderate | Low |
|  |  | **Wang P (2021) [35]** | Low | High | Low | Low | Moderate | Low |
|  |  | **Matsui R (2022) [40]** | Low | High | Moderate | Low | Moderate | Moderate |
|  |  | **Okazoe Y (2023) [42]** | Low | High | Moderate | Low | Moderate | Moderate |
|  |  | **Zhang Y (2022) [45]** | Low | Low | Low | Low | Moderate | Moderate |
|  |  | **Omiya S (2023) [48]** | Low | High | Low | Low | Moderate | Moderate |
|  |  | **Harimoto N (2023) [49]** | Low | High | Low | High | Moderate | Moderate |
|  |  | **Chen W (2024) [53]** | Low | High | Low | Low | Moderate | Moderate |
|  |  | **Igarashi T (2024) [55]** | Moderate | High | Low | Low | Moderate | Moderate |
|  | **Postoperative complications** | **Huang DD (2021) [30]** | Low | Low | Low | Low | Moderate | Low |
|  |  | **Kakavas S (2020) [32]** | Low | Low | Moderate | Low | High | Moderate |
|  |  | **Okada G (2021) [34]** | Low | Low | Moderate | Low | Moderate | High |
|  |  | **Wang P (2021) [35]** | Low | Low | Low | Low | Moderate | High |
|  |  | **Xu LB (2021) [36]** | Low | Low | Low | Low | Low | Low |
|  |  | **Yin L (2021) [37]** | Low | Low | Low | Low | Moderate | Moderate |
|  |  | **Liu Y (2023) [38]** | Low | Low | Low | Low | Moderate | Moderate |
|  |  | **Matsui R (2022) [39]** | Low | Low | Moderate | Low | Moderate | Moderate |
|  |  | **Murnane LC (2023) [41]** | Low | Low | Low | Low | Moderate | Moderate |
|  |  | **Okazoe Y (2023) [42]** | Low | Low | Moderate | Low | Moderate | Moderate |
|  |  | **Tan S (2022) [43]** | Low | Low | Moderate | Low | Moderate | Moderate |
|  |  | **Wobith M (2022) [44]** | Low | Low | Low | Low | Moderate | Moderate |
|  |  | **Omiya S (2023) [48]** | Low | Low | Low | High | Moderate | Moderate |
|  |  | **Harimoto N (2023) [49]** | Low | Low | Low | Low | Moderate | Moderate |
|  |  | **Shen N (2023) [50]** | Low | Low | Moderate | Low | Moderate | Moderate |
|  |  | **Zhou CJ (2023) [51]** | Low | Low | Low | High | Moderate | Moderate |
|  |  | **Sun S (2023) [52]** | Low | Low | Low | Low | Moderate | Moderate |
|  |  | **Chen W (2024) [53]** | Low | Low | Low | Low | Moderate | Moderate |
|  |  | **Wang SL (2024) [54]** | Low | Low | Low | High | Moderate | Moderate |
|  |  | **Igarashi T (2024) [55]** | Low | Low | Low | High | Moderate | Moderate |
|  |  | **Yıldız Kopuz TN (2024) [56]** | Low | Low | Low | Low | Moderate | Moderate |
|  | **Postoperative hospital stays** | **Lee B (2021) [33]** | Moderate | High | High | High | High | High |
|  |  | **Okada G (2021) [34]** | Low | Low | Moderate | Moderate | Moderate | Moderate |
|  |  | **Wang P (2021) [35]** | Low | Low | Low | Moderate | Moderate | Moderate |
|  |  | **Xu LB (2021) [36]** | Low | Low | Low | Moderate | Moderate | Moderate |
|  |  | **Yin L (2021) [37]** | Low | Low | Low | Moderate | Moderate | Moderate |
|  |  | **Liu Y (2023) [38]** | Low | Low | Low | Moderate | Moderate | Moderate |
|  |  | **Murnane LC (2023) [41]** | Low | Low | Low | Low | Moderate | Moderate |
|  |  | **Okazoe Y (2023) [42]** | Low | Low | Moderate | Moderate | Moderate | Moderate |
|  |  | **Tan S (2022) [43]** | Low | Low | Moderate | Low | Moderate | Moderate |
|  |  | **Wobith M (2022) [44]** | Low | Low | Low | Moderate | Moderate | Moderate |
|  |  | **Omiya S (2023) [48]** | Low | Low | Low | High | Moderate | Moderate |
|  |  | **Zhou CJ (2023) [51]** | Low | Low | Low | High | Moderate | Moderate |
|  |  | **Sun S (2023) [52]** | Low | Low | Low | High | Moderate | Moderate |
|  |  | **Chen W (2024) [53]** | Low | Low | Low | High | Moderate | Moderate |
|  |  | **Wang SL (2024) [54]** | Low | High | Low | High | Moderate | Moderate |
|  |  | **Yıldız Kopuz TN (2024) [56]** | Low | Low | Low | High | Moderate | Moderate |
